# Pyrenyl-Substituted Imidazo[4,5-*f*][1,10]phenanthroline Rhenium(I) Complexes with Record-High Triplet Excited-State Lifetimes at Room Temperature: Steric Control of Photoinduced Processes in Bichromophoric Systems

**DOI:** 10.1021/acs.inorgchem.3c02662

**Published:** 2023-11-11

**Authors:** Katarzyna Choroba, Mateusz Penkala, Joanna Palion-Gazda, Ewa Malicka, Barbara Machura

**Affiliations:** Institute of Chemistry, University of Silesia, Szkolna 9, Katowice 40-006, Poland

## Abstract

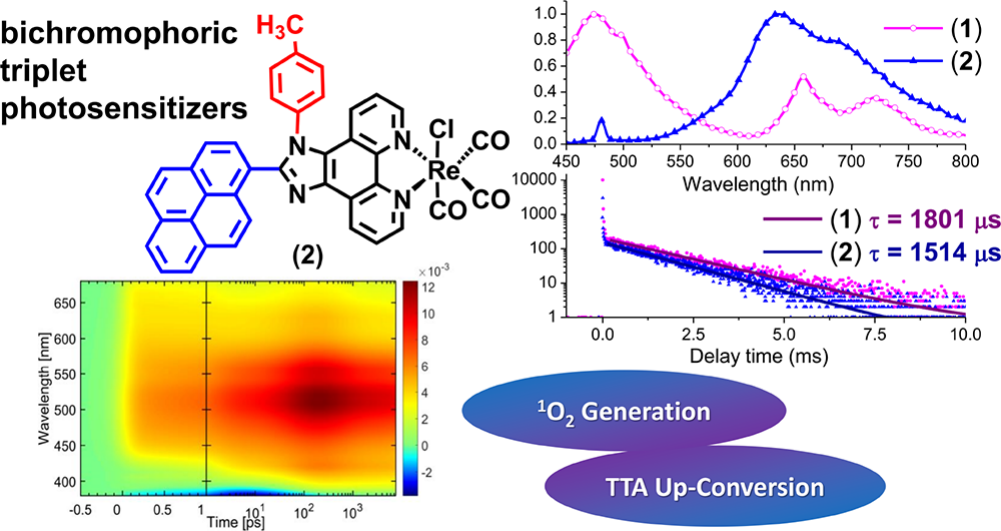

Photochemical applications based on intermolecular photoinduced energy triplet state transfer require photosensitizers with strong visible absorptivity and extended triplet excited-state lifetimes. Using a bichromophore approach, two Re(I) tricarbonyl complexes with 2-(1-pyrenyl)-1*H*-imidazo[4,5-*f*][1,10]phenanthroline (pyr-imphen) and 1-(4-(methyl)phenyl)-2-(1-pyrenyl)-imidazo[4,5-*f*][1,10]phenanthroline (pyr-tol-imphen) showing extraordinary long triplet excited states at room temperature (>1000 μs) were obtained, and their ground- and excited-state properties were thoroughly investigated by a wide range of spectroscopic methods, including femtosecond transient absorption (fs-TA). It is worth noting that the designed [ReCl(CO)_3_(pyr-imphen)] (**1**) and [ReCl(CO)_3_(pyr-tol-imphen)] (**2**) complexes form a unique pair differing in the mutual chromophore arrangement due to introduction of a 4-(methyl)phenyl substituent into the imidazole ring at the H1-position, imposing an increase in the dihedral angle between the pyrene and {ReCl(CO)_3_(imphen)} chromophores. The magnitude of the electronic coupling between the pyrene and {ReCl(CO)_3_(imphen)} chromophores was found to be an efficient tool to tune the photophysical properties of **1** and **2**. The usefulness of designed Re(I) compounds as triplet photosensitizers was successfully verified by examination of their abilities for ^1^O_2_ generation and triplet–triplet annihilation upconversion. The phosphorescence lifetimes, ∼1800 μs for **1** and ∼1500 μs for **2**, are the longest lifetimes reported for Re(I) diimine carbonyl complexes in solution at room temperature.

## Abstract 2

Two Re(I)-based emitters with extraordinary long triplet excited states at room temperature were obtained and thoroughly explored with the aid of static and time-resolved spectroscopic methods, including transient absorption. Photoinduced processes in the bichromophore Re(I) complexes with imidazophenanthroline-based ligands were additionally modified by the substituent steric hindrance in the imidazole ring at the H1-position. The usefulness of Re(I) compounds as triplet photosensitizers was successfully verified by examination of their abilities for ^1^O_2_ generation and triplet−triplet annihilation upconversion.

## Introduction

Precise control of electronic excited states in transition metal complexes, and thus their photophysical and photochemical properties, is crucial for rational design of functional materials with predefined photophysical behavior, appropriate for applications in photocatalysis,^1−4^ organic light-emitting diodes,^5−9^ solar energy conversion,^10−12^ phosphorescence molecular sensing,^13−15^ and chemotherapy and photodynamic therapy. Some of these photochemical applications involve intermolecular photoinduced energy triplet state transfer, with the efficiency being strongly dependent on the visible absorptivity and triplet excited-state lifetime of the photosensitizer. To increase the visible light harvesting of transition metal complexes, ligands with strong visible absorptivity are introduced into the coordination sphere of the metal ion. In turn, to achieve triplet emitters with long excited states, both radiative and nonradiative decay rate constants must be small, which means that the triplet excited state should have a predominant ligand character.^13,16−26^ An efficient strategy for accessing long-lived phosphorescent dyes is to use an organic ligand with a non-emissive long-lived triplet state (^3^IL) close in energy to an emissive metal-to-ligand charge transfer triplet excited state (^3^MLCT). The establishment of thermal equilibration between these triplet states results in an extension of the lifetime of ^3^MLCT, as the ligand-based triplet excited state becomes the energy reservoir. Extended emission lifetimes are also expected when the ^3^IL excited state is noticeably lower than ^3^MLCT, and strong spin–orbit coupling results in the ligand-based phosphorescence at room temperature. Since when Ford and Rodgers first demonstrated a bichromophoric approach,^27^ it has been successfully used for the synthesis of rhenium(I) carbonyl complexes with naphthalimide-subsitiuted ligands^28−30^ and ruthenium(II) complexes bearing polypyridyl ligands functionalized with pyrenyl and anthryl groups,^19,31−36^ giving promising photosensitizers. Recently, our group has explored Re(I) carbonyl complexes with aryl-substituted 2,2′:6′,2″-terpyridines and reported extended room-temperature photoluminescence lifetime for [ReCl(CO)_3_(4′-pyrenyl-terpy-κ^2^N)].^37^

In the current contribution, we present photophysical properties of bichromophore Re(I) carbonyl complexes with 2-(1-pyrenyl)-1*H*-imidazo[4,5-*f*][1,10]phenanthroline (pyr-imphen) and 1-(4-(methyl)phenyl)-2-(1-pyrenyl)-imidazo[4,5-*f*][1,10]phenanthroline (pyr-tol-imphen). The great advantages of imidazo[4,5-*f*][1,10]phenanthrolines are their prominent antitumor and luminescence properties, as well as the convenient synthesis along with the possibility of introduction of all types of substituents into the imidazole ring at H1- and C2-positions, directly (via the covalent bond) or through an aryl/heterocycle bridge.^38,39^ Although imidazo[4,5-*f*][1,10]phenanthrolines have been combined with various transition metal ions,^38,39^ [ReCl(CO)_3_(R-imphen)] systems have been rarely investigated, and their photophysics is unfathomable.^40−53^

Furthermore, the designed complexes [ReCl(CO)_3_(pyr-imphen)] (**1**) and [ReCl(CO)_3_(pyr-tol-imphen)] (**2**) form a unique pair, making it possible to determine the impact of ligand conformation restriction on the mutual chromophore arrangement and, thus, modification of photoinduced processes and triplet–triplet energy processes in such systems. The 4-(methyl)phenyl substituent, introduced into the imidazole ring at the H1-position, imposes an increase in the dihedral angle between the pyrene and [ReCl(CO)_3_(imphen)] chromophores and thus induces a weaker electronic coupling between the organic and metal-based chromophores. To the best of our knowledge, this type of a ligand conformation restriction has been first used to tune photophysical achievements of bichromophore transition metal complexes with imidazophenanthroline-based ligands. Taking into consideration a wide range of possible structural modifications of imphen-based ligands, it can be assumed that this approach will be successfully utilized in the design of other multichromophore transition-metal compounds. An important advantage of the attachment of aryl and alkyl groups concerns the increase in the solubility of resulting transition metal complexes, which is beneficial regarding the applications of these systems.

**Chart 1:**
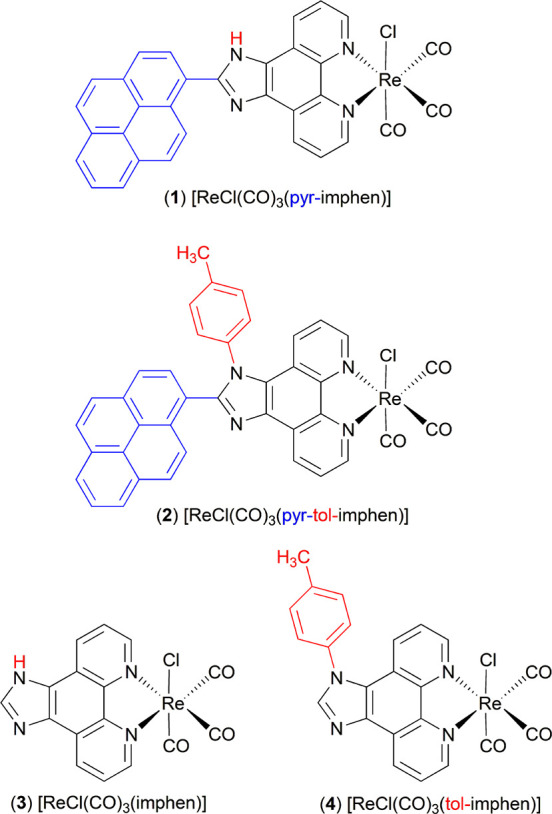
Rhenium(I) Complexes Employed in the Study

To get a better understanding of the impact of the pyrene chromophore and molecular ligand conformation, ground- and excited-state properties of [ReCl(CO)_3_(pyr-imphen)] (**1**) and [ReCl(CO)_3_(pyr-tol-imphen)] (**2**) were compared with those for parent complexes [ReCl(CO)_3_(imphen)] (**3**) and [ReCl(CO)_3_(tol-imphen)] (**4**) (Chart 1).

A complete picture of excited-state processes in the designed systems has been achieved with the aid of static and time-resolved spectroscopic methods, including transient absorption (TA). In addition, both pyrenyl-substituted Re(I) complexes, which showed extraordinary long triplet excited states at room temperature (>1000 μs), were successfully tested as photosensitizers for ^1^O_2_ sensitizing and triplet–triplet annihilation for energy upconversion (TTA UC) to verify their potential applications.

## Results and Discussion

### Synthesis and Structure Confirmation

Complexes [ReCl(CO)_3_(pyr-imphen)] (**1**), [ReCl(CO)_3_(pyr-tol-imphen)] (**2**), [ReCl(CO)_3_(imphen)] (**3**), and [ReCl(CO)_3_(tol-imphen)] (**4**) were synthesized by a direct reaction of [Re(CO)_5_Cl] with the appropriate imidazo[4,5-*f*][1,10]phenanthroline-based ligand in refluxing acetonitrile-toluene mixture under argon protection. Regarding our previous results,^37,53^ compounds **1** and **2** have been designed as bichromophore systems with metal-to-ligand charge transfer (^3^MLCT) and pyrene-localized (^3^IL_pyrene_) triplet excited states in energetic proximity. Complexes **3** and **4** were used as reference systems for a better understanding of photoinduced processes in pyrenyl-substituted Re(I) systems.

Successful coordination of the imidazo[4,5-*f*][1,10]phenanthroline-based ligand and formation of Re(I) carbonyls **1**–**4** were evidenced with the aid of elemental analyses and spectroscopic tools (see Figures S2–S6 in the Supporting Information). FT-IR spectra of **1**–**4** show a set of intense ν(C≡O) bands in the region of 2030–1875 cm^–1^ with a pattern typical of *fac*-{Re(CO)_3_}^+^ moiety accompanied by less intensive stretching and bending vibrations of the coordinated imidazo[4,5-*f*][1,10]phenanthroline-based ligand (Figure S2). The ^1^H and ^13^C NMR signals of model chromophores were fully assigned using one- and two-dimensional ^1^H–^1^H COSY, ^1^H–^13^C HMQC, and ^1^H–^13^C HMBC techniques. For the pyrenyl-substituted Re(I) systems (**1** and **2**), however, the full assignment of the signals in ^1^H and ^13^C NMR spectra was precluded due to the significant overlapping of signals (Figures S3 and S4).

**Figure 1:**
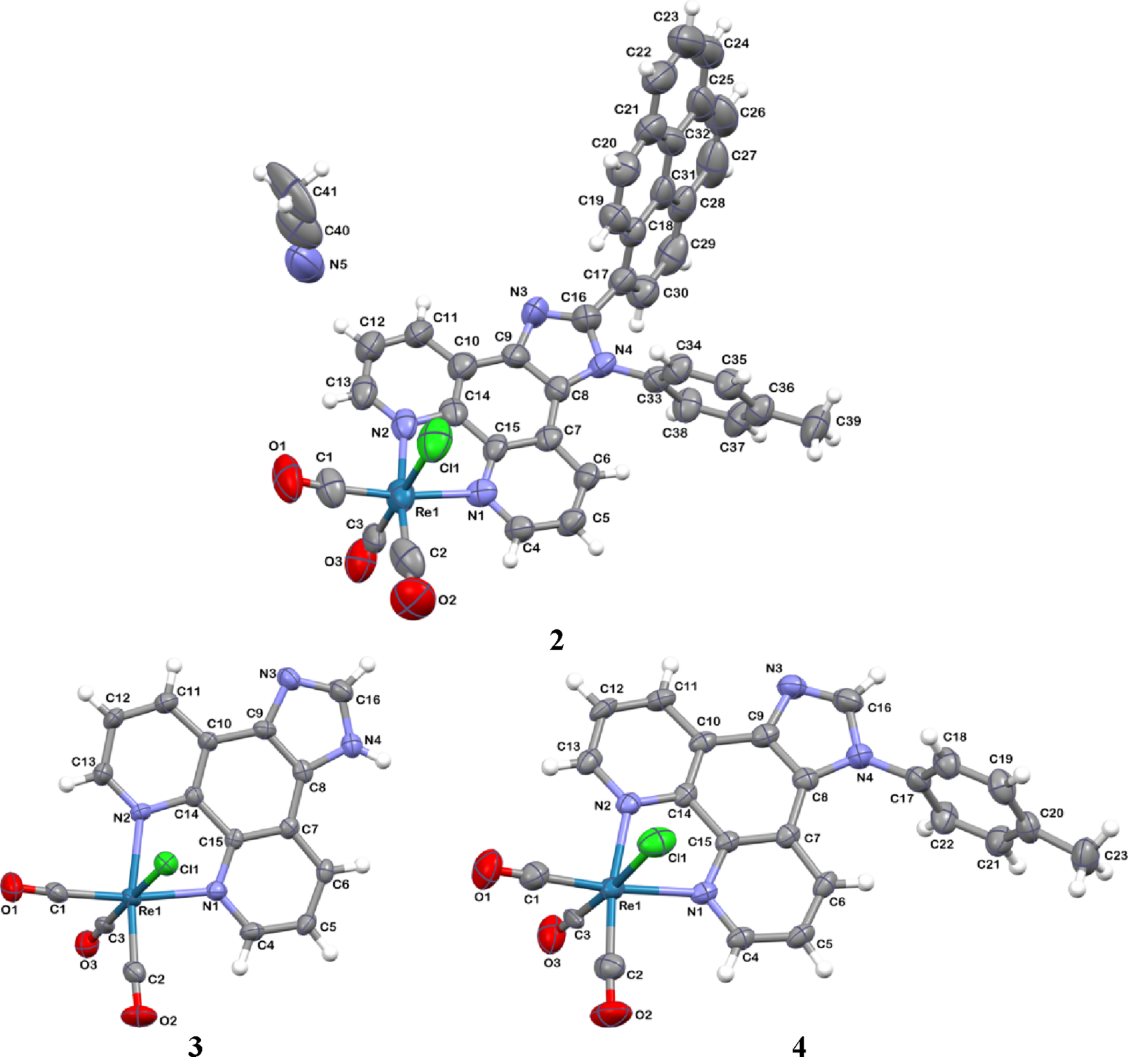
Perspective view showing the asymmetric units of Re(I) complexes along with the atom numbering. Displacement ellipsoids are drawn at the 50% probability level.

Also, single-crystal X-ray structures of complexes **2**–**4** were determined (Tables S1–S5, Figure 1, and Figures S1, S7, and S8). Typically, of this class of compounds, the Re(I) ion of **2**–**4** shows a distorted octahedral coordination environment, and three carbonyl ligands are arranged in *fa*c geometry (Figure 1). The introduction of aryl substituents (pyrenyl and 4-(methyl)phenyl) into the imidazole ring at the C2- and H1-positions of the imphen core does not generate noticeable changes in bond lengths and angles around the Re(I) center (Table S2). Most remarkably, due to the steric hindrance induced by the 4-(methyl)phenyl substituent attached to the imidazole ring at the H1-position, there is a large torsional twist (∼70°) between the planes of imphen and pyrene in the molecular structure **2**, which may lead to weak electronic communications between ^3^MLCT and ^3^IL_pyrene_ excited states.

The packing and stabilization of the crystal structures of **2**–**4** are contributed by weak hydrogen bonds as well as π•••π and X–Y•••π intermolecular contacts (Figures S7 and S8 and Tables S3–S5).

### Theoretical Insight on Ground- and Excited-State Properties of **1**–**4**

To better understand the effect of the pyrenyl substituent and its inclination toward the imphen core on optical features of resulting Re(I) complexes, DFT and TD-DFT calculations were performed for **1**–**4** at the PBE0/def2-TZVPD/def2-TZVP level of theory (Figures S9–S18 and Tables S6–S10).

The calculated bond lengths and angles of **1**–**4** in ground-state geometries (S_0_) are in satisfactory agreement with X-ray analysis results, and general trends observed in the experimental data are well theoretically reproduced, as shown in Figure S9 and Table S6. Upon going from S_0_ to T_1_, noticeable variations in bond distances were found only in the case of the parent complexes. In the triplet-optimized geometry (T_1_) of **3** and **4**, Re–N and Re–Cl distances undergo shortening, and Re–C becomes elongated. The noticeable structural difference between the **1** and **2** complexes is only the pyrene-imphen torsion angle, which was estimated to be ∼39° and ∼61° for ground state (S_0_)-optimized geometries of **1** and **2**, respectively. In the triplet-optimized geometries, the pyrene-imphen dihedral angle decreases to ∼20° for **1** and ∼52° for **2** (Table S6). The calculated torsion angle values clearly indicate that the twist of the aryl group toward the imphen core, and thus the magnitude of their electronic coupling, can be effectively controlled by substituents of suitable steric hindrance introduced into the imidazole ring at the H1-position. It may be an efficient way of tuning the photophysical properties of these systems.

**Figure 2:**
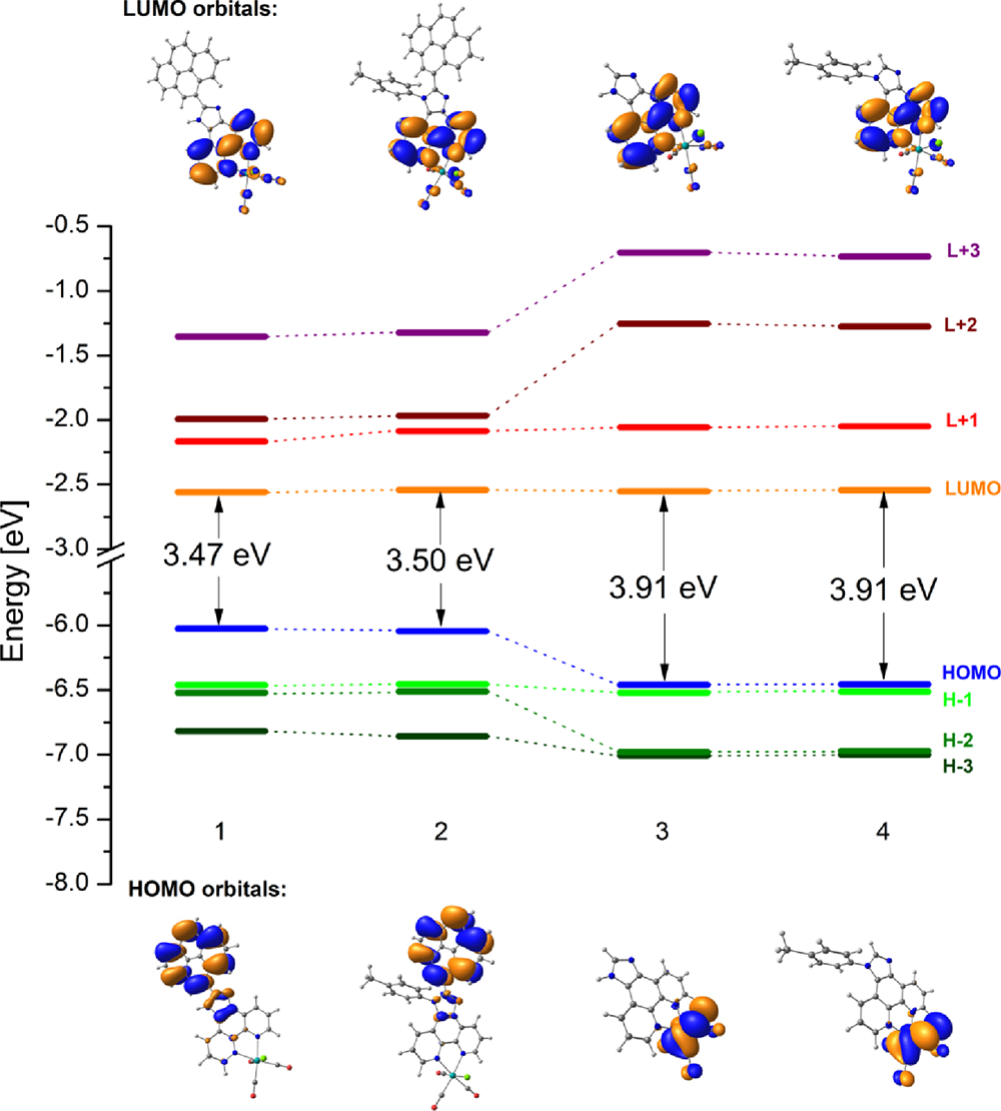
Partial molecular orbital energy level diagrams with the electron density plots of the highest occupied and lowest unoccupied MOs for complexes **1**–**4**.

As shown in Figure 2, the pyrenyl-substituted systems show a reduced HOMO–LUMO gap relative to the reference complexes. The attachment of pyrene to the imphen core leads to a ∼0.5 eV destabilization of the HOMO of complexes **1** and **2** in relation to the parent systems, leaving the LUMO energy virtually unperturbed. In all complexes, the lowest unoccupied molecular orbital (LUMO) is predominately constituted of π* imphen ligand orbitals. The highest occupied molecular orbital (HOMO) of **1** and **2** resides almost exclusively on the pyrenyl substituent, contrary to the parent complexes with HOMO spreading over the {Re(CO)_3_Cl} moiety. Rhenium *d* orbitals combined with π*_CO_ and p_Cl_ orbitals participate in H-1 and H-2 of **1**–**2** and H-1 and H-3 of **3**–**4**. A noticeable stabilization of L + 2 and L + 3 of complexes **1** and **2** in relation to model systems is attributed to the large contribution of pyrene orbitals.

According to TD-DFT calculations and NBO analysis (Figures S15 and S18 and Tables S7 and S10), the striking difference between **1** and **2** concerns the lowest energy singlet transition. For complex **2**, the transition S_0_ → S_1_ (exclusively contributed by H-1 → L) is MLCT in nature with a low oscillator strength. The hole is localized on rhenium *d*_π_ orbitals, and the particle is constituted by π* imphen ligand orbitals. Conversely, for the complex with a significantly smaller pyrene-imphen torsion angle (**1**), the lowest energy singlet transition displays mixed character: ^1^MLCT (d_π_(Re) → π*_imphen_) and ^1^ILCT (π_pyrene_ → π*_imphen_). No pure ^1^MLCT transitions was evidenced theoretically for **1**. For model chromophores, all three lowest energy singlet transitions, attributed to the long-wavelength absorption band, are MLCT character.

To achieve the characteristics of the lowest energy triplet excited state of **1**–**4** complexes, the spin density surfaces were generated from the lowest energy optimized triplet states (Figure S10). For both pyrenyl-substituted Re(I) complexes, the spin density surfaces are predominantly distributed on the pyrenyl substituent but they differ in the contribution of imphen orbitals. A larger imphen participation for complex **1** indicates a greater degree of intraligand charge transfer in the triplet state, assigned as ^3^IL_pyrene_ /^3^ILCT for **1** and ^3^IL_pyrene_ for **2**. The spin density of model chromophores, distributed among the {Re(CO)_3_Cl} unit and π* orbitals of the phen moiety, confirms the ^3^MLCT character of their triplet states.

### UV–Vis Absorption and Emission Spectra

**Figure 3:**
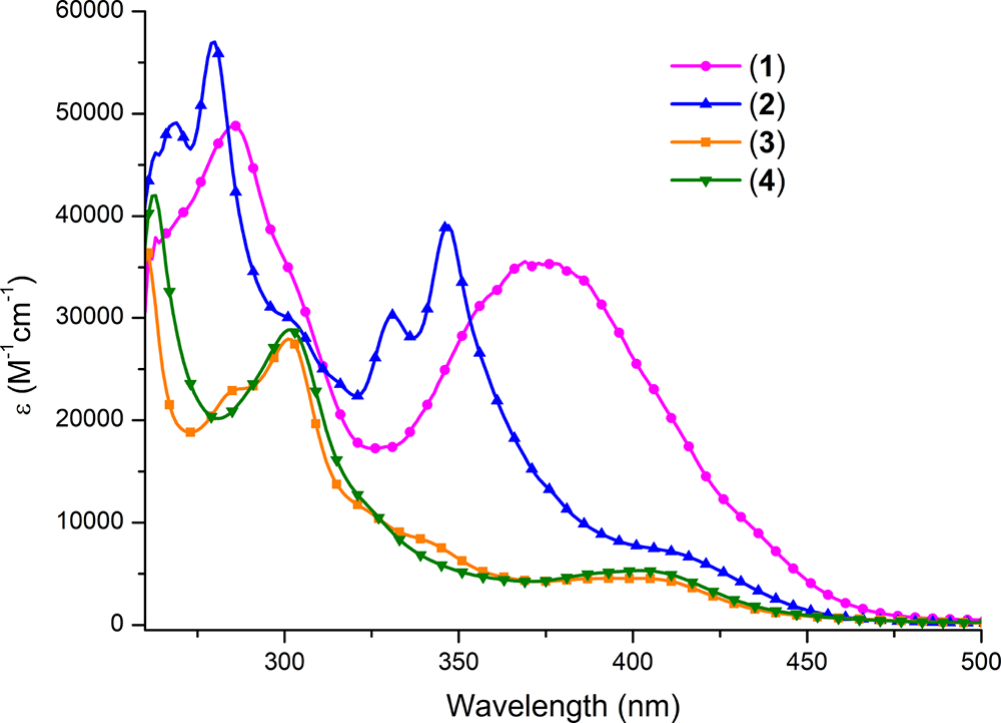
Comparison of UV–vis spectra of complexes **1**–**4** in DMSO. Concentration of compounds: 25 μM.

UV–vis spectra of Re(I) complexes (**1**–**4**) in DMSO are shown in Figure 3 and Figure S19–S22, and the absorption maxima along with corresponding molar extinction coefficients are summarized in Table S11.

The absorption bands of **1**–**4** in the high-energy region of 220–325 nm are ascribed to intraligand ^1^π → ^1^π* transitions of the corresponding imphen-based ligand. In the range of 325–475 nm, complex **1** shows broad and strong absorption with a maximum at ∼370 nm and a weak shoulder at ∼430 nm. By comparing with the spectra of pyr-imphen, imphen, and parent Re(I) complex (**3**), this band can be assigned to metal-to-ligand charge transfer (^1^MLCT, d_π_(Re) → π*_imphen_) and intraligand (^1^IL, π_pyrene_ → π*_pyrene_) in combination with intraligand charge transfer transitions (^1^ILCT, π_pyrene_ → π*_imphen_). The red-shift and significant absorptivity enhancement of the lowest energy absorption in relation to **3**, along with the lack of characteristic pyrene vibronic progression between 300 and 350 nm, support the strong electronic coupling between 1-pyrenyl and imphen moieties (see Figure S19). Complex **2** shows a drastically different absorption profile in this region. In this case, the absorption with characteristic vibronic progression corresponding to π_pyrene_–π_pyrene_* transitions (325–400 nm) is well resolved from the ^1^MLCT band, indicating the weak electronic coupling between 1-pyrenyl and imphen units. The observed spectral features of **1** and **2** well correlate with TD-DFT calculations, which showed that the incorporation of 4-(methyl)phenyl at the H1-position of 2-(1-pyrenyl)-1*H*-imidazo[4,5-*f*][1,10]phenanthroline leads to a significant increase in the dihedral angle between the appended pyrenyl group and the imphen framework and, thus, better separation of ^1^MLCT and ^1^IL states.

**Figure 4:**
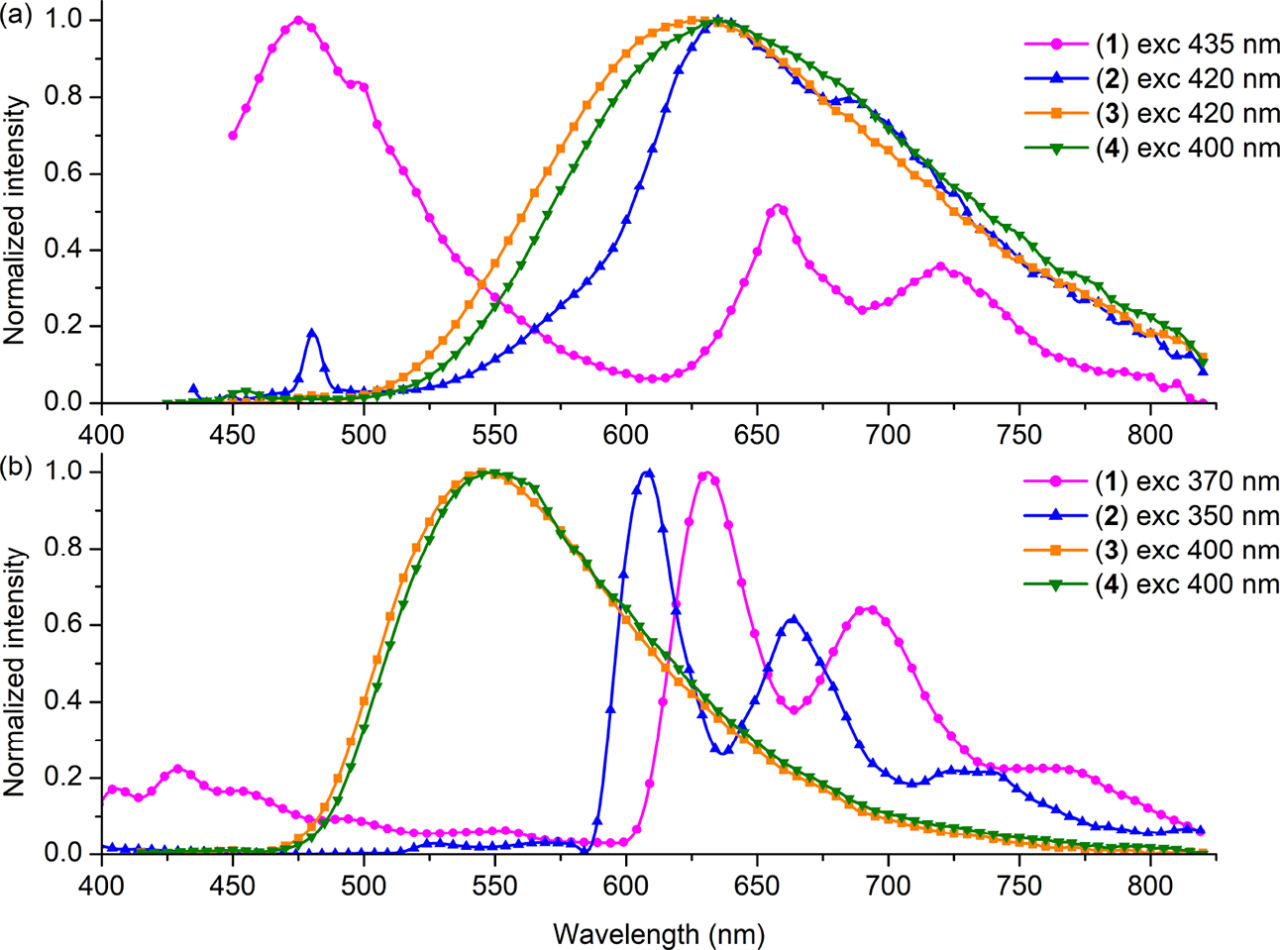
Emission spectra of **1**–**4** in DMSO at RT (a) and in an EtOH:MeOH glass matrix at 77 K (b).

Regarding the emission behavior, parent complexes show an excitation-independent broad and featureless emission band at room temperature (Figure 4 and Figures S23 and S24), which remains unstructured and undergoes a significant blue-shift upon cooling to 77 K, consistent with the emission being predominantly of triplet-state metal-to-ligand charge-transfer (^3^MLCT) character^54−56^ (Figure 4).

**Figure 5:**
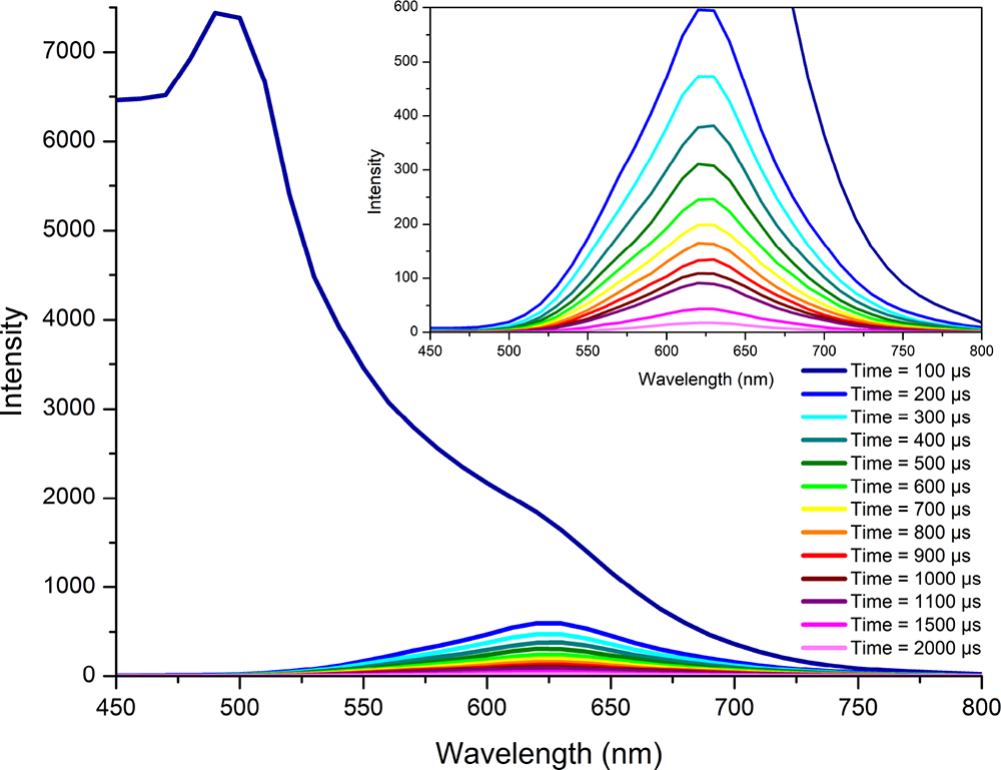
Time-resolved emission spectra (TRES) of **2** in DMSO. Concentration: 50 μM; excitation wavelength: 435 nm. Inset: close-up of low intensity spectra. For TRES spectra of **1** and excitation wavelength of 355 nm, see Figures S26 and S27 in the Supporting Information.

Distinctly from chromophores **3** and **4**, pyrenyl-substituted Re(I) complexes (**1** and **2**) display excitation-dependent dual fluorescence-phosphorescence emission (Figures S23 and S24). With the lower energy excitation, the relative phosphorescence–fluorescence increases. A short-lived higher energy component of **1** and **2** is superimposed with the ligand-centered fluorescence (^1^IL), but its intensity is significantly quenched relative to that of the corresponding free ligand (Figure S25). Such a phenomenon is well recognized in bichromophoric systems and indicates incomplete energy transfer from the ^1^IL to ^1^MLCT excited state via the Förster resonance energy transfer (FRET) mechanism.^29,30,57−59^ The values of *EnT* according to eq 1:
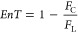
1where *F*_C_ and *F*_L_ are the integrated fluorescence of the complex and free ligand,^60,61^ equal to 98.76% for **1** and 98.92% for **2**. The stability and photostability of **1** and **2** in DMSO exclude the fact that the observed higher energy band is the emission of the ligand that has dissociated from the complex (Figures S21 and S22). The contribution of fluorescence and phosphorescence in emission spectra of **1** and **2** was also evidenced by time-resolved emission spectra (TRES) recorded at room temperature (Figure 5 and Figures S26 and S27).

A striking difference between **1** and **2** can be noticed at longer wavelengths (>500 nm). The phosphorescence band of **2** is almost structureless and falls in the range of ^3^MLCT for the parent chromophores. In turn, complex **1** exhibits a vibronically structured emission band with maxima at 657, 720, and 790 nm, attributable to the pyrene-based phosphorescence. The observation of the room-temperature phosphorescence of pyrene is very limited. Despite a few Ru(II) and Pt(II) examples showing pyrene-based phosphorescence at RT, pyrenyl-substituted transition complexes are generally characterized by a structureless ^3^MLCT emission band, likewise complex **2**, or they are non-emissive at RT.^62−69^

**Figure 6:**
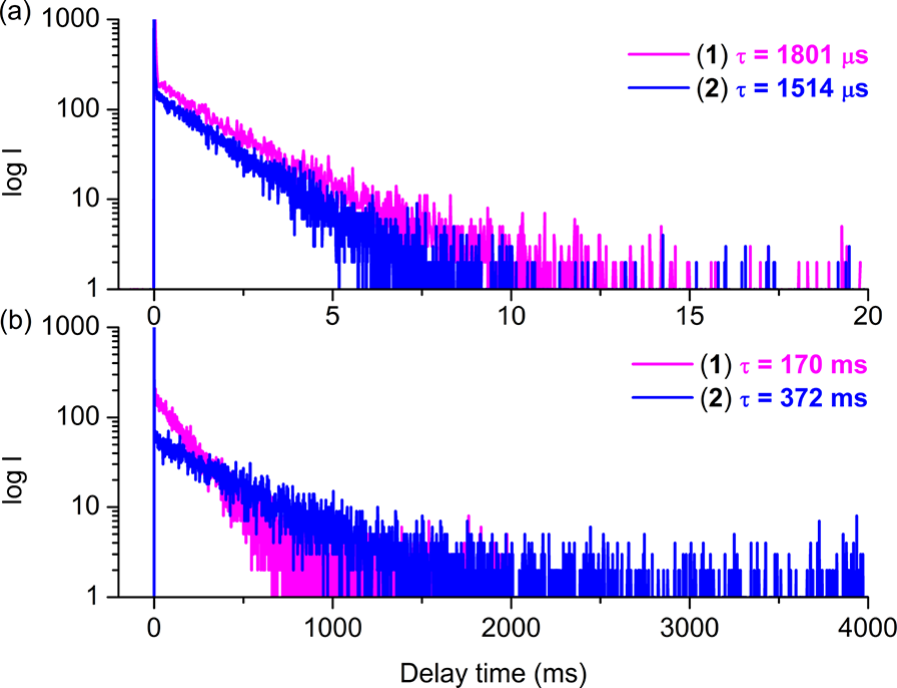
Comparison of decay time curves for **1** and **2** in DMSO at RT (a) and EtOH:MeOH glass matrix at 77 K (b).

For both complexes **1** and **2**, phosphorescence lifetimes at RT, determined using the monoexponential decay model (∼1800 μs for **1** and ∼1500 μs for **2**), are much longer than those of reference chromophores (223 ns for **3** and 179 ns for **4**, see Table 1 and Figure 6). It is worth noting that these values are the longest lifetimes reported for Re(I) diimine carbonyl complexes.^28^ Concerning the extreme sensitivity of triplet excited states of **1** and **2** toward oxygen, it is highly probable that their lifetimes are even longer.

**Table 1:** Summary of Emission Properties of **1**–**4**

complex	medium	λ_exc_ (nm)	λ_em_ (nm)	lifetime	χ^2^	QY (%)
**1**	DMSO	375	460	τ_1_ = 1.77 ns	1.054	0.52
		435	657, 720, 790	τ_1_ = 1800.90 μs	1.039	
	77 K^a^	375	405, 430, 450, 494	τ_avr_^b^ = 9.49 ns	0.995	
		370	631, 691, 762	τ_1_ = 169.67 ms	1.005	
**2**	DMSO	375	450	τ_avr_^b^ = 2.12 ns	0.995	1.06
		420	636	τ_1_ = 1513.84 μs	1.016	
	77 K^a^	420	608, 663, 730	τ_1_ = 372.38 ms	1.078	
**3**	DMSO	405	625	τ_1_ = 223.27 ns	0.993	3.11
	77 K^a^	400	546	τ_avr_^b^ = 7.76 μs	0.999	
**4**	DMSO	405	634	τ_1_ = 179.47 ns	0.956	3.82
	77 K^a^	400	549	τ_avr_^b^ = 11.85 μs	0.973	

a Ethanol:methanol (4:1 v/v) glassy matrix at 77 K.

b Average emission lifetime. For more details, see Figure S24 in the Supporting Information.

To gain further insight, photoluminescence properties of the title complexes were investigated at low temperature (77 K). These studies also revealed noticeable differences between **1** and **2**. While complex **2** presents only a vibronically structured emission band attributed to the ^3^π_pyrene_, the frozen-state emission spectrum of **1** includes both ligand-based fluorescence (^1^IL) and phosphorescence (^3^π_pyrene_) components. The bathochromic shift of the phosphorescence for complex **1** in comparison to **2** indicates significantly larger participation of ^3^ILCT (^3^π_pyrene_ → ^3^π*(imphen)) in the case of **1**.^70^ The triplet excited-state lifetimes of pyrenyl-substituted Re(I) complexes (∼170 ms for **1** and ∼370 ms **2**) are shorter than that for free pyrene (700 ms)^71,72^ but a few orders of magnitude longer than those for parent chromophores at 77 K (average on 7.76 μs for **3** and 11.85 μs for **4**).

### ^3^MLCT–^3^IL_pyrene_ Energy Gap

Extended triplet emission lifetimes and reduced photoluminescence quantum yields of pyrenyl-substituted Re(I) complexes at RT in relation to reference chromophores are supportive of the energetic proximity of metal-to-ligand charge transfer and pyrene-localized triplet excited states.

**Figure 7:**
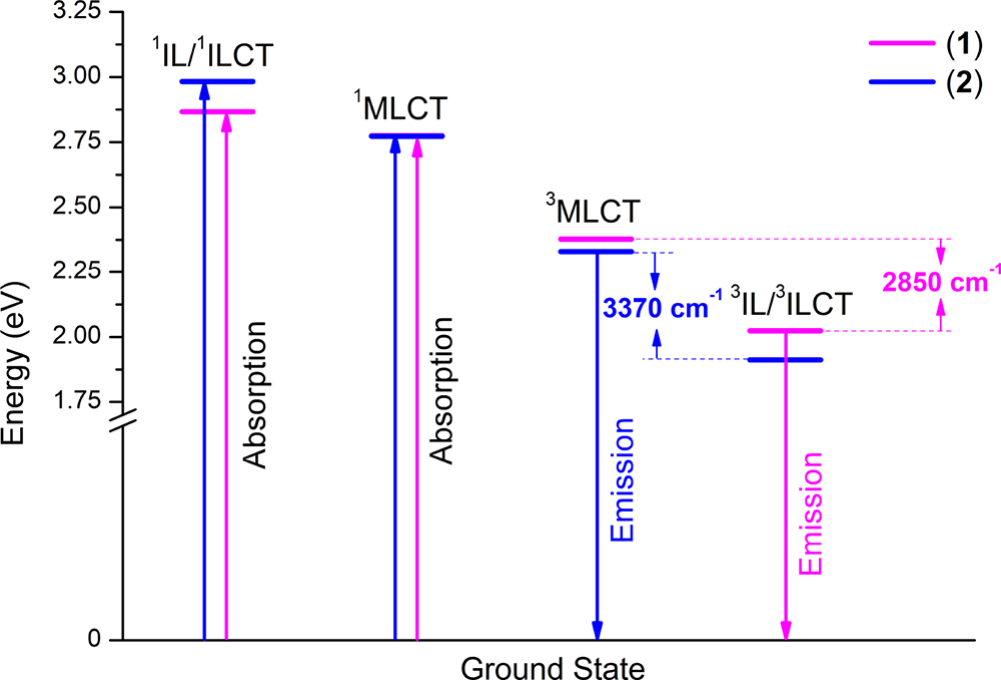
Calculated energies of singlet and triplet excited states of **1** and **2** and the ^3^MLCT–^3^IL energy gaps. The energies of the ^1^MLCT and ^1^IL^/1^ILCT excited states were obtained by the tangent line on the lowest-energy side of the longest wavelength absorption band of the appropriate model chromophores (**3** and **4**) and free ligands (L1 and L2), respectively.

To estimate the energy gap between the ^3^MLCT and ^3^IL_pyrene_ triplet excited states in the pyrene-substituted Re(I) carbonyls (**1** and **2**), two different methodologies were used. In the first approach, the energies of excited states ^3^MLCT were determined from the room-temperature emission band of the appropriate model chromophore (**3** and **4**) by taking the tangent line on the high-energy side of the emission band and its intersection with the wavelength axis. The triplet energies ^3^IL_pyrene_ were estimated by drawing the tangent line on the high-energy side of the frozen-state phosphorescence band of the corresponding free ligands (L1 and L2) and its intersection with the wavelength axis (Figure S28). The triplet emissions of the free ligands were sensitized by the addition of 10% ethyl iodide. The difference of obtained energy values of ^3^MLCT and ^3^IL_pyrene_ states gives energy gaps of 2850 cm^–1^ for **1** and 3372 cm^–1^ for **2** (Figure 7).

In the second method, the triplet energy gaps between ^3^MLCT and ^3^IL_pyrene_ excited states of **1** and **2** were calculated using the following eqs 2–5:
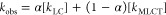
2
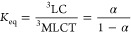
3
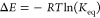
4
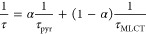
5where τ, τ_pyr_, and τ_MLCT_ are the phosphorescence lifetimes of pyrene-substituted Re(I) carbonyl (**1** or **2**), pure pyrene (700 ms), and corresponding model complex, respectively. The parameter α represents the fraction of the ^3^IL_pyrene_ excited state, while 1 – α corresponds to the fraction of the ^3^IL_pyrene_ excited state in the established equilibrium. Also, in this method, the calculated energy gap between the ^3^MLCT and ^3^IL_pyrene_ triplet is larger for complex **2**, but the obtained values are expectedly smaller, 1845 cm^–1^ for **1** and 1854 cm^–1^ for **2**.

As shown in Figure 7, a weaker electronic communication between the metal-to-ligand charge transfer and pyrene localized triplet excited states, achieved by the increase in the dihedral angle between imphen and pyrenyl moieties due to the introduction of the 4-(methyl)phenyl substituent into the imidazole ring at the H1-position, leads to stabilization of both ^3^MLCT and ^3^IL_pyrene_, simultaneously resulting in the increase in the energy gap between them.

### Femtosecond Transient Absorption

**Figure 8:**
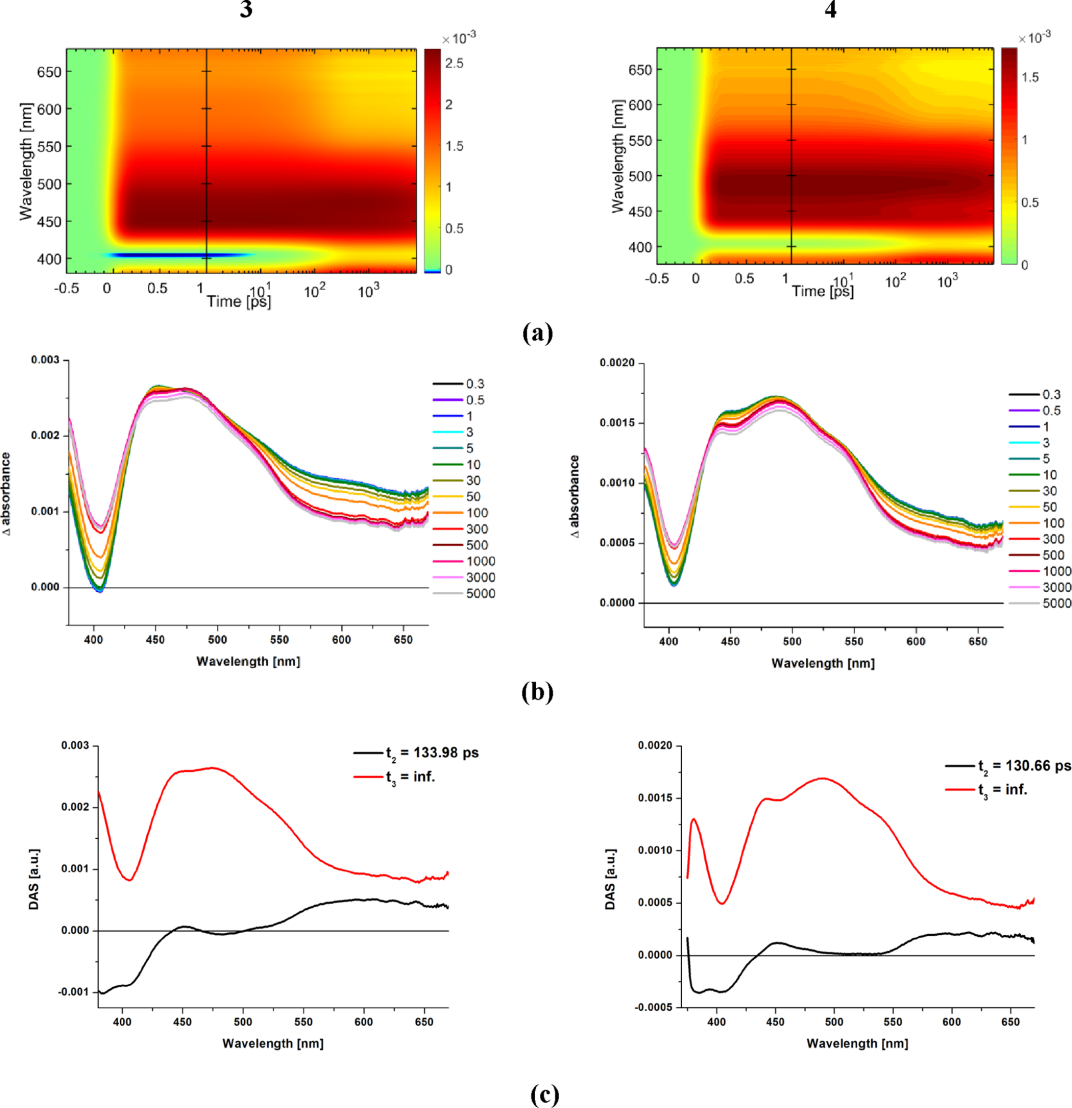
Summary of fs-TA studies for model complexes **3** and **4** at the 355 nm pump: (a) 2D time–wavelength plots; (b) fs-TA spectra at selected time delays; (c) decay-associated spectra (DAS; see also complete data in Figure S31).

**Figure 9:**
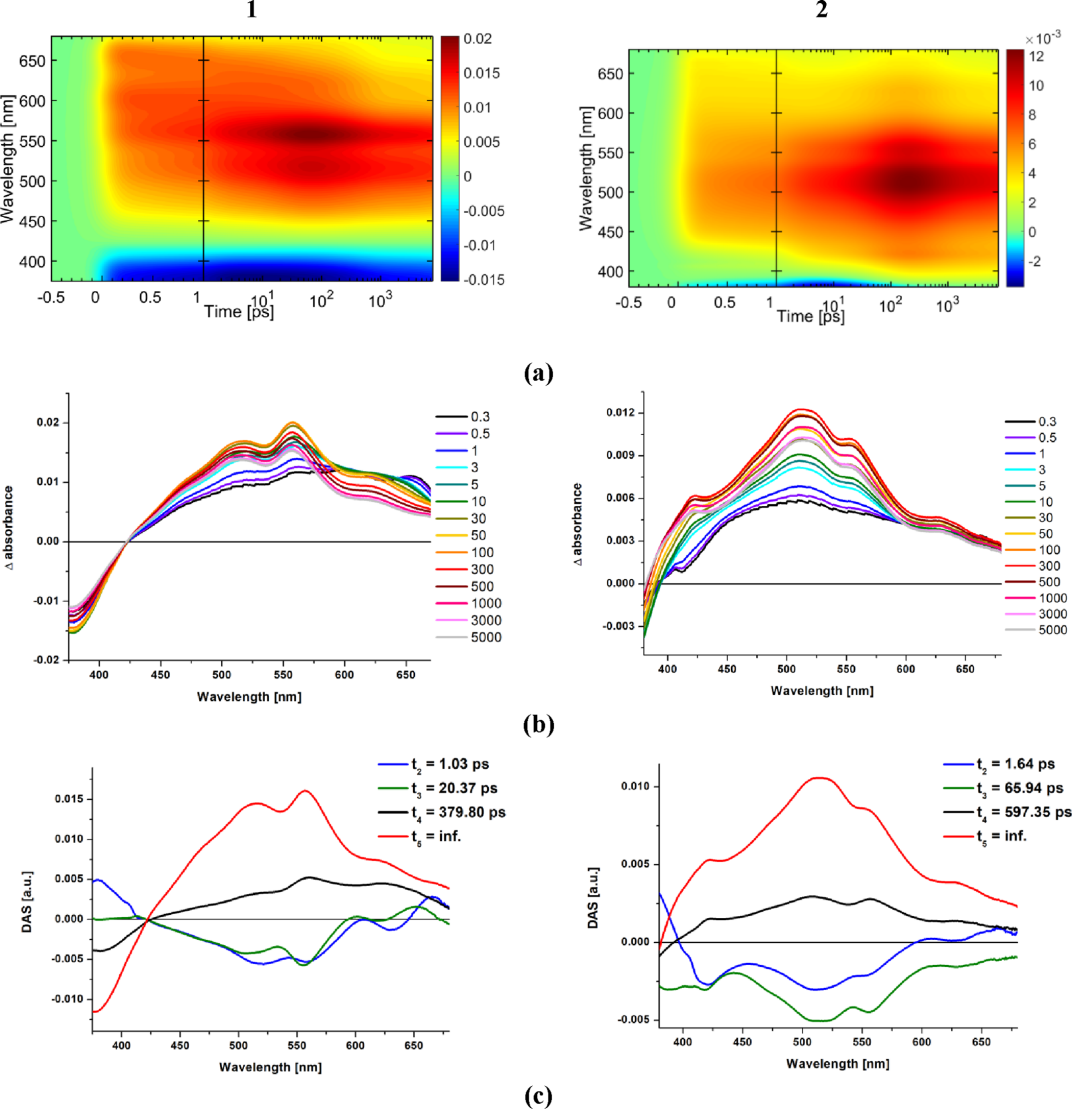
Summary of fs-TA studies for complexes **1** and **2** at the 355 nm pump. (a) 2D time–wavelength plots; (b) fs-TA spectra at selected time delays; (c) decay-associated spectra (DAS; see also complete data in Figures S29 and S30).

To get further insight into photophysical processes and examine their excited-state dynamics, femtosecond transient absorption (fs-TA) studies were performed for **1**–**4** in DMSO, and obtained fs-TA data were analyzed by global analysis. For reference complexes, fs-TA experiments were performed with the excitation at 355 nm, while pyrenyl-substituted Re(I) carbonyls were excited at the blue and red edges of the lowest energy absorption, which are at 355 and 420 nm. For complexes **1** and **2**, two different pump wavelengths were used because the lowest energy absorption band of these systems was found to be contributed by both ^1^pyrene and ^1^MLCT excited states, and both Re(I) carbonyls displayed excitation-dependent dual fluorescence–phosphorescence emission. The results of fs-TA studies are summarized in Figures 8 and 9 and Figures S29–S32.

The fs-TA spectra of model complexes are dominated by broad intense excited-state absorption (ESA) in the range of 420–590 nm (Figure 8 and Figure S31), present up to the end of the delay stage (7 ns). This spectral profile is attributable to the ^3^MLCT excited state, populated due to ^1^MLCT → ^3^MLCT transition.^73,53^ The intersystem crossing process (t_1_) occurs in the time range shorter than the instrument response, while the lifetimes t_2_ and t_3_ determined from global fit analysis (Figure 8) can be assigned to vibrational/structural relaxation of the lowest triplet state ^3^MLCT and ground-state recovery, respectively.

Following photoexcitation of **2** at 355 nm (Figure 9), an instant spectrally unstructured ESA band in the visible region is observed. With reference to the model complex **4**, it can be assigned to the hot ^3^MLCT excited state. Within 390–675 ps, this component undergoes vibrational cooling, which is manifested by a blue-shift of the signal. With increasing delay time, the ESA evolves into the band with three discernible maxima at 420, 516, and 556 nm. To a small extent, the ESA spectral shape may be slightly distorted at 400–520 nm due to the possible overlapping with the stimulated fluorescence occurring as a result of incomplete energy transfer from ^1^IL to ^1^MLCT (Figure S30). Undoubtedly, however, the TA spectral features of **2** are supportive of the ^3^MLCT–^3^IL_pyrene_ equilibrium. The intense absorption with maxima at 516 and 556 nm is attributable to the ^3^IL_pyrene_ excited state, while the higher energy maximum at 420 nm represents ^3^MLCT.^72,74,75^ The triplet excited state on the pyrene chromophore is populated due to the triplet–triplet energy transfer (TTET) from the initially formed ^3^MLCT state. Both ^3^MLCT and ^3^IL_pyrene_ excited states are present up to the end of the delay stage. Regarding the spectral features, the photophysical processes occurring upon photoexcitation of **2** may be represented by the following scheme ^1^MLCT → ^3^MLCT ↔ ^3^IL_pyrene_, followed by the ground-state recovery.

With the use of the global analysis, the fs-data of **2** were the best fit with four decay associated species (DAS_i_) characterized by time constants *t*_*i*_. The ultrafast intersystem crossing ^1^MLCT → ^3^MLCT (*t*_1_) was not resolved. It occurs in a time range shorter than the instrument response. The component with time constant of 1.64 ps (DAS_2_) was assigned to the vibrational cooling of the ^3^MLCT excited state. The decay-associated species with a time constant of 65.94 ps (DAS_3_) represents the formation of the ^3^IL_pyrene_ excited state, which is followed by vibrational/structural relaxation of this state (DAS_4_ with *t*_4_ = 597.35 ps). With reference to previous reports,^72,74,75^ structural changes in the molecular geometry of **2** in the electronically triplet excited state mainly concern the pyrene-imphen torsion angle. Its decrease in the triplet excited state facilitates the population of the ^3^IL_pyrene_ excited state via the TTET mechanism.

For complex **1** excited at 355 nm, the spectral features at longer time delays are also indicative of the presence of both ^3^MLCT and ^3^IL_pyrene_. In relation to **2**, however, the higher energy shoulder corresponding to the ^3^MLCT excited state shows a noticeable red-shift, and is largely overlapped with components which represent the T_1_ → T_*n*_ transitions of the pyrene unit (maxima at 515 and 558 nm). A bathochromic shift is also visible for partly observed ground-state bleaching (GSB) of **1** in relation to **2**, which correlates well with UV–vis results. The stimulated fluorescence may slightly distort the ESA spectral shape in the range of 405–550 nm (Figures S29 and S30).

The most striking difference between **1** and **2** is noticeable in the region >600 nm, where complex **1** shows a more intense absorption contrary to **2**. With reference to TA spectra of the free ligands (Figure S32), this component most likely represents the population of the ILCT (pyrene → imphen) excited state. Therefore, taking into consideration the temporal evolution of transient absorption spectra and spectral profiles of decay-associated species of **1**, it can be assumed that the more planar geometry of pyr-imphen facilitates the population of the ^3^IL_pyrene_ excited state and leads to the formation of ^3^IL_pyrene_ via two paths ^1^MLCT → ^3^MLCT → ^3^IL_pyrene_ and ^1^ILCT → ^3^IL_pyrene_/^3^ILCT. The lifetime *t*_2_ is attributed to the formation of the ^3^IL_pyrene_ excited state via channel ^1^MLCT → ^3^MLCT → ^3^IL_pyrene_, while ^1^ILCT → ^3^IL_pyrene_/^3^ILCT is represented by DAS_3_ (*t*_2_). In analogy to **2** and parent complexes, ISC (^1^MLCT → ^3^MLCT) occurs in the time range shorter than the instrument response. The decay-associated species with a time constant of 379.8 ps (DAS_4_) represents the vibrational/structural relaxation of ^3^IL_pyrene_, while the component with infinite lifetime (DAS_5_) corresponds to the ground-state recovery.

Upon excitation at 420 nm, complexes **1** and **2** show fs-TA spectra, which are qualitatively similar to those for photoexcitation at 355 nm, and only negligible differences can be noticed in estimated time constants *t_i_*. The complete fs-TA data for pyrenyl-substituted Re(I) complexes are demonstrated in Figures S29 and S30.

### Singlet Oxygen Generation

**Figure 10:**
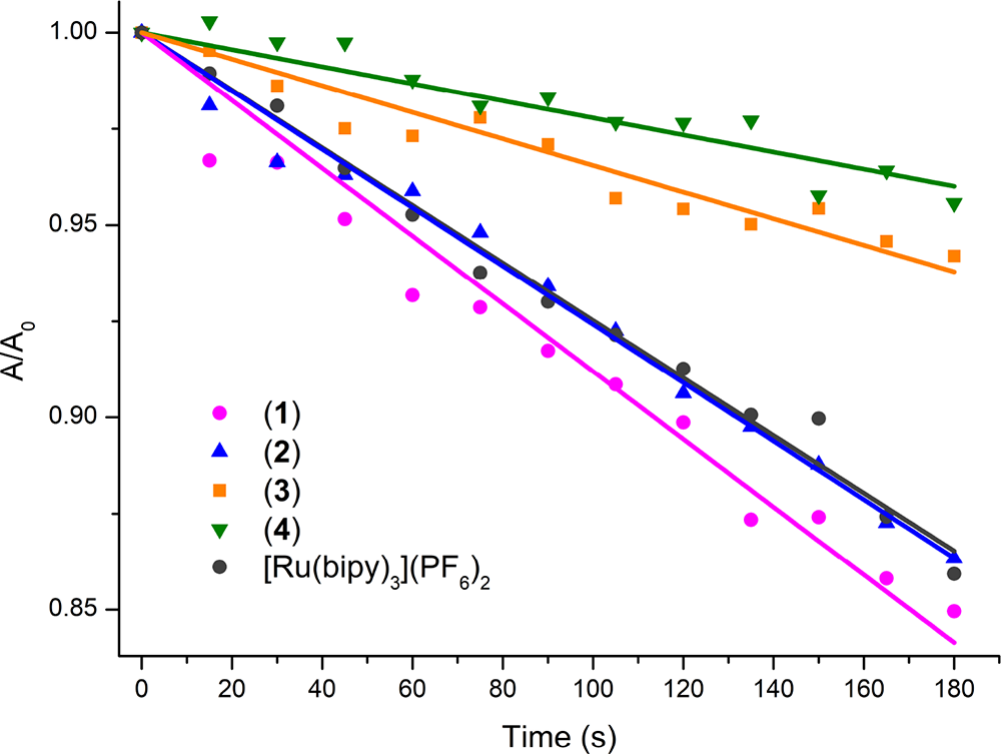
Relative changes in the absorbance of DPBF at 417 nm (*A*/*A*_0_) against the irradiation time in the presence of the studied metal complex, where *A*_0_ stands for absorbance at *t* = 0 s, and *A* stands for absorbance after consecutive irradiation times.

The efficiency of Re(I) complexes in the generation of singlet oxygen was evaluated by an indirect method using [Ru(bipy)_3_](PF_6_)_2_ as the reference standard and 1,3-diphenylisobenzofuran (DPBF) as the singlet oxygen scavenger. The latter one is highly reactive toward ^1^O_2_, forming endoperoxide, which spontaneously decomposes to 1,2-dibenzoylbenzene.^76−79^ The progress of photo-oxidation of DPBF in the presence of Re(I) complexes and [Ru(bipy)_3_](PF_6_)_2_ was monitored by the gradual decrease in the absorption of DPBF at 417 nm (Figure S33) and quantitatively compared by plotting *A*/*A*_0_ against the irradiation time (Figure 10).

The obtained ^1^O_2_ quantum yield Φ_Δ_ values for these complexes follow the order **1** (82.6%) > **2** (68.8.1%) > **3** (30.7%) > **4** (18.9%), which is consistent with the trend observed for triplet excited-state lifetimes and visible absorptivity for these systems. Both pyrenyl-substituted Re(I) complexes (**1** and **2**) show remarkably enhanced singlet oxygen-sensitizing abilities compared to model chromophores (**3** and **4**). With the singlet oxygen quantum yield of ∼80%, complex **1** belongs to a family of the most efficient ^1^O_2_ photosensitizers.^80^

### Triplet–Triplet Annihilation Upconversion (TTA UC)

**Figure 11:**
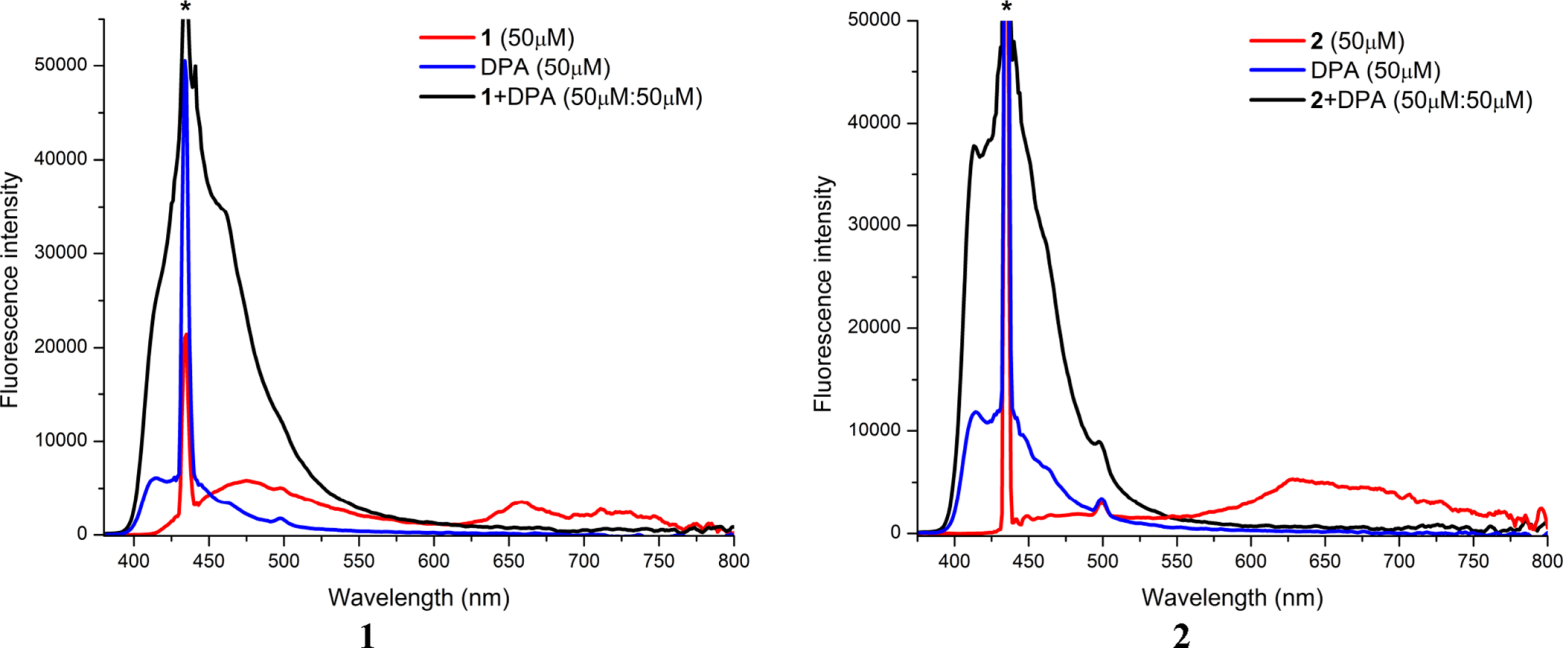
Emission spectra displaying TTA upconversion of 9,10-diphenylanthracene (DPA) in the presence of complexes **1** and **2**. The asterisk denotes an excitation wavelength of 435 nm.

The TTA UC studies were performed upon photoexcitation at 435 nm, with the use of 1,10-diphenylanthracene (DPA) as the triplet acceptor (Figure 11 and Figures S34 and S35). The energy of the lowest triplet excited state of DPA is 1.77 eV. In the presence of DPA, due to the triplet–triplet energy transfer (TTET) between the photosensitizer and the annihilator, the phosphorescence of pyrenyl-substituted complexes was quenched, and lifetimes of the unconverted emission were found to be extremely longer (146 μs for **1** and 172 μs for **2**) than the prompt fluorescence lifetime of DPA (6.50 ns), supporting the delayed fluorescence sensitized by pyrenyl-substituted complexes (Figure S35). For reference complexes, in agreement with their short phosphorescence lifetimes, no upconverted emission was observed (Figure S34).

To estimate the efficiency of TTET processes in pyrenyl-substituted complexes, the Stern–Volmer photoluminescence intensity quenching experiments were performed for **1** and **2** with DPA in deaerated DMSO. The obtained quenching constants (*K*_SV_), 1.12 × 10^7^ for **1** and 9.88 × 10^5^ M^–1^ for **2**, support a higher efficiency of TTEF for **1**, in agreement with its stronger visible absorptivity and more extended triplet excited-state lifetime in relation to complex **2**.

## Conclusions

Using the bichromophore strategy, two long-lived Re(I)-based triplet emitters able to perform triplet–triplet energy transfer were obtained and thoroughly explored with the aid of static and time-resolved spectroscopic methods, including transient absorption. We demonstrated that coordination of 2-(1-pyrenyl)-1*H*-imidazo[4,5-*f*][1,10]phenanthroline (pyr-imphen) and 1-(4-(methyl)phenyl)-2(1-pyrenyl)-imidazo[4,5-*f*][1,10]phenanthroline (pyr-tol-imphen) to {ReCl(CO)_3_} resulted in formation of Re(I) tricarbonyl complexes with record-high triplet excited-state lifetimes in solution at room temperature. Additionally, we evidenced the possibility of tuning photoinduced processes and triplet–triplet energy processes in the bichromophore metal complexes with imidazophenanthroline-based ligands by the steric hindrance of substituents introduced into the imidazole ring at the H1-position. On the basis of steady-state and time-resolved spectroscopic data, the differences in the photophysical behavior of **1** and **2** were rationalized by the contribution of intraligand charge-transfer (ILCT) transitions originating from charge delocalization from the pyrenyl group to the imphen acceptor framework (^1^ILCT and ^3^ILCT), dominant in the case of the complex with stronger electronic coupling between the pyrenyl and imphen moieties (**1**) but almost absent for **2**. Although slightly better functional parameters were achieved for [ReCl(CO)_3_(pyr-imphen)] (**1**), an important advantage of the introduction of the 4-(methyl)phenyl substituent into the imidazole ring at the H1-position was a significant increase in the solubility of the resulting Re(I) complex [ReCl(CO)_3_(pyr-tol-imphen)] (**2**), beneficial regarding potential photochemical applications.

The usefulness of designed compounds as triplet photosensitizers was verified by an examination of their abilities for ^1^O_2_ generation and triplet–triplet annihilation upconversion. The results presented herein provide an in-depth understanding of the impact of the mutual chromophore orientation on photoinduced processes in bichromophore systems. It can be expected that they will be crucial in view of the development of new efficient photosensitizers for modern technologies, especially that imidazophenanthroline-based ligands are known to be excellent versatile ligands to control the photophysical and antiproliferative behavior of metal complexes.

## Experimental Section

The ligands^81−84^ and Re(I) complexes^53^ were prepared employing methods reported previously. The analytical, structural, and spectroscopic data for obtained compounds are included in the Supporting Information.

Elemental analyses were performed with the use of a Vario EL Cube (Elementar) for C, H, and N contents. IR spectra were recorded using a Nicolet iS5 FTIR spectrophotometer (4000–400 cm^–1^) with the use of the KBr pellet method. NMR spectra were registered on a Bruker Avance 500 NMR spectrometer in DMSO-*d*_6_ or CDCl_3_. X-ray diffraction data were collected at room temperature using a Gemini A Ultra diffractometer (Oxford Diffraction) with MoKα radiation (λ = 0.71073 Å). Crystallographic data for **2**–**4** were deposited with the Cambridge Crystallographic Data Center, CCDC 2279356–2279359. Theoretical calculations were performed using the GAUSSIAN-16 program package^85^ at the DFT or TD-DFT level with the PBE0^86,87^ functional, def2-TZVPD basis set for rhenium and def2-TZVP basis set for other elements,^88−90^ and use of the polarizable continuum model (PCM).^91−93^ UV–vis absorption spectra were measured using an Evolution 220 (ThermoScientific) UV–vis spectrometer in DMSO solutions. Emission spectra as well as the time-resolved measurements of argon-saturated samples in DMSO solutions or ethanol:methanol 77 K frozen matrix (4:1 v/v) were measured on a FLS-980 fluorescence spectrophotometer (Edinburgh Instruments). The fs-TA spectra were measured using a pump–probe transient absorption spectroscopy system (Ultrafast Systems, Helios) described previously.^37,70^ Singlet oxygen generation (^1^O_2_) efficiency was determined using the Evolution 220 UV–vis spectrometer in DMSO with 1,3-diphenylisobenzofuran (DPBF) as a sensitizer. Triplet–triplet annihilation upconversion experiments were performed on the FLS-980 fluorescence spectrophotometer with 9,10-diphenylanthracene (DPA) as a triplet acceptor. Detailed synthesis, characterization methods, and extended experimental description are provided in the Supporting Information.

## Supporting Information Available

Additional experimental details, synthetic and experimental data, IR, NMR, single-crystal X-ray analysis, DFT calculations, UV–vis, emission spectroscopy, femtosecond transient absorption, singlet oxygen generation, and triplet–triplet annihilation upconversion data (PDF)
