# Supplementary material for: Pyrenyl-Substituted Imidazo[4,5-f][1,10]phenanthroline Rhenium(I) Complexes with Record-High Triplet Excited-State Lifetimes at Room Temperature: Steric Control of Photoinduced Processes in Bichromophoric Systems
Source: Inorg Chem. 2023 Nov 11;62(47):19256–69. doi: 10.1021/acs.inorgchem.3c02662 (PMC10685448; doi:10.1021/acs.inorgchem.3c02662)
Supplement: Supplementary file 1 — ic3c02662_si_001.pdf [file ic3c02662_si_001.pdf]

SUPPORTING INFORMATION

Pyrenyl-Substituted Imidazo[4,5-f][1,10]phenanthroline Rhenium(I) Complexes with Record-High Triplet Excited-State Lifetimes at Room Temperature: Steric Control of Photoinduced Processes in Bichromophoric Systems

Katarzyna Choroba,* Mateusz Penkala, Joanna Palion-Gazda, Ewa Malicka, Barbara Machura*

Institute of Chemistry, University of Silesia, Szkolna 9, Katowice 40-006, Poland

*Email: katarzyna.choroba@us.edu.pl.
*Email: barbara.machura@us.edu.pl.

**General characterization**	
Photographs and crystal habits of **3a** and **3b**	**Figure S1**
IR spectra of complexes and respective ligands	**Figure S2**
NMR spectra of Re(I) complexes	**Figures S3–S6**
**X-Ray analysis**	
Crystal data and structure refinement	**Table S1**
Selected bond lengths [Å] and angles [°]	**Table S2**
Short intra- and intermolecular hydrogen bonds	**Table S3**
Short π•••π interactions	**Table S4**
X–Y•••Cg(J)(π-ring) interactions	**Table S5**
Inter- and intramolecular short contacts	**Figure S7**
Molecular packing displayed down the b axis	**Figure S8**
**DFT calculations**	
Selected values of bond lengths and dihedral angles in DFT-optimized structures	**Figure S9**
The calculated and experimental values of selected bond lengths and dihedral angles in DFT-optimized structures	**Table S6**
DFT spin density maps of complexes	**Figure S10**
Percentage contribution of selected molecular fragments to the frontier molecular orbitals and the isosurface plots of the orbitals	**Figures S11–S14**
Calculated NTOs of significant low-energy transitions	**Figures S15–S18**
Calculated theoretical parameters of electron transitions	**Tables S7–S10**
**UV-Vis absorption**	
Summary of UV-Vis properties of complexes	**Table S11**
UV-Vis spectra of compounds **1** and **2**, their respective ligands and model complexes as well as pyrene building block	**Figure S19**
UV-Vis spectra of complexes **1**–**4** and their respective ligands	**Figure S20**
UV-Vis stability of complexes **1**–**4** in DMSO	**Figure S21**
UV-Vis photostability of complexes **1**–**4** in DMSO upon irradiation with wavelength 420 nm	**Figure S22**
**Emission spectroscopy**	
Emission spectra recorded upon different excitation wavelengths	**Figure S23**
Summary of luminescence properties of complexes **1**–**4**	**Figure S24**
Comparison of emission intensity of Re(I) complexes with their respective ligands with identical measurement conditions	**Figure S25**
Time-resolved emission spectra of **1** and **2**	**Figures S26–S27**
Method of calculating energies of MLCT and IL states of **1** and **2**	**Figure S28**
**Femtosecond transient absorption**	
Summary of fs-TA measurements for complexes **1**–**4** and ligands **L1**–**L2**	**Figures S29–S32**
**Singlet oxygen generation**	
UV-Vis absorption spectra of DPBF in DMSO treated with the complexes **1–2**	**Figure S33**
**Triplet–triplet annihilation upconversion**	
Emission spectra displaying TTA upconversion of DPA in presence of complexes **3** and **4**	**Figure S34**
The phosphorence quenching of the complexes **1** and **2** with increasing concentration of DPA	**Figure S35**

General Characterization

Preparation of ligands

All chemicals and solvents for synthesis (of reagent grade) and spectroscopic studies (of HPLC or spectroscopic grade) were commercially available and used without further purification.

The ligands pyr-imphen (L1), pyr-tol-imphen (L2), imphen (L3), tol-imphen (L4) were prepared according to the modified method reported in literature[1–4]. Briefly, to a mixture of 1,10-phenanthroline-5,6-dione (5 mmol) and corresponding aldehyde (6 mmol) (formaldehyde or 1-pyrenecarboxaldehyde) a glacial acetic acid (25 ml) and ammonium acetate (91 mmol) were added. In case when *p*-tolyl group was introduced to the nitrogen atom (L1 and L2), also *p*-toluidine (5 mmol) was added to the reaction mixture. After refluxing for 24 h under argon atmosphere, the post-reaction mixture was cooled down to room temperature and poured into 80 ml of water. The obtained suspension was neutralized with concentrated ammonia until pH-indicator paper showed the solution was slightly basic. The resulting precipitate was filtered and washed thoroughly with water and left to dry overnight. Afterwards, crude product was crystallized from ethanol-chloroform mixture, yielding the desired ligand.

L1: ^{1}**H NMR** (400 MHz, DMSO-d_6) δ = 14.12 (s, 1H), 9.54 (d, J = 9.3 Hz, 1H), 9.09-9.07 (m, 3H), 9.02 (d, J = 8.1 Hz, 1H), 8.68 (d, J = 8.0 Hz, 1H), 8.54 (d, J = 8.0 Hz, 1H), 8.45 – 8.36 (m, 3H), 8.36-8.27 (m, 2H), 8.16 (t, J = 7.6 Hz, 1H), 7.93-7.84 (m, 2H) ppm.

L2: ^{1}**H NMR** (500 MHz, $CDCl_3$) δ = 9.23 (dd, J = 4.3, 1.8 Hz, 1H), 9.20 (dd, J = 8.1, 1.8 Hz, 1H), 9.11 (dd, J = 4.3, 1.7 Hz, 1H), 8.27 – 8.18 (m, 3H), 8.14 – 7.99 (m, 5H), 7.93 (d, J = 7.8 Hz, 1H), 7.77 (dd, J = 8.1, 4.4 Hz, 1H), 7.60 (dd, J = 8.3, 1.7 Hz, 1H), 7.35 (dd, J = 8.4, 4.3 Hz, 1H), 7.32 – 7.25 (m, 2H), 7.10 (d, J = 8.0 Hz, 2H), 2.28 (s, 3H) ppm.

L3: ^{1}**H NMR** (500 MHz, DMSO-d_6) δ = 13.72 (s, 1H), 9.06 – 9.00 (m, 2H), 8.83 (bs, 2H), 8.46 (s, 1H), 7.86 – 7.79 (m, 2H). ppm.

L4: ^{1}**H NMR** (500 MHz, DMSO-d_6) δ = 9.08 (dd, J = 4.3, 1.8 Hz, 1H), 9.01 – 8.93 (m, 2H), 8.46 (s, 1H), 7.86 (dd, J = 8.1, 4.3 Hz, 1H), 7.73 (dd, J = 8.3, 1.7 Hz, 1H), 7.67 – 7.60 (m, 2H), 7.57 (dd, J = 8.4, 4.3 Hz, 1H), 7.56 – 7.50 (m, 2H), 2.51 (s, 3H) ppm.

Preparation of Re(I) complexes

Re(I) carbonyl complexes were prepared according to the standard procedure[5] by reacting of $Re(CO)_5Cl$ (0.10 g, 0.27 mmol) and suitable ligand (0.27 mmol) in toluene solution. The resulting mixture was refluxed for 12 h under an argon atmosphere. After this, the resulting precipitate was filtered, dried under air, and then purified by repeated recrystallization from toluene or acetonitrile. X-Ray quality crystals of **2–4** were obtained by the slow evaporation method from acetonitrile solution. In case of compound **3**, two types of crystals were obtained (Figure S1): small yellow block-shaped **3a** and larger yellow plates of **3b**. The crystals were separated manually under microscope. Attempts in changes of synthesis conditions and crystallisation techniques did not result in obtaining a uniform separate sample of any type.

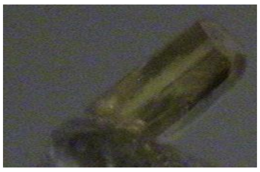	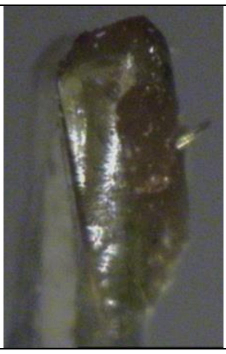
**3a**<br>yellow block<br>0.179 × 0.080 × 0.079 mm	**3b**<br>yellow plate<br>0.387 × 0.155 × 0.026 mm

Figure S1. Photographs and crystal habits of **3a** and **3b**.

1: **Yield**: 0.080 g, 40% **Anal. calc**. for $C_{32}H_{16}N_4ReO_3Cl$ (726.15 g/mol): C 52.93, H 2.22, N 7.72% found: C 52.75; H 2.497; N 7.69%. **IR (KBr, cm^{-1})** intensity: vs - very strong; s - strong, m – medium, w – weak: 3116 (w), 3086 (w) $\nu_{C–H}$; 2025 (vs), 1925 (vs), 1913 (vs) $\nu_{C≡O}$; 1624 (w), 1602 (w), 1552 (w), 1518 (w), 1483 (w), 1455 (w) $\nu_{C=C}$, $\nu_{C=N}$; 847 (m), 821 (m) $\gamma_{C–H}$.

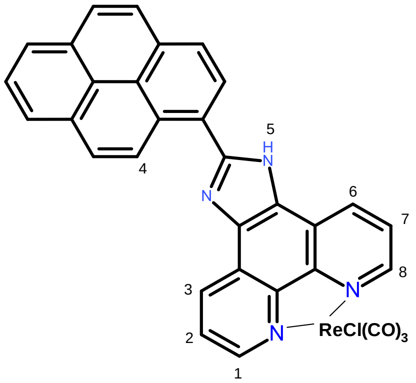

^{1}H NMR (500 MHz, DMSO-d$_6$) δ = 14.63 (s, 1H, H-5), 9.48 (d, *J* = 9.3 Hz, 1H, H-4), 9.44 – 9.41 (m, 3H, H-1, H-8, H-3), 9.36 (d, *J* = 8.3 Hz, 1H, H-6), 8.69 (dd, *J* = 8.0, 1.3 Hz, 1H, H_{pyrene}), 8.57 (dd, *J* = 8.1, 1.7 Hz, 1H, H_{pyrene}), 8.45 – 8.38 (m, 3H, H_{pyrene}), 8.38 – 8.28 (m, 2H, H_{pyrene}), 8.26 – 8.14 (m, 3H) ppm.
^{13}C NMR (126 MHz, DMSO-d$_6$) δ = 197.84, 190.11, 152.95, 151.53, 151.42, 144.00, 143.80, 136.48, 133.06, 132.01, 130.87, 130.28, 128.95, 128.88, 128.83, 128.78, 128.75, 128.19, 127.56, 127.31, 126.97, 126.82, 126.51, 126.18, 125.84, 125.77, 125.22, 124.95, 124.31, 123.61, 121.31 ppm.

2: **Yield**: 0.145 g, 65% **Anal. calc**. for $C_{39}H_{22}N_4ReO_3Cl$ (816.28 g/mol): C 57.38, H 2.72, N 6.86% found: C 57.53; H 2.761; N 6.97%. **IR (KBr, cm^{-1}):** 3045 (w) $\nu_{C–H}$; 2020 (vs), 1907 (vs), 1887 (vs) $\nu_{C≡O}$; 1616 (w), 1602 (w), 1515 (m), 1477 (w), 1452 (m) $\nu_{C=C}$, $\nu_{C=N}$; 851 (m), 807 (w) $\gamma_{C–H}$.

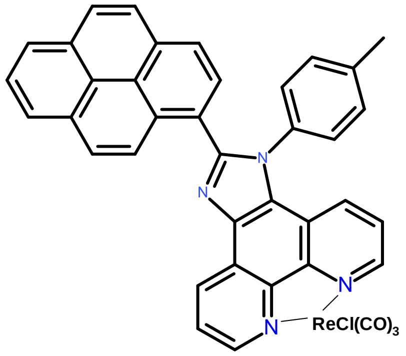

^{1}H NMR (500 MHz, DMSO-d$_6$) δ = 9.47 (dd, *J* = 5.1, 1.4 Hz, 1H), 9.45 (dd, *J* = 8.2, 1.5 Hz, 1H), 9.37 (dd, *J* = 5.1, 1.3 Hz, 1H), 8.39 (d, *J* = 7.7 Hz, 1H), 8.36 (d, *J* = 7.5 Hz, 1H), 8.31 (d, *J* = 8.5 Hz, 2H), 8.26 – 8.17 (m, 5H), 8.14 (t, *J* = 7.6 Hz, 1H), 7.92 (dd, *J* = 8.6, 5.1 Hz, 1H), 7.78 (dd, *J* = 8.5, 1.4 Hz, 1H), 7.67 (dd, *J* = 8.3, 2.3 Hz, 1H), 7.61 (dd, *J* = 8.3, 2.3 Hz, 1H), 7.24 (t, *J* = 6.5 Hz, 2H), 2.23 (s, 3H, CH_3-) ppm.
^{13}C NMR (126 MHz, DMSO-d$_6$) δ = 197.83 (CO), 197.78 (CO), 190.02 (CO), 154.08, 152.12, 151.44, 144.33, 144.01, 140.08, 135.69, 133.50, 133.19, 131.72, 130.64, 130.62, 130.59, 130.54, 130.30, 130.17, 128.76, 128.61, 128.05, 127.99, 127.22, 127.15, 127.02, 126.79, 126.16, 126.11, 125.95, 125.65, 124.79, 124.02, 123.93, 123.37, 123.29, 121.66, 118.08, 20.72 ppm.

3: **Yield**: 0.115 g, 80% **Anal. calc**. for $C_{16}H_8N_4ReO_3Cl$ (525.92 g/mol): C 36.54, H 1.53, N 10.65% found: C 36.37; H 1.726; N 10.46%. **IR (KBr, cm^{-1}):** 3111 (m), 3087 (m), 3050 (w), 2823 (w), 2798 (w), 2773 (w) $\nu_{C–H}$; 2028 (vs), 1952 (vs), 1919 (vs), 1877 (vs) $\nu_{C≡O}$; 1618 (w), 1604 (m), 1535 (w), 1513 (w), 1477 (w) $\nu_{C=C}$, $\nu_{C=N}$; 813 (m) $\gamma_{C–H}$.

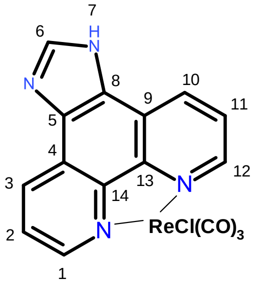

^{1}H NMR (500 MHz, DMSO-d$_6$) δ = 14.25 (s, 1H, H-7), 9.36 (d, *J* = 5.2 Hz, 1H, H-1, H-12), 9.23 – 9.17 (m, 2H, H-3, H-10), 8.73 (d, *J* = 1.3 Hz, 1H, H-6), 8.15 (bs, 2H, H-2, H-11) ppm.
^{13}C NMR (126 MHz, DMSO-d$_6$) δ = 197.83 (CO), 190.04 (CO), 151.33 (C-1/C-12), 143.78 (C-14), 143.08 (C-6), 140.85 (C-13), 135.33 (C-5), 132.71 (C-10), 126.73 (C-2), 126.48 (C-11), 125.89 (C-8), 125.42 (C-4), 121.42 (C-9) ppm.

4: **Yield**: 0.120 g, 70% **Anal. calc**. for $C_{23}H_{14}N_4ReO_3Cl$ (616.04 g/mol): C 44.84, H 2.29, N 9.09% found: C 45.16; H 2.683; N 9.31%. **IR (KBr, cm^{-1}):** 3062 (w), 3028 (w), 2925 (w), 2851 (w) $\nu_{C–H}$; 2021 (vs), 1915 (vs), 1877 (vs) $\nu_{C≡O}$; 1604 (m), 1517 (m), 1480 (w) $\nu_{C=C}$, $\nu_{C=N}$; 858 (w), 820 (m) $\gamma_{C–H}$.

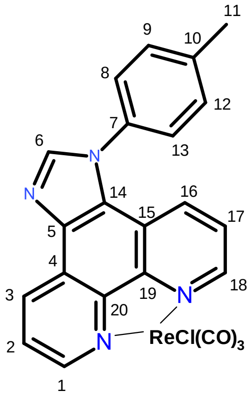

^{1}H NMR (500 MHz, DMSO-d_6) δ = 9.42 (d, *J* = 5.1 Hz, 1H, H-1), 9.34 (d, *J* = 5.3 Hz, 2H, H-18, H-16), 8.77 (s, 1H, H-6), 8.20 (dd, *J* = 7.6, 4.7 Hz, 1H, H-2), 8.03 (d, *J* = 8.6 Hz, 1H, H-3), 7.93 (dd, *J* = 8.6, 5.2 Hz, 1H, H-17), 7.71 (bs, 2H, H-8, H-13), 7.57 (d, *J* = 7.9 Hz, 2H, H-9, H-12), 2.53 (s, 3H, H-11) ppm.
^{13}C NMR (126 MHz, DMSO-d_6) δ = 198.26 (CO), 190.42 (CO), 152.48 (C-1), 151.97 (C-18), 146.81 (C-6), 144.73 (C-20), 144.43 (C-19), 140.79 (C-7), 136.77 (C-5), 133.84 (C-14), 133.43 (C-16), 131.34 (C-8/C-13/C-3), 127.63 (C-2), 127.48 (C-9/C-12), 126.56 (C-17), 126.18 (C-4), 125.81 (C-15), 121.95 (C-10), 21.39 (C-11) ppm.

Crystal structure determination and refinement
For complexes **2–4**, X-ray diffraction data was collected at room temperature using four-circle diffractometer Gemini A Ultra (Oxford Diffraction) with Atlas CCD detector and graphite monochromated MoKα radiation (λ = 0.71073 Å). Diffraction data collection, cell refinement and data reduction were performed using the CrysAlis^{Pro} software[6]. The structures were solved with the direct methods in SHELXS and refined with least square minimization using SHELXL-2014[7]. All the non-hydrogen atoms were refined anisotropically, and hydrogen atoms were placed in calculated positions and refined with riding constrains: d(C–H) = 0.93 Å, U_{iso}(H) = 1.2 U_{eq}(C) (for aromatic) and d(C–H) = 0.96 Å, U_{iso}(H) = 1.5 U_{eq}(C) (for methyl). The methyl groups were allowed to rotate about their local threefold axis. Crystallographic data for **2–4** were deposited with the Cambridge Crystallographic Data Center, CCDC 2279356-2279359. Copies of this information may be obtained free of charge from the Director, CCDC, 12 Union Road, Cambridge CB2 1EZ, UK (Fax: +44 1223 336033; e-mail: deposit@ccdc.cam.ac.uk or www.ccdc.cam.ac.uk).

Computational details
Theoretical calculations (singlet and triplet geometry optimization, molecular orbitals and NTO analysis, calculated absorption spectra) were performed using the GAUSSIAN-16 program package[8] at the DFT or TD-DFT level with the PBE0[9,10] hybrid exchange-correlation functional, and the def2-TZVPD basis set for rhenium and def2-TZVP basis set for other elements[11–13]. The polarizable continuum model (PCM) correction was used to simulate the acetonitrile solvent environment for all calculations[14–16]. After optimization of the geometry, vibrational frequencies were calculated to verify the minimum on the potential energy surface.

Physical measurements
Elemental analyses were performed with use of Vario EL Cube (Elementar) apparatus for N C, H and Cl content.
IR spectra were recorded using Nicolet iS5 FTIR spectrophotometer (4000–400 cm^{-1}) with use of KBr pellets method.
NMR spectra were registered on a Bruker Avance 500 NMR spectrometer in DMSO-d_6 (or in $CDCl_3$ in case of L2). For complexes **3–4,** multidimensional correlation NMR was taken: homonuclear 2D COSY and heteronuclear HMQC and HMBC (long-range) correlation spectra. The full assignment of signals was possible for complexes **3** and **4**, while the significant overlapping of signals for complexes **1** and **2** impeded with the full assignment.
The UV-Vis absorption spectra were measured using ThermoScientific Evolution 220 UV-Vis spectrometer in DMSO solutions. The kinetic stability of each sample was examined by measurement of the series of UV-Vis spectra in 4h intervals for 72h. The photostability of the solutions were performed by measurements of the series of UV-Vis spectra, each after 20 minutes of irradiation of the sample with xenon light source at 420 nm excitation wavelength.

Photoluminescence spectra
The emission spectra of argon-saturated samples in DMSO solutions were measured on FLS-980 fluorescence spectrophotometer (Edinburgh Instruments) equipped with a 450 W Xe arc lamp light source and PMT (Hamamatsu, R928P) detector. Before each measurement of the complexes, freshly prepared solutions were deaerated using 1h of argon-bubbling. The emission spectra at 77 K were measured in an ethanol:methanol (4:1 v/v) matrix frozen with liquid nitrogen. Triplet emission spectra of organic ligands was obtained using 10% ethyl iodide as a phosphorescence sensitizer.

The lifetime measurements were carried out for optically diluted, argon-saturated solutions on FLS-980. Short fluorescence lifetimes were measured with TCSPC method using picosecond EPLED 375 nm pulsed diode as excitation source with 20 μs pulse period, with additional measurement of the IRF for the analysis of a fluorescence decay. Long-lived phosphorescence lifetimes were measured with MCS method with use of 60 W microsecond Xe flash lamp with 10 Hz or 0.1 Hz repetition rate, and the calculations of the lifetimes were performed via FLS-980 software.
Quantum yield of each complex was measured on FLS-980 with the addition of integrating sphere attachment. The measurements were taken using the absolute method for argon-saturated solutions, with DMSO as reference. The emission correction file was applied to take into account the sensitivity of the monochromator, detector, sphere coating and optics to wavelength. The FLS-980 software was used to perform the emission correction and calculation of the quantum yield.

Femtosecond transient absorption

The fs TA spectra were measured using a pump-probe transient absorption spectroscopy system (Ultrafast Systems, Helios) described previously[17,18]. The samples of **1–4,** L1 and L2 were prepared in DMSO, with absorbance of the first absorption band in the range of 0.5–0.7 in the excitation wavelengths (corresponding to concentrations in the range of 1.25×10^{-4} mol/dm^3 for ligands and 10^{-4} – 5×10^{-4} mol/dm^3 for the complexes), and placed in a 2 mm path length quartz cells with magnetic stirring. The samples were excited with 355 nm (for all) and 420 nm pump pulses (for **1** and **2**). Obtained data was prepared using the Surface Xplorer (Ultrafast Systems)[5] with corrections for the probe chirp and solvent signal before each analysis and then analysed with the use of the Optimus™ software[5,19,20]. The software allowed to perform Singular Value Deconvolution of the 3D surface into principal components (spectra and kinetics), Global Analysis (lifetimes, with an error of ~10%), and Decay Associated Spectra, DAS, of the detected transients.

Singlet Oxygen Generation

Singlet oxygen generation ($^1O_2$) efficiency was determined in DMSO using 1,3-diphenylisobenzofuran (DPBF) sensitized by complexes **1–4** that generate singlet oxygen under irradiation with visible light. The reaction between DPBF and generated singlet oxygen results in formation of an endoperoxide, which decomposes to colourless 1,2-dibenzoylbenzene at room temperature. Decrease of the absorbance of DPBF was monitored spectroscopically. The UV-Vis spectra of DPBF and Re(I) complex were recorded in the presence of the complexes exposed to 420 nm wavelength from 15 s to 180 s. Singlet oxygen quantum yields (Φ_Δ) were calculated with use of $[Ru(bipy)_3](PF_6)_2$ as the reference standard ($\Phi_{\Delta O2}$ = 0.66 in DMSO)[21,22] according to equation:

$$\Phi_{\Delta sam} = \Phi_{\Delta ref}\left(\frac{1-10^{-A_{ref}}}{1-10^{-A_{sam}}}\right)\left(\frac{m_{sam}}{m_{ref}}\right)\left(\frac{\eta_{sam}}{\eta_{ref}}\right)^2$$

With "sam" and "ref" representing Re(I) complex sample and reference sample respectively; Φ_Δ is the singlet oxygen quantum yield; A is the absorbance at the irradiation wavelength; m is the slope of the DPBF absorption changing over time and η is the refractive index of the solvent.

Triplet–triplet annihilation upconversion

Triplet–triplet annihilation upconversion experiments were performed on FLS-980 fluorescence spectrophotometer with use of Xe arc lamp as excitation source. 9,10-Diphenylanthracene (DPA) was used as a triplet acceptor. Solutions in DMSO of the DPA, Re(I) complexes **1–4** and the prepared mixtures (1:1 mol/mol) were deaerated before measurements. Samples were photoexcited at 435 nm wavelength, with the uniform excitation and emission slits of the apparatus for each experiment, and the upconverted fluorescence of DPA was observed. The TTET efficiency was measured by the quenching experiments, with changing of the DPA concentration. Fitting of the quenching results with the Stern–Volmer equation $F_0/F = 1 + K_{SV}[Q]$ give the K_{SV} values. F and F_0 are integrated intensities of the Re(I) complexes phosphorescence with and without DPA, K_{SV} is the Stern-Volmer constant, and [Q] is the molar concentration of DPA[23,24].

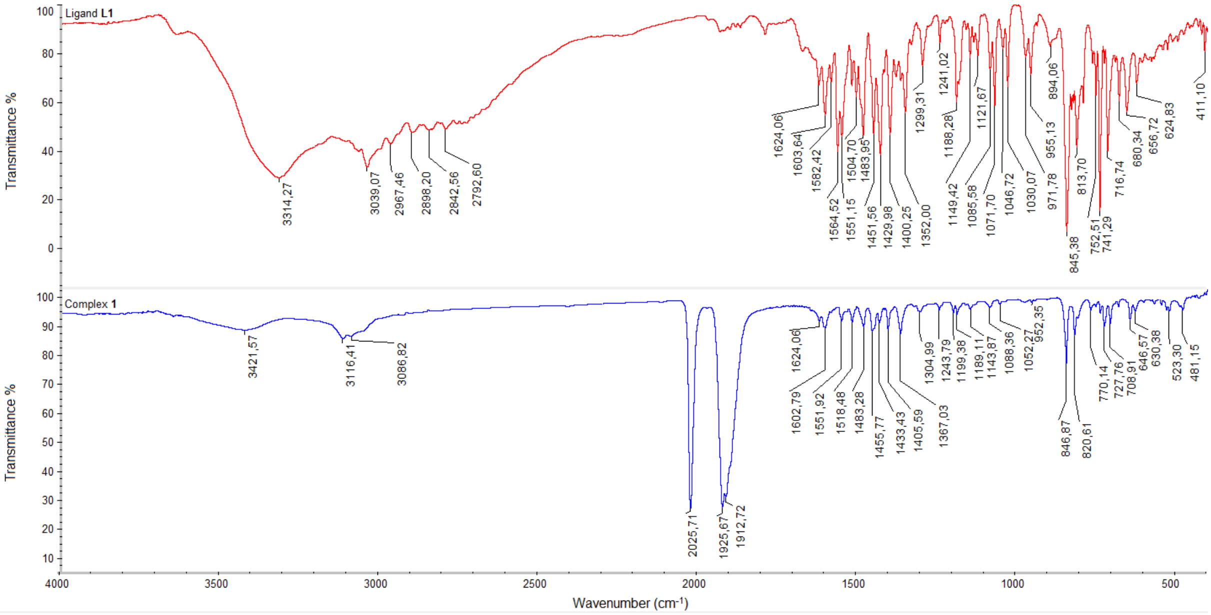
Ligand L1
Complex 1
Transmittance %
Wavenumber (cm-1)
3314,27
3039,07
2967,46
2898,20
2842,56
2792,60
1624,06
1603,64
1582,42
1564,52
1551,15
1504,70
1483,95
1451,56
1429,98
1400,25
1352,00
1299,31
1241,02
1188,28
1149,42
1121,67
1085,58
1071,70
1046,72
1030,07
971,78
955,13
894,06
845,38
813,70
752,51
741,29
716,74
680,34
656,72
624,83
411,10
3421,57
3116,41
3086,82
2025,71
1925,67
1912,72
1624,06
1602,79
1551,92
1518,48
1483,28
1455,77
1433,43
1405,59
1367,03
1304,99
1243,79
1199,38
1189,11
1143,87
1088,36
1052,27
952,35
846,87
820,61
770,14
727,76
708,91
646,57
630,38
523,30
481,15

(a)

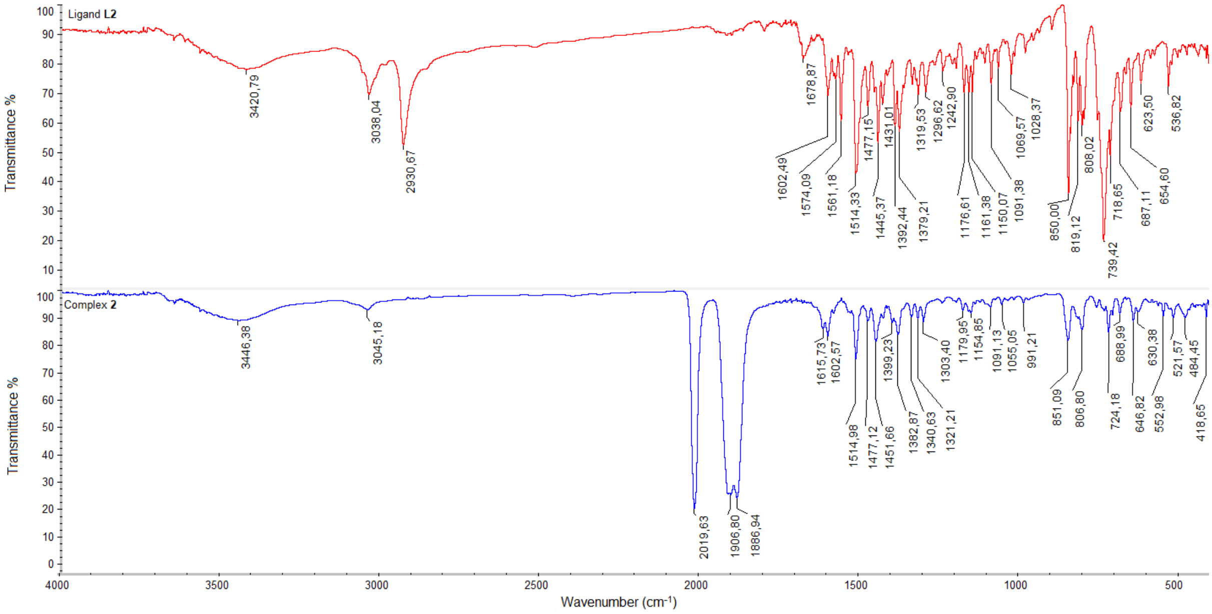
Ligand L2
Complex 2
Transmittance %
Wavenumber (cm-1)
3420,79
3038,04
2930,67
1678,87
1602,49
1574,09
1561,18
1514,33
1477,15
1445,37
1431,01
1392,44
1379,21
1319,53
1296,62
1242,90
1176,61
1161,38
1150,07
1091,38
1069,57
1028,37
850,00
819,12
808,02
739,42
718,65
687,11
654,60
623,50
536,82
3446,38
3045,18
2019,63
1906,80
1886,94
1615,73
1602,57
1514,98
1477,12
1451,66
1399,23
1382,87
1340,63
1321,21
1303,40
1179,95
1154,85
1091,13
1055,05
991,21
851,09
806,80
724,18
688,99
646,82
630,38
552,98
521,57
484,45
418,65

(b)

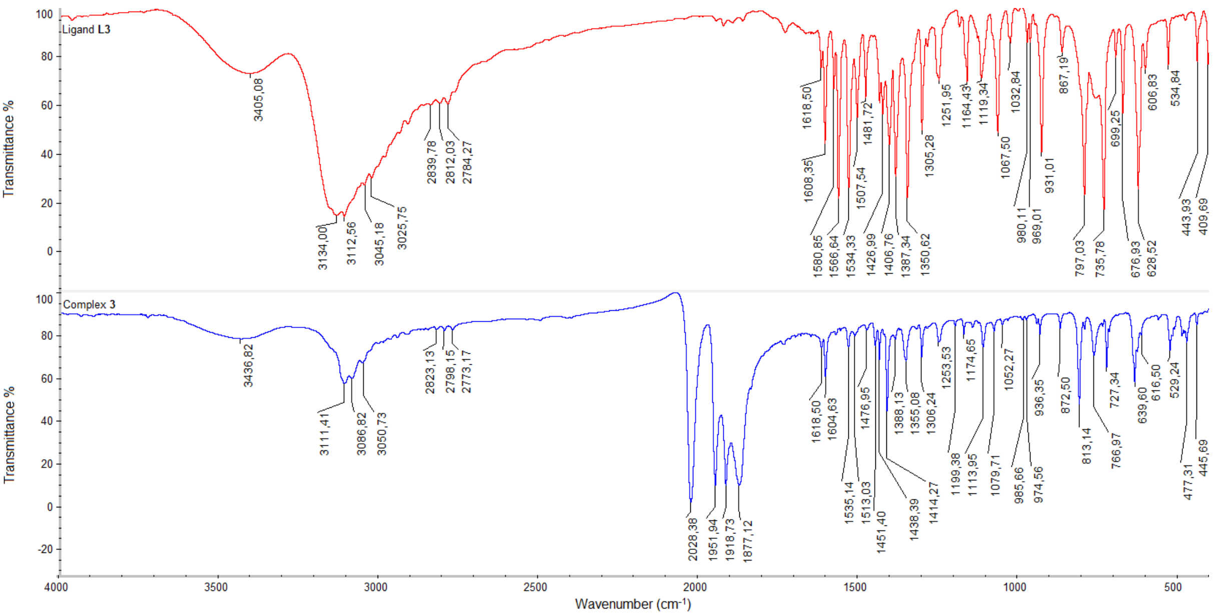

(c)

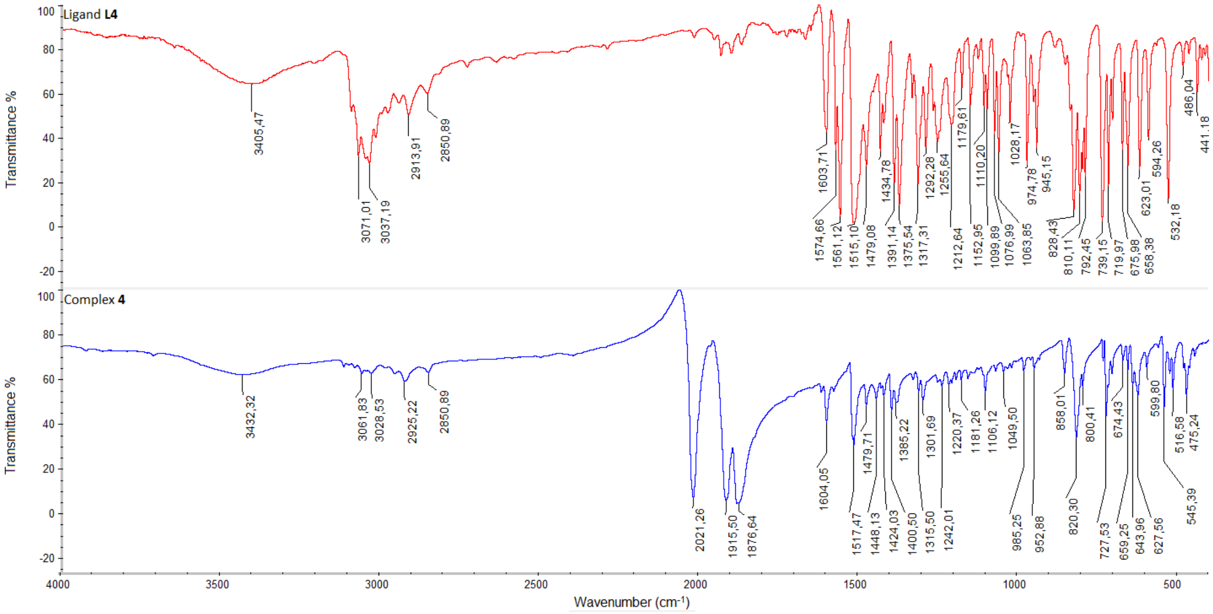

(d)

Figure S2. IR spectra of complexes **1-4** (blue spectra, lower panels) and their respective ligands (red spectra, upper panels) (a–d).

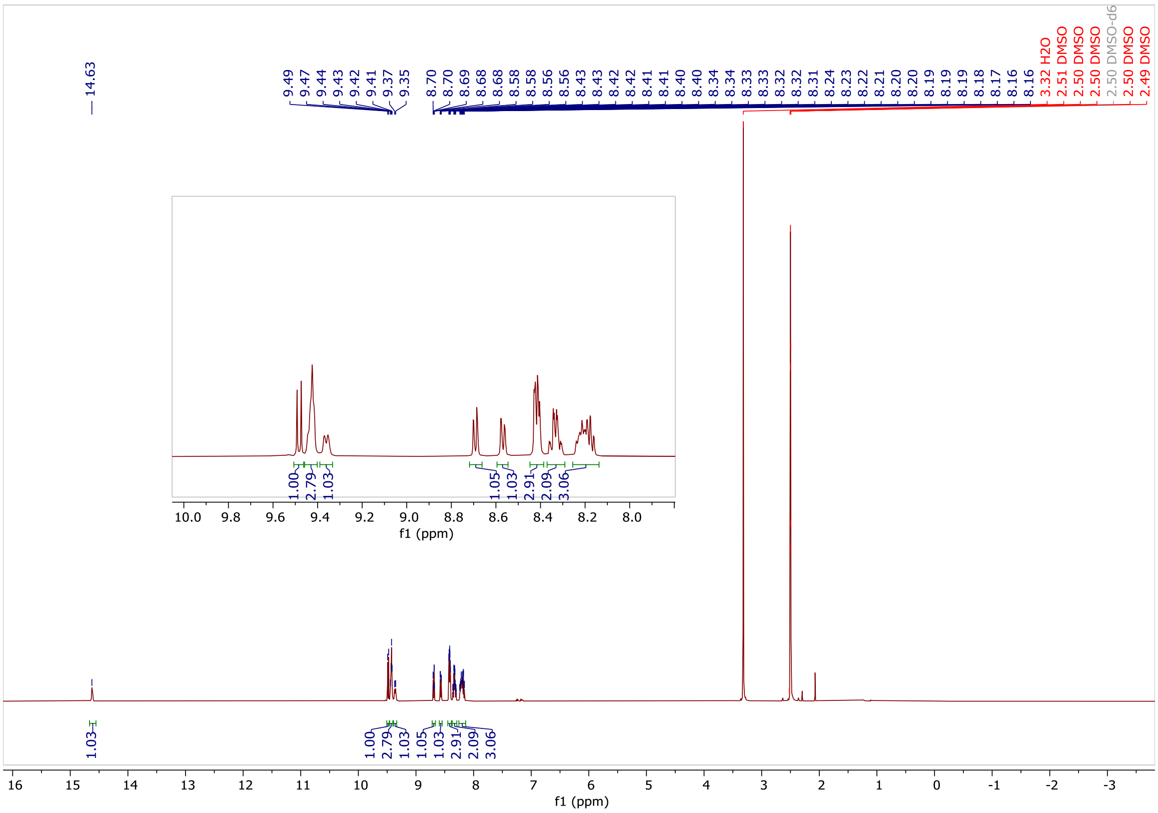

(a)

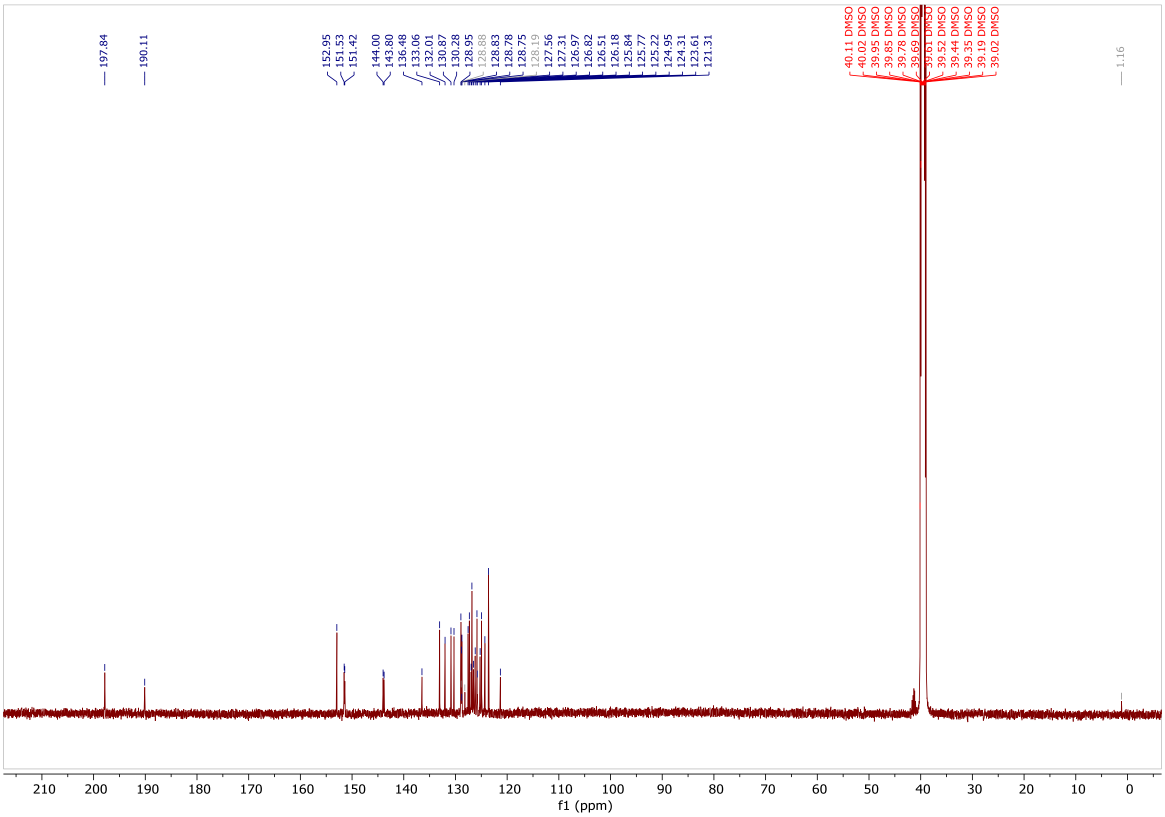

(b)

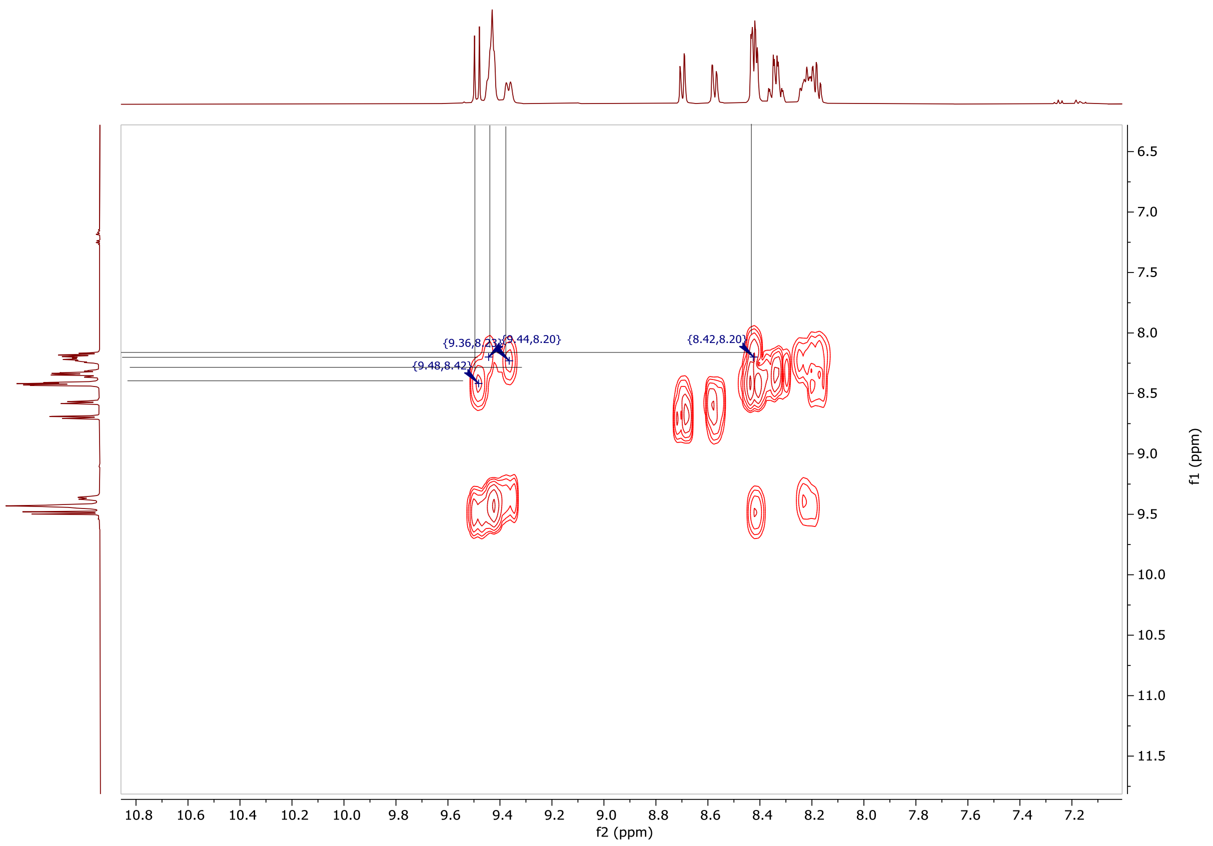
{9.36,8.23}
{9.44,8.20}
{9.48,8.42}
{8.42,8.20}
f2 (ppm)
f1 (ppm)

(c)

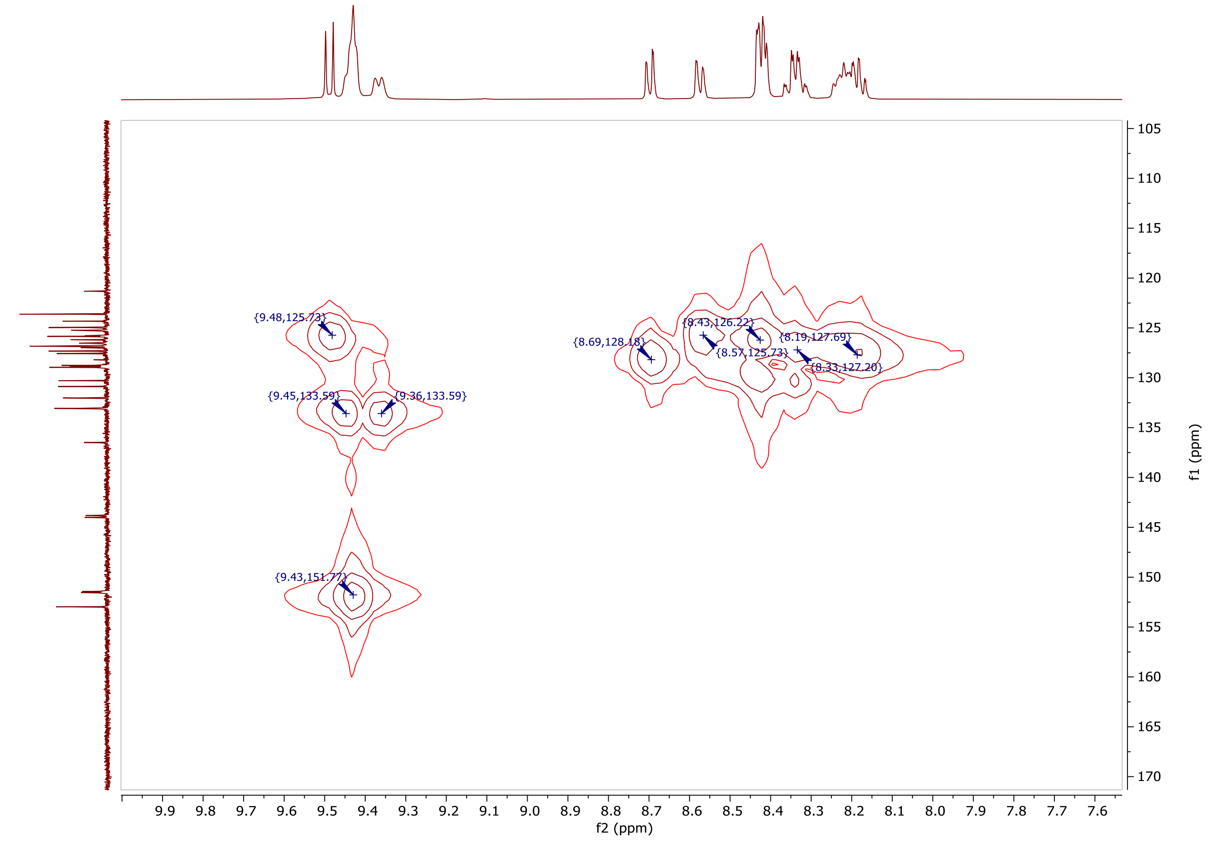
{9.48,125.73}
{9.45,133.59}
{9.36,133.59}
{9.43,151.77}
{8.69,128.18}
{8.43,126.22}
{8.57,125.73}
{8.19,127.69}
{8.33,127.20}
f2 (ppm)
f1 (ppm)

(d)

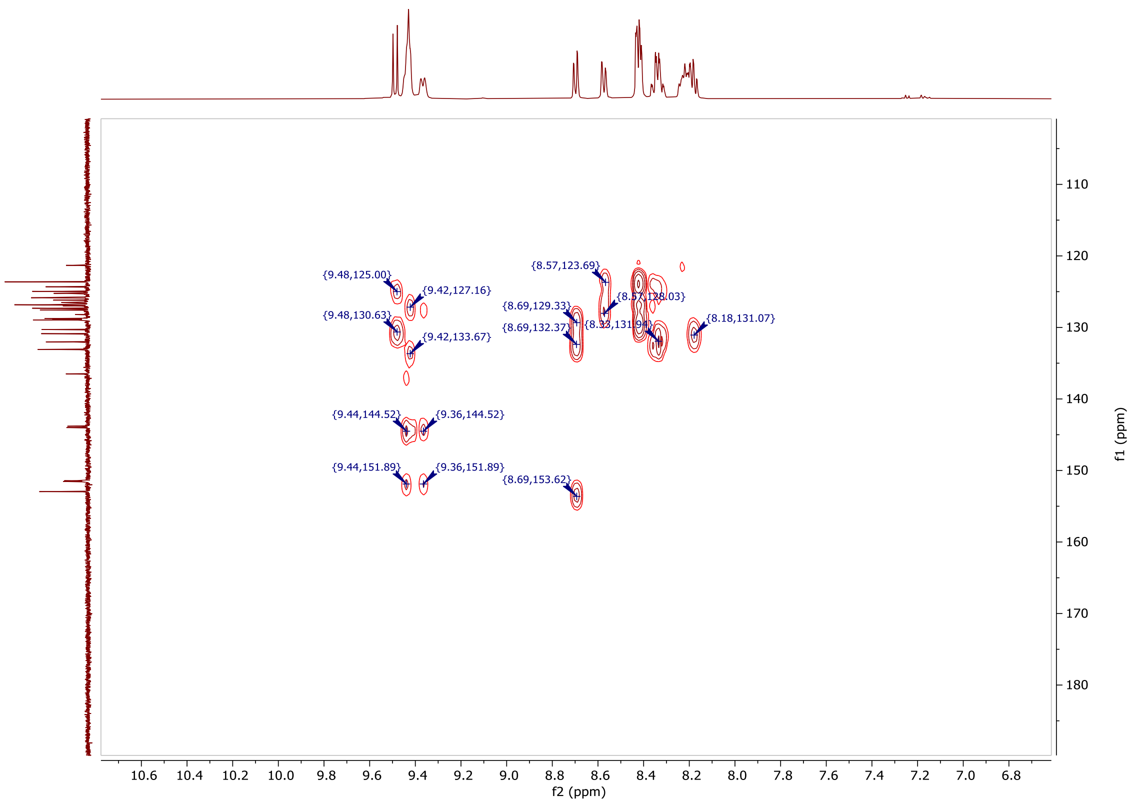

(e)

Figure S3. ^{1}H (a), ^{13}C (b), 2D ^{1}H–^{1}H COSY (c), ^{1}H–^{13}C HMQC (d) and ^{1}H–^{13}C HMBC (e) NMR spectra of complex **1** in DMSO-d$_6$.

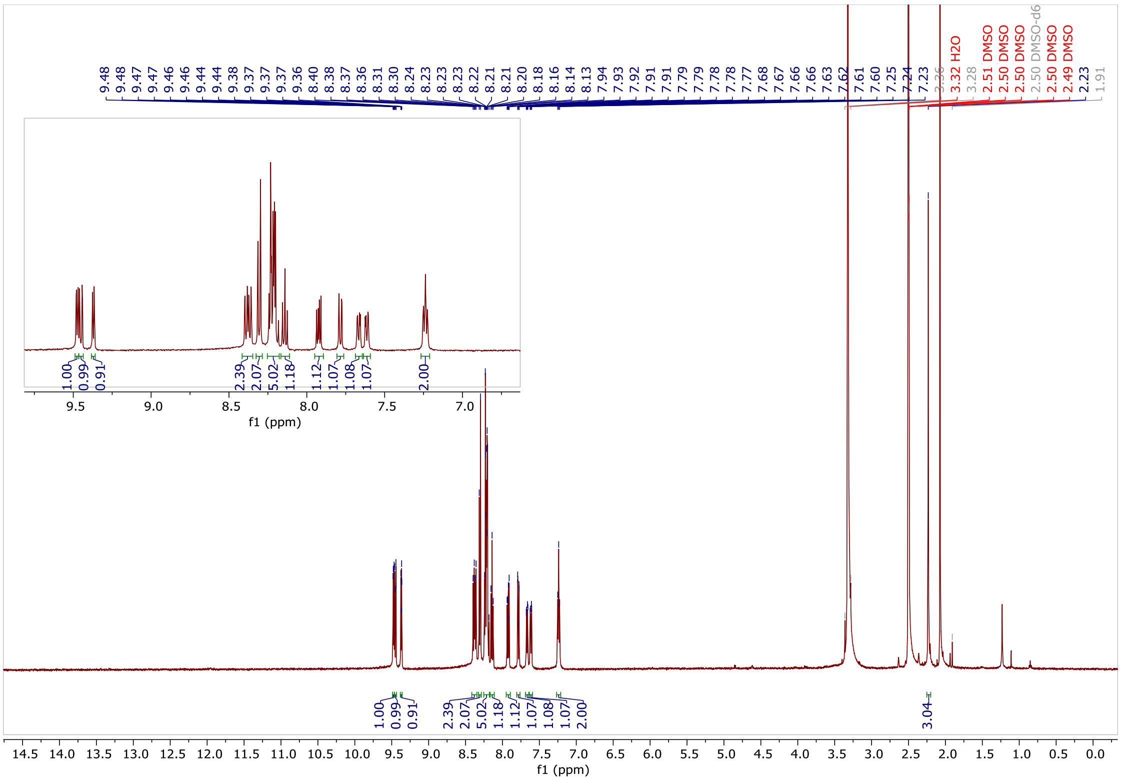

(a)

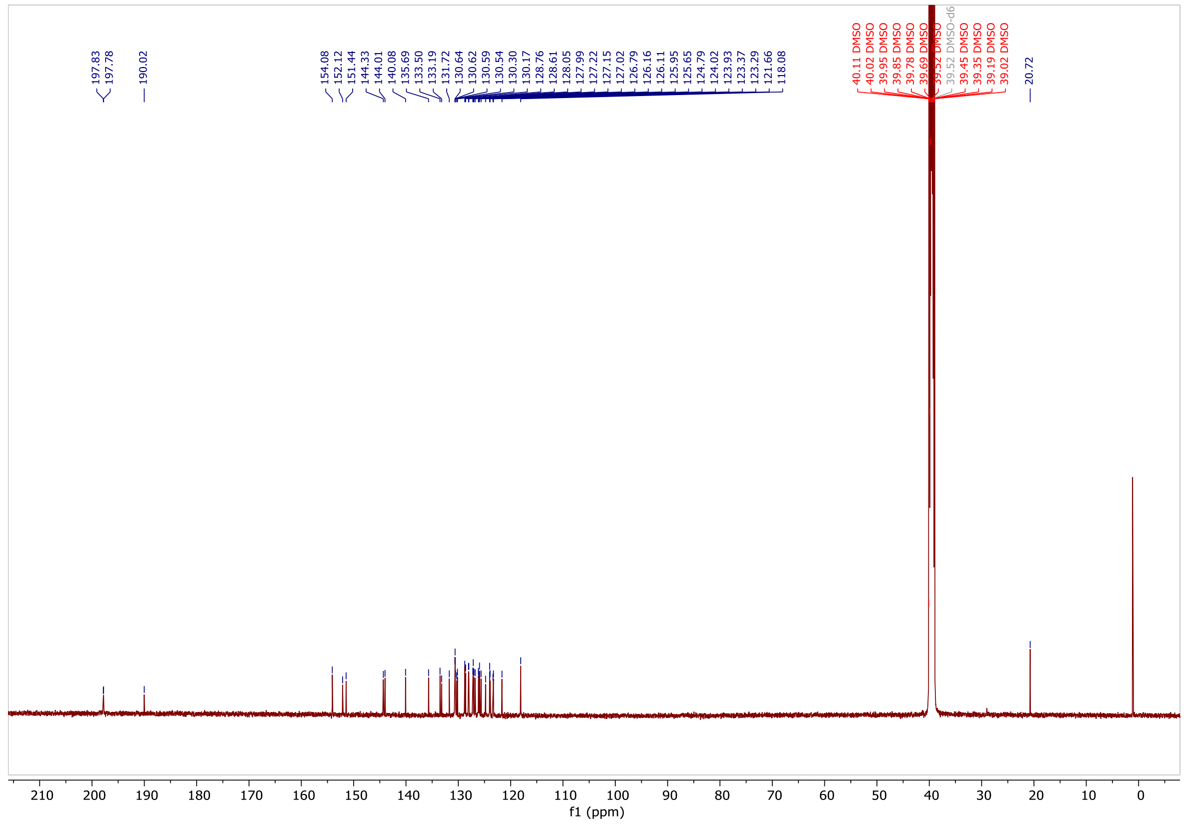
197.83
197.78
190.02
154.08
152.12
151.44
144.33
144.01
140.08
135.69
133.50
133.19
131.72
130.64
130.62
130.59
130.54
130.30
130.17
128.76
128.61
128.05
127.99
127.22
127.15
127.02
126.79
126.16
126.11
125.95
125.65
124.79
124.02
123.93
123.37
123.29
121.66
118.08
40.11 DMSO
40.02 DMSO
39.95 DMSO
39.85 DMSO
39.78 DMSO
39.69 DMSO
39.52 DMSO
39.52 DMSO-d6
39.45 DMSO
39.35 DMSO
39.19 DMSO
39.02 DMSO
20.72
f1 (ppm)

(b)

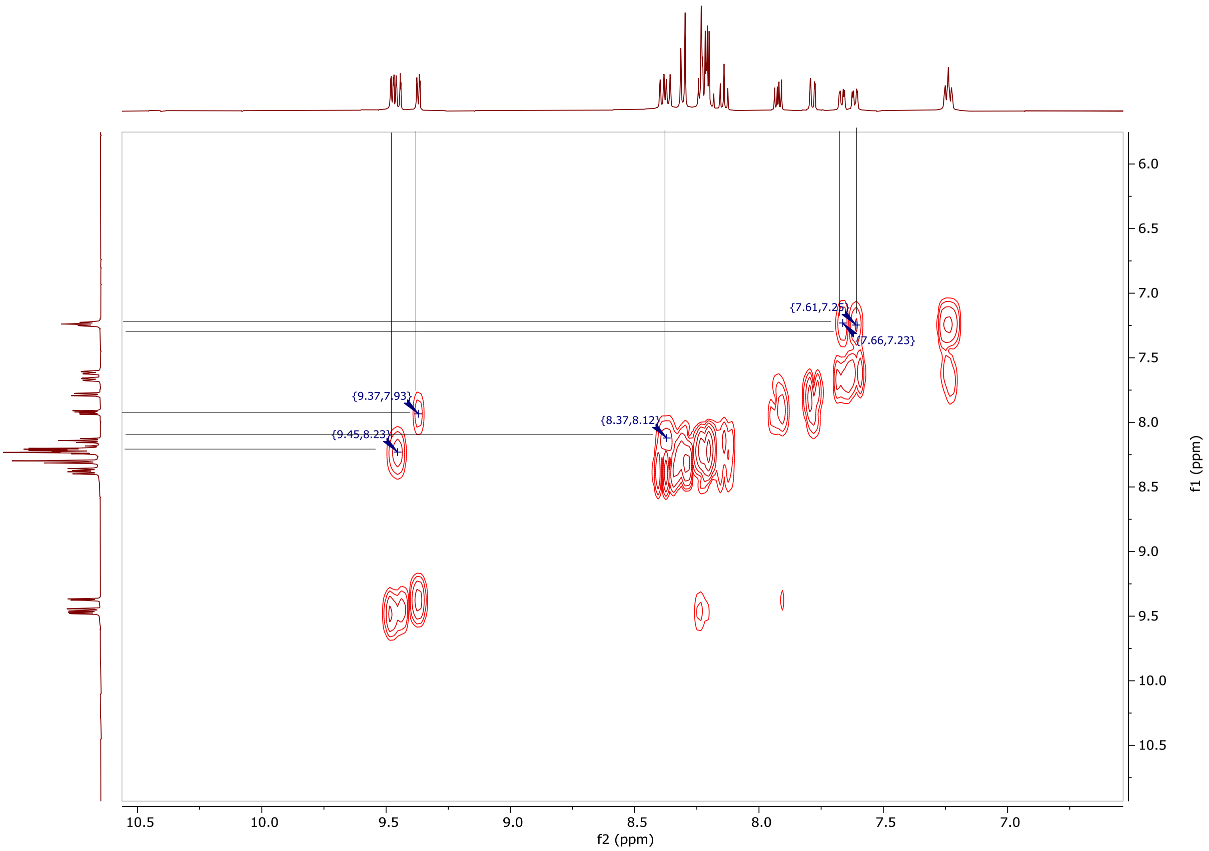
{7.61,7.25}
{7.66,7.23}
{9.37,7.93}
{8.37,8.12}
{9.45,8.23}
f2 (ppm)
f1 (ppm)

(c)

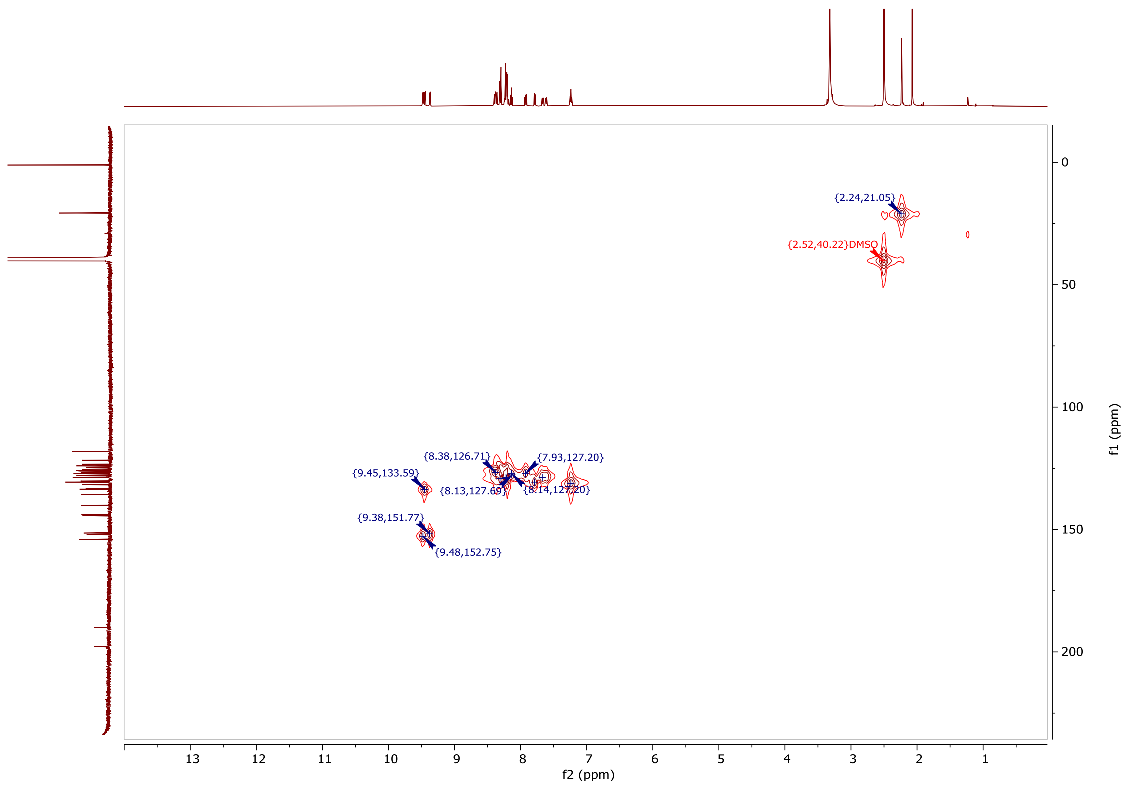

(d)

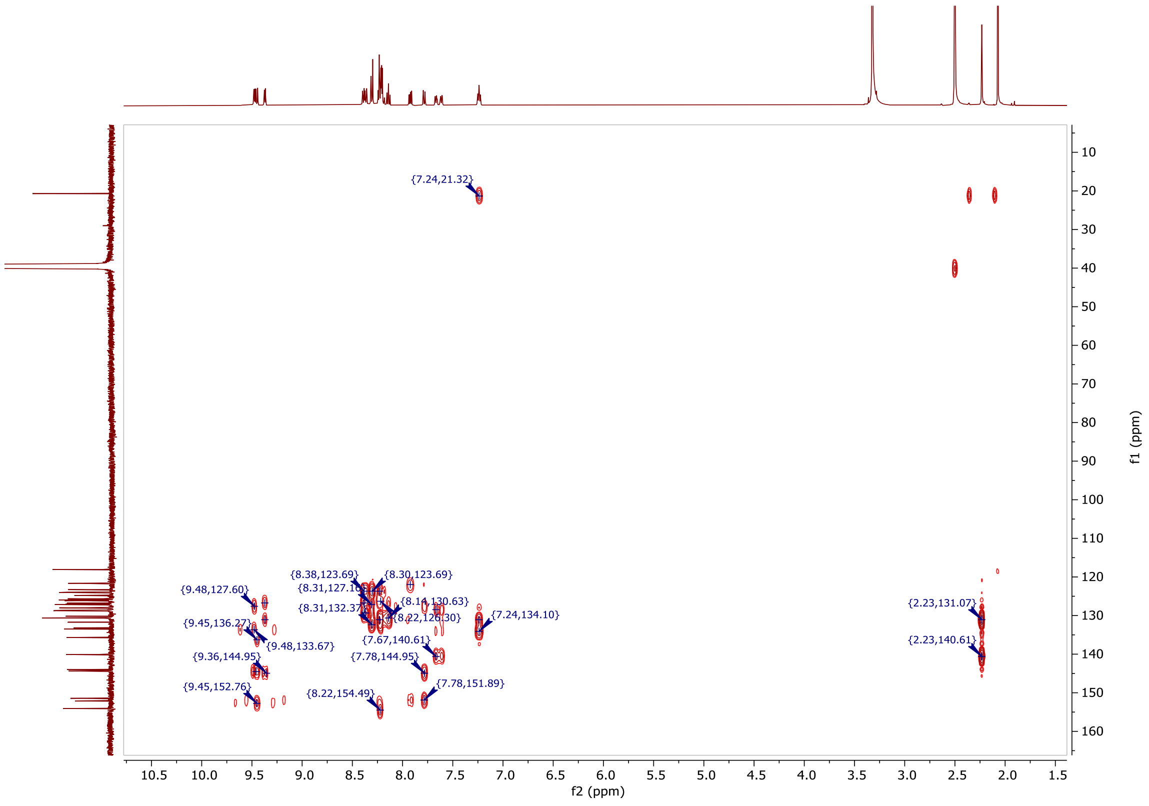

(e)

Figure S4. ^{1}H (a), ^{13}C (b), 2D ^{1}H–^{1}H COSY (c), ^{1}H–^{13}C HMQC (d) and ^{1}H–^{13}C HMBC (e) NMR spectra of complex **2** in DMSO-d_6.

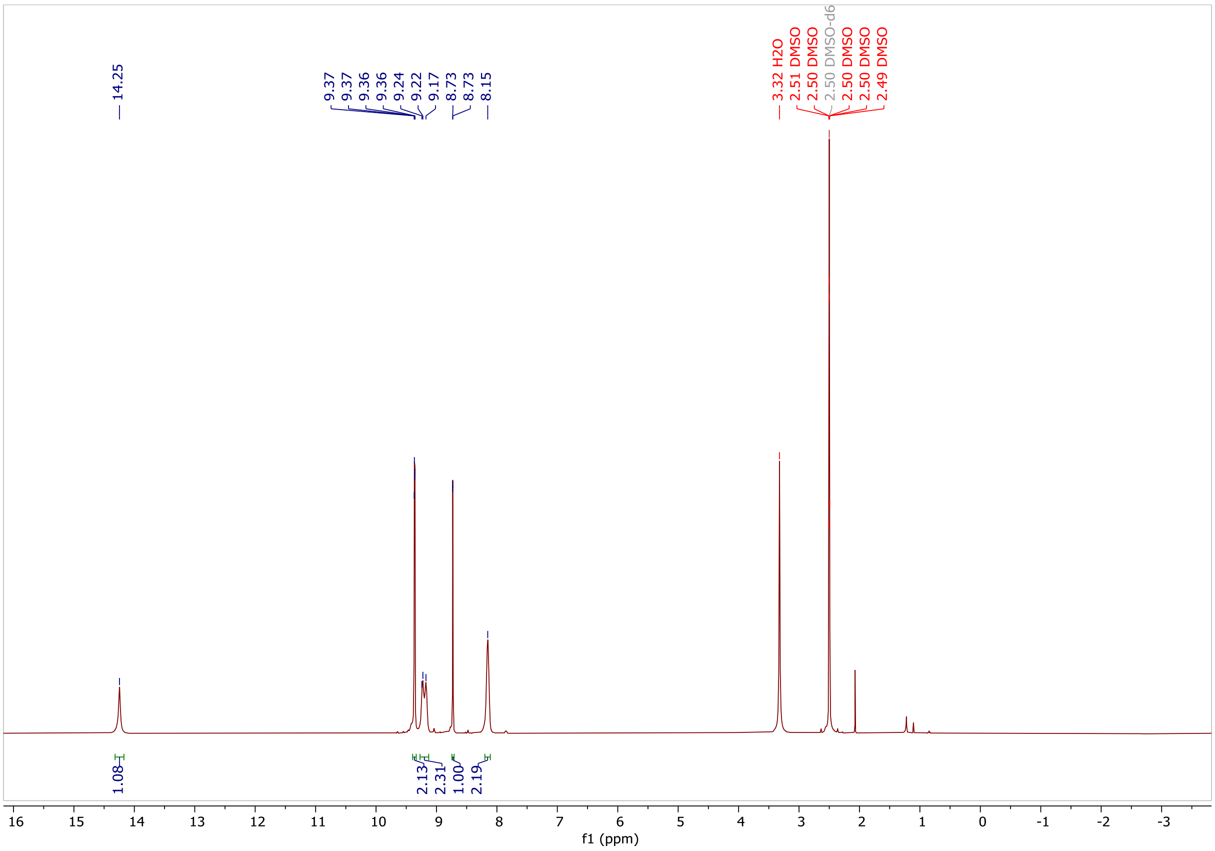
14.25
9.37
9.37
9.36
9.36
9.24
9.22
9.17
8.73
8.73
8.15
3.32 H2O
2.51 DMSO
2.50 DMSO
2.50 DMSO-d6
2.50 DMSO
2.50 DMSO
2.49 DMSO
1.08
2.13
2.31
1.00
2.19
f1 (ppm)

(a)

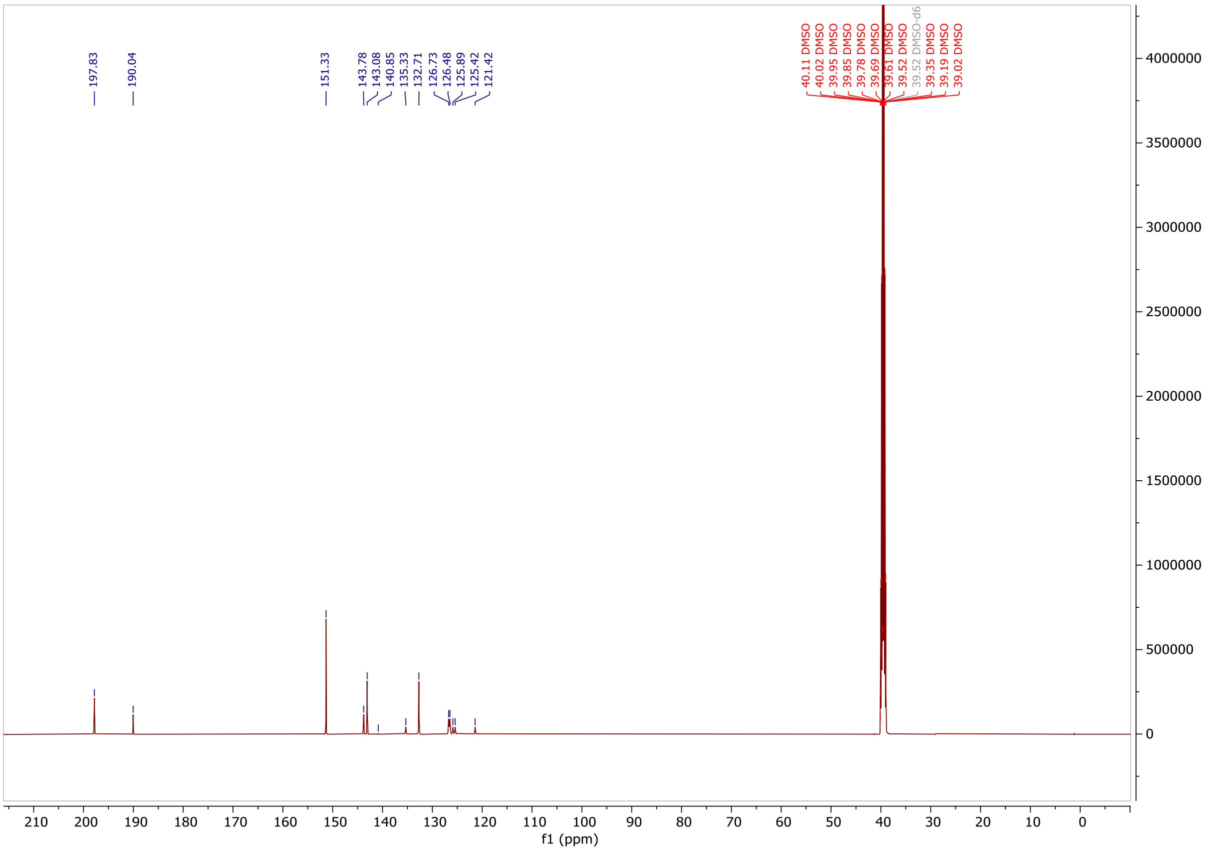
197.83
190.04
151.33
143.78
143.08
140.85
135.33
132.71
126.73
126.48
125.89
125.42
121.42
40.11 DMSO
40.02 DMSO
39.95 DMSO
39.85 DMSO
39.78 DMSO
39.69 DMSO
39.61 DMSO
39.52 DMSO
39.52 DMSO-d6
39.35 DMSO
39.19 DMSO
39.02 DMSO
f1 (ppm)

(b)

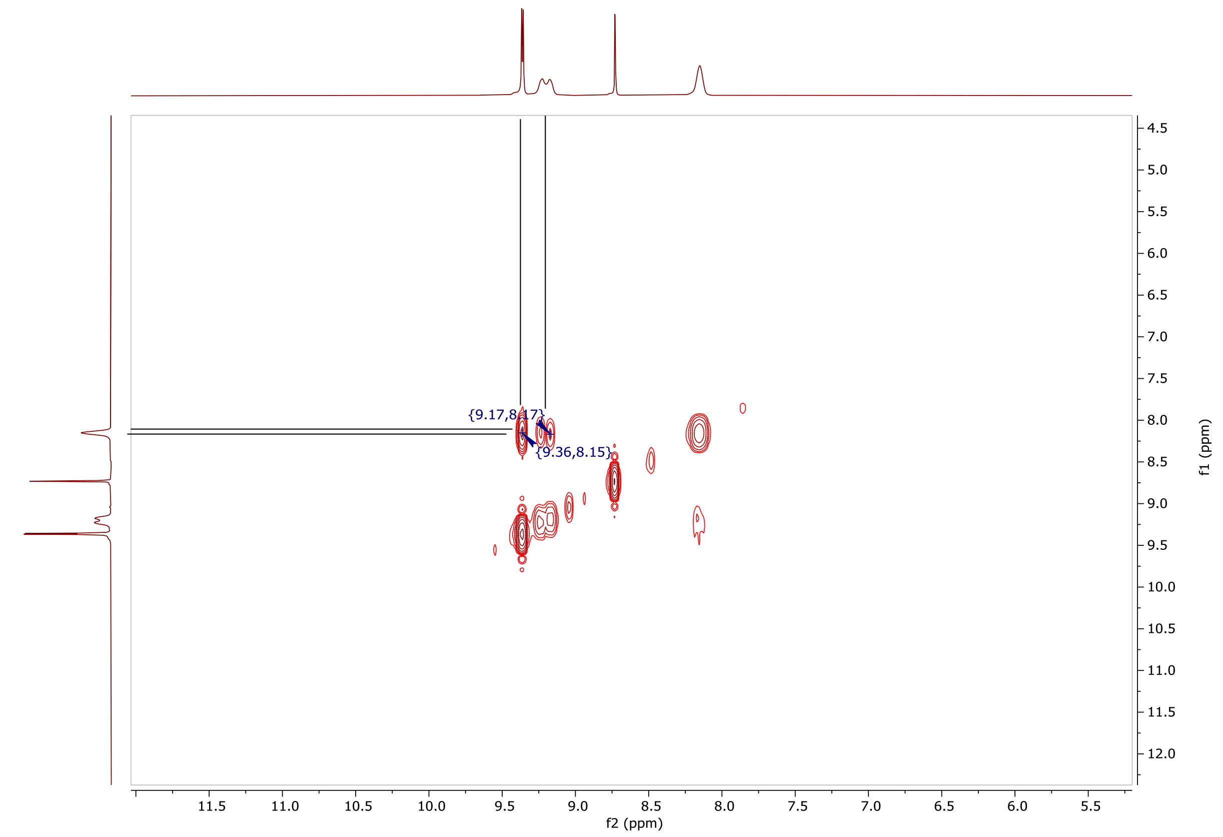
{9.17,8.17}
{9.36,8.15}
f2 (ppm)
f1 (ppm)

(c)

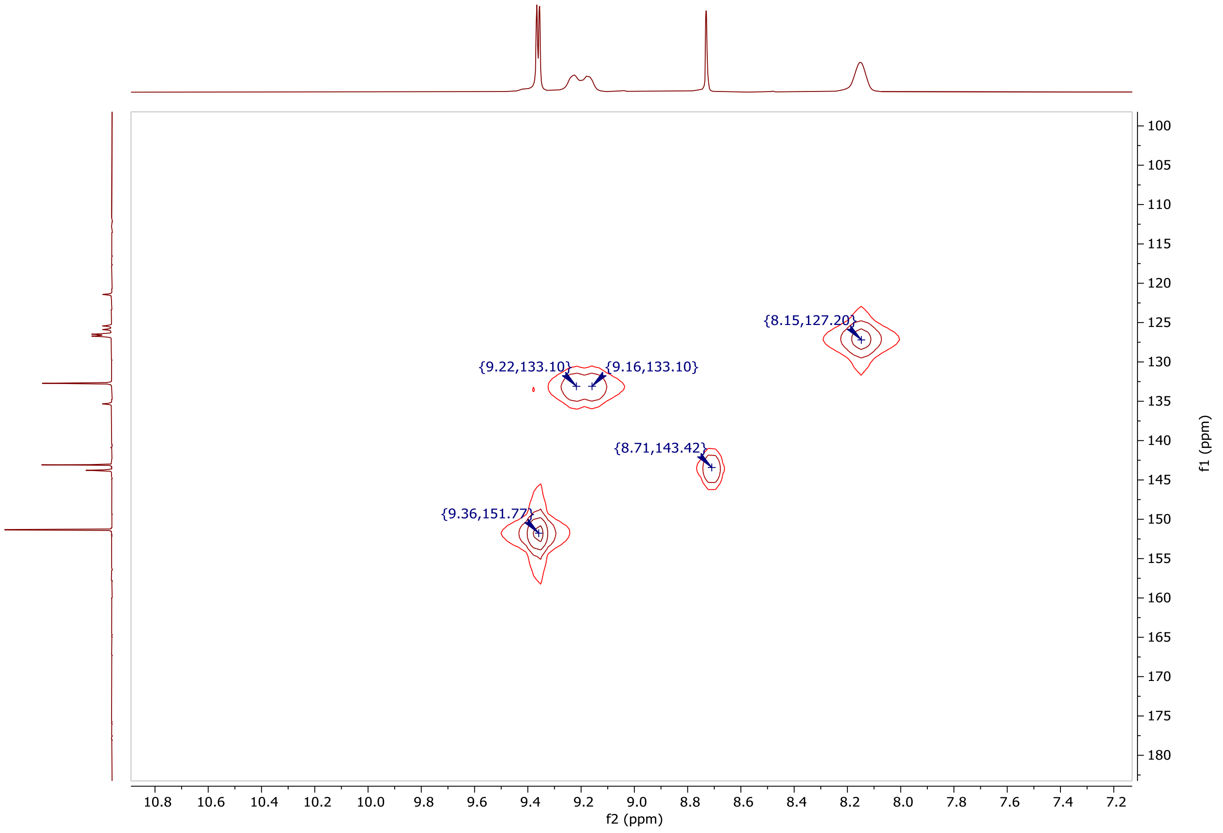
{8.15,127.20}
{9.22,133.10}
{9.16,133.10}
{8.71,143.42}
{9.36,151.77}
f2 (ppm)
f1 (ppm)

(d)

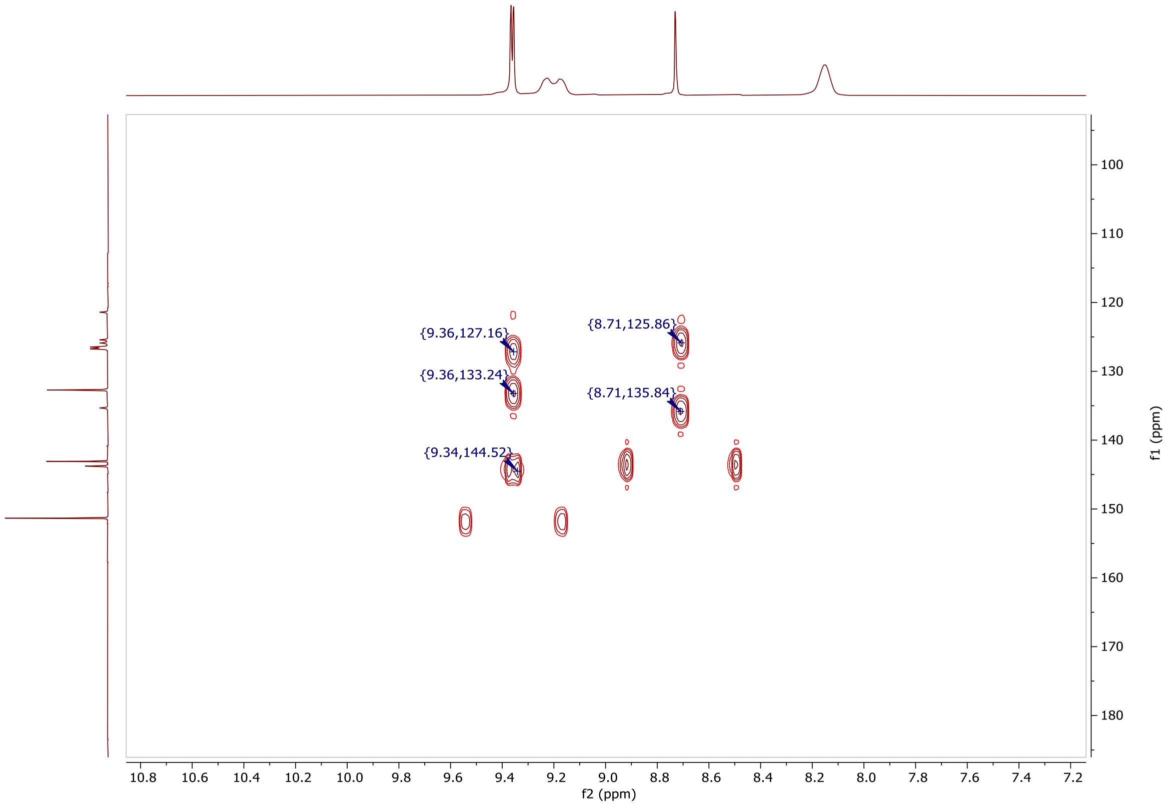

(e)

Figure S5. ^{1}H (a), ^{13}C (b), 2D ^{1}H–^{1}H COSY (c), ^{1}H–^{13}C HMQC (d) and ^{1}H–^{13}C HMBC (e) NMR spectra of complex **3** in DMSO-d$_6$.

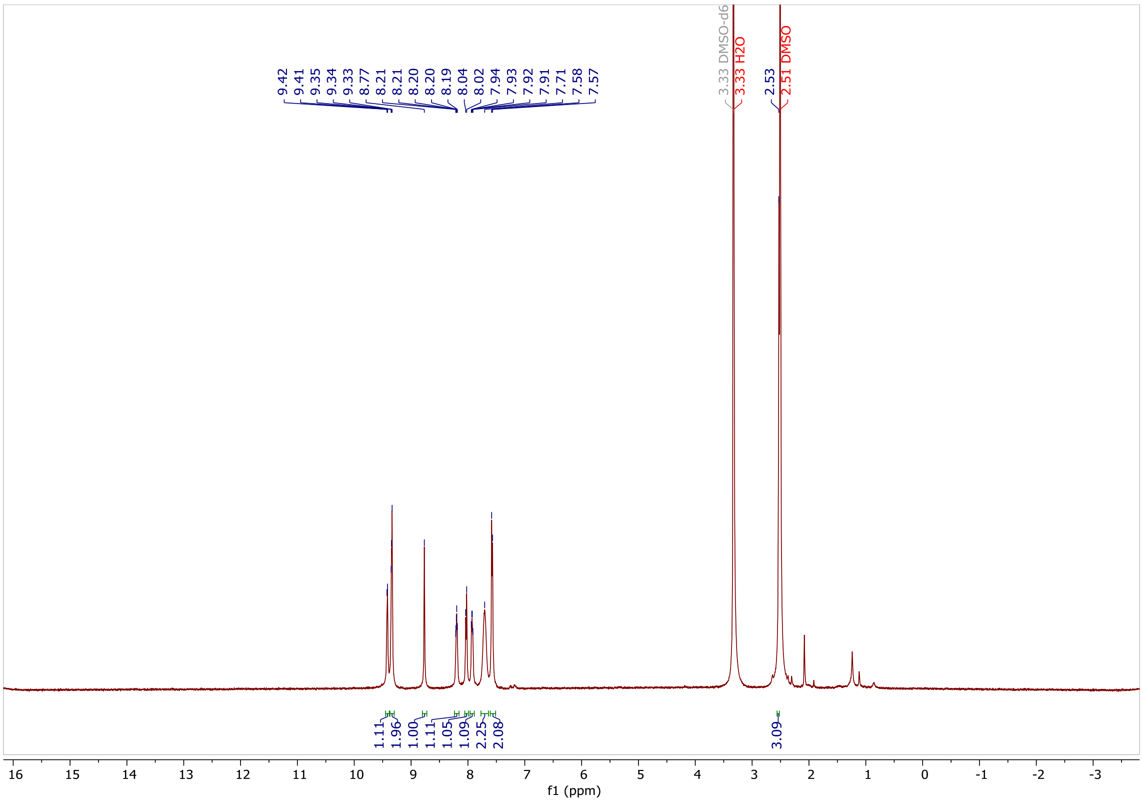

(a)

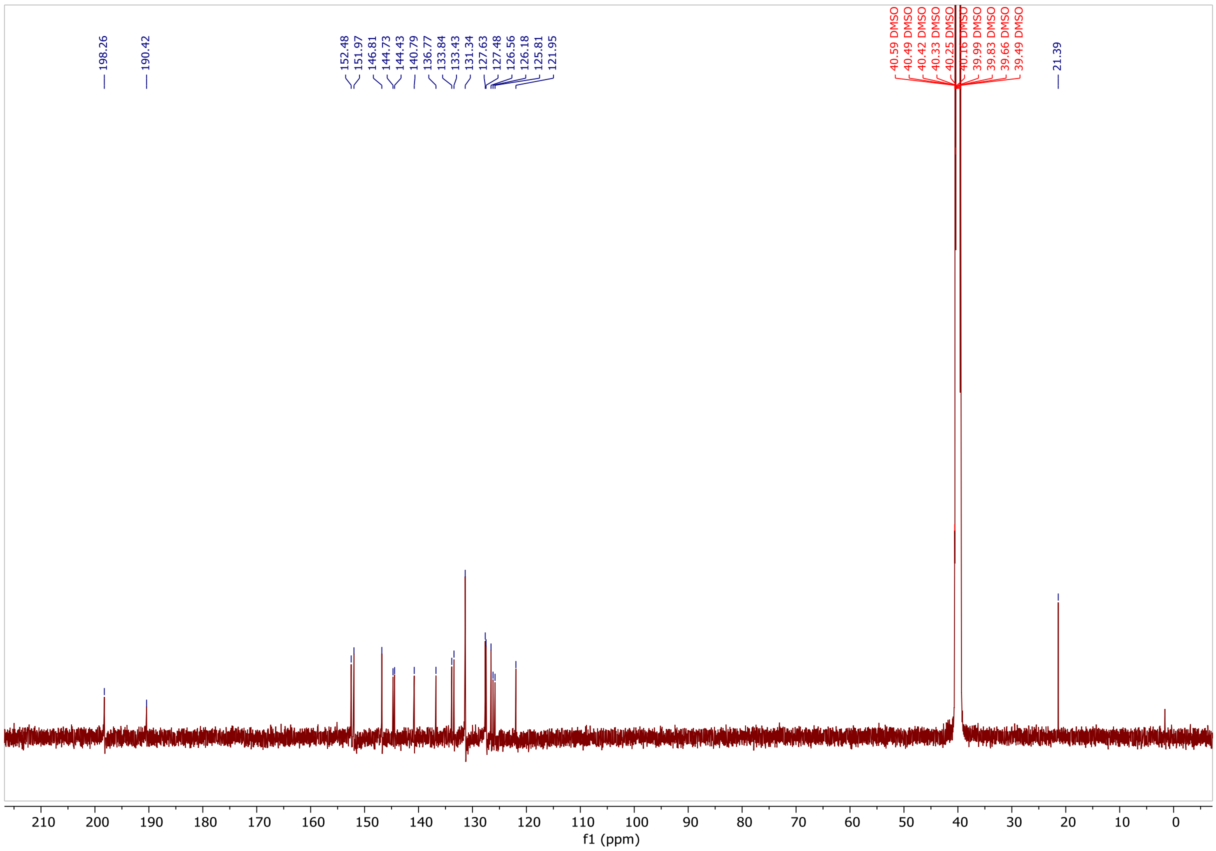

198.26
190.42
152.48
151.97
146.81
144.73
144.43
140.79
136.77
133.84
133.43
131.34
127.63
127.48
126.56
126.18
125.81
121.95
40.59 DMSO
40.49 DMSO
40.42 DMSO
40.33 DMSO
40.25 DMSO
40.16 DMSO
39.99 DMSO
39.83 DMSO
39.66 DMSO
39.49 DMSO
21.39
f1 (ppm)

(b)

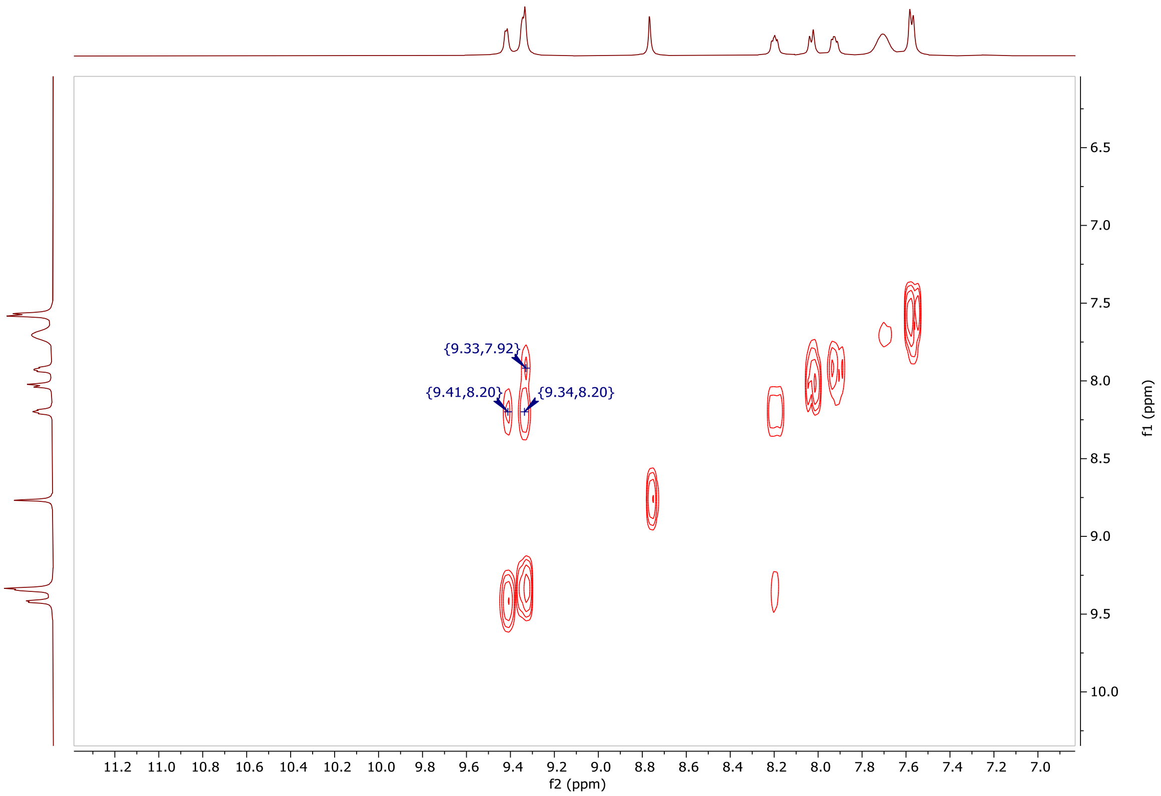

{9.33,7.92}
{9.41,8.20}
{9.34,8.20}
f2 (ppm)
f1 (ppm)

(c)

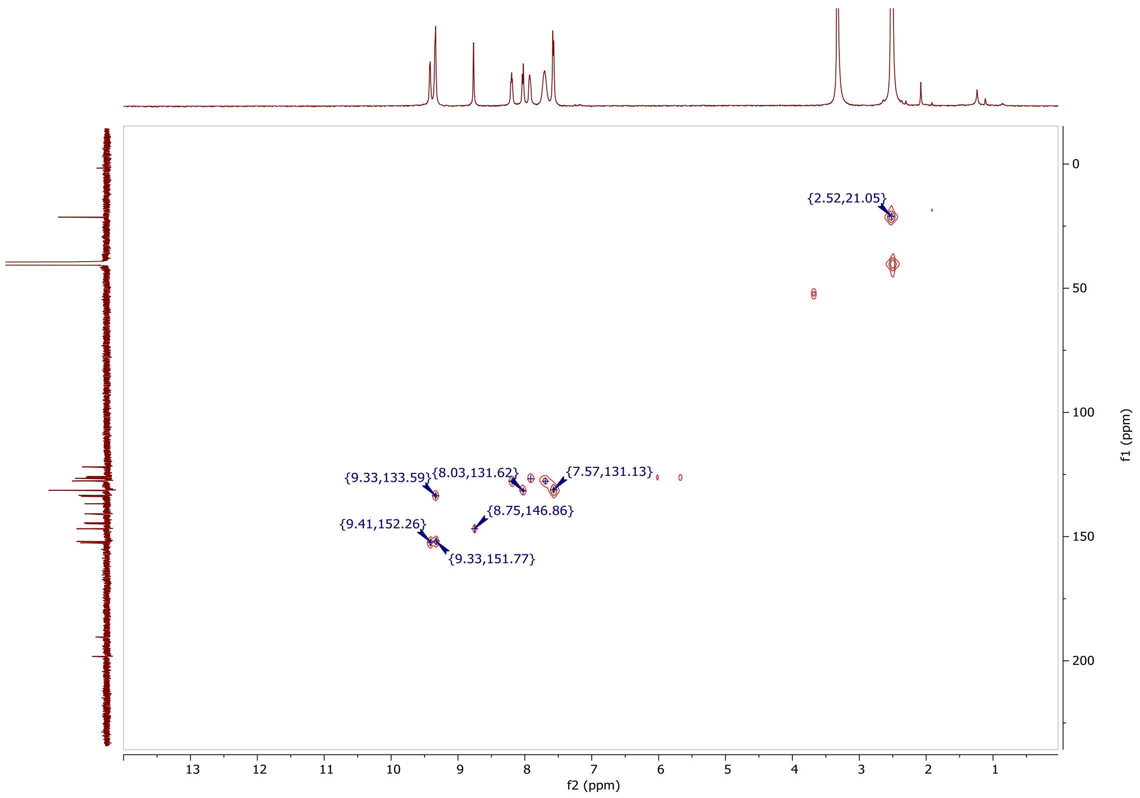

(d)

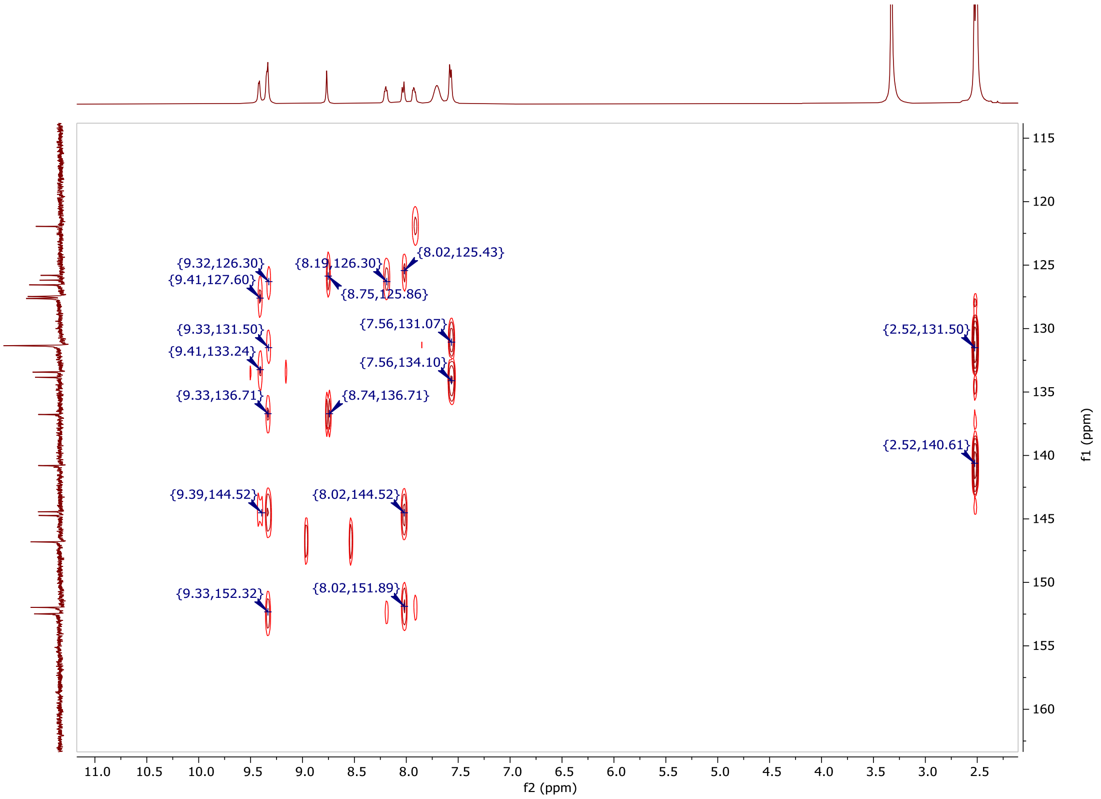

(e)

Figure S6. ^{1}H (a), ^{13}C (b), 2D ^{1}H–^{1}H COSY (c), ^{1}H–^{13}C HMQC (d) and ^{1}H–^{13}C HMBC (e) NMR spectra of complex **4** in DMSO-d_6.

X-Ray Analysis

Table S1. Crystal data and structure refinement.

**Complex**	**2**	**3 type A yellow block**	**3 type B yellow plate**	**4**
**Empirical formula**	$C_{80}H_{47}Cl_2N_9O_6Re_2$	$C_{16}H_8ClN_4O_3Re$	$C_{16}H_8ClN_4O_3Re$	$C_{23}H_{14}ClN_4O_3Re$
**Formula weight**	1673.56	525.91	525.91	616.03
**Temperature [K]**	295.0(2)	295.0(2)	295.0(2)	295.0(2)
**Wavelength [Å]**	0.71073	0.71073	0.71073	0.71073
**Crystal system**	Monoclinic	Monoclinic	Monoclinic	Monoclinic
**Space group**	$P2_1/c$	$P2_1/c$	$I2/a$	$I2/a$
**Unit cell dimensions [Å,°]**	a = 12.2939(7)	a = 7.0304(4)	a = 18.1459(11)	a = 23.5031(8)
	b = 11.9987(6)	b = 9.6319(4)	b = 7.0731(3)	b = 6.3774(2)
	c = 23.2308(12)	c = 22.6054(11)	c = 25.9490(14)	c = 28.1114(11)
	β = 90.788(4)	β = 96.392(5)	β = 108.553(7)	β = 104.304(4)
**Volume [Å^3]**	3426.5(3)	1521.23(13)	3157.4(3)	4083.0(3)
**Z**	2	4	8	8
**Density (calculated) [Mg/m^3]**	1.622	2.296	2.213	2.004
**Absorption coefficient [mm^{-1}]**	3.670	8.189	7.891	6.119
**F(000)**	1644	992	1984	2368
**Crystal size [mm]**	0.21 × 0.11 × 0.04	0.18 × 0.08 × 0.08	0.39 × 0.16 × 0.03	0.21 × 0.04 × 0.04
**θ range for data collection [°]**	3.51 to 25.05	3.45 to 25.05	3.35 to 25.05	3.32 to 25.05
**Index ranges**	$-14 \leq h \leq 14$<br>$-14 \leq k \leq 13$<br>$-27 \leq l \leq 22$	$-8 \leq h \leq 7$<br>$-11 \leq k \leq 11$<br>$-23 \leq l \leq 26$	$-20 \leq h \leq 21$<br>$-7 \leq k \leq 8$<br>$-27 \leq l \leq 30$	$-25 \leq h \leq 28$<br>$-7 \leq k \leq 7$<br>$-33 \leq l \leq 32$
**Reflections collected**	16142	6650	9616	17666
**Independent reflections**	6056 (R_{int} = 0.0589)	2698 (R_{int} = 0.0302)	2789 (R_{int} = 0.0603)	3611 (R_{int} = 0.0431)
**Completeness to 2θ**	99.6%	99.8%	99.7%	99.8%
**Min. and max. transm.**	0.518 and 1.000	0.560 and 1.000	0.155 and 1.000	0.208 and 1.000
**Data / restraints / parameters**	6056 / 0 / 462	2698 / 0 / 226	2789 / 0 / 226	3611 / 0 / 290
**Goodness-of-fit on F2**	1.022	1.085	1.043	1.071
**Final R indices [I>2σ(I)]**	R_1 = 0.0608<br>wR_2 = 0.1390	R_1 = 0.0291<br>wR_2 = 0.0606	R_1 = 0.0341<br>wR_2 = 0.0691	R_1 = 0.0277<br>wR_2 = 0.0591
**R indices (all data)**	R_1 = 0.1028<br>wR_2 = 0.1566	R_1 = 0.0363<br>wR_2 = 0.0630	R_1 = 0.0491<br>wR_2 = 0.0752	R_1 = 0.0359<br>wR_2 = 0.0622
**Largest diff. peak and hole [e Å^{-3}]**	1.42 and -0.62	1.35 and -0.66	1.65 and -0.95	0.96 and -0.581
**CCDC deposit no.**	2279356	2279357	2279358	2279359

Table S2. Selected bond lengths [Å] and angles [°].

Bond lengths	2	3a yellow block	3b yellow plate	4
**Re(1)–Cl(1)**	2.459(3)	2.4907(14)	2.4969(18)	2.4614(15)
**Re(1)–N(1)**	2.164(7)	2.181(4)	2.175(5)	2.177(4)
**Re(1)–N(2)**	2.164(8)	2.168(4)	2.165(5)	2.178(4)
**Re(1)–C(1)**	1.889(10)	1.914(7)	1.915(8)	1.911(6)
**Re(1)–C(2)**	1.896(15)	1.919(6)	1.912(8)	1.923(5)
**Re(1)–C(3)**	2.020(12)	1.903(6)	1.926(9)	1.983(7)
**Bond angles**				
**N(1)–Re(1)–Cl(1)**	83.96(19)	85.13(11)	87.65(13)	82.07(10)
**N(2)–Re(1)–Cl(1)**	85.7(2)	82.68(11)	80.98(14)	85.21(10)
**N(2)–Re(1)–N(1)**	75.0(3)	75.37(16)	75.36(18)	75.18(13)
**C(1)–Re(1)–Cl(1)**	90.3(3)	91.80(17)	90.8(2)	91.00(17)
**C(1)–Re(1)–N(1)**	171.8(4)	171.89(19)	173.6(2)	172.42(19)
**C(1)–Re(1)–N(2)**	98.7(4)	96.8(2)	98.2(3)	101.30(18)
**C(1)–Re(1)–C(2)**	89.4(5)	90.2(2)	90.7(3)	88.3(2)
**C(1)–Re(1)–C(3)**	91.7(4)	88.3(2)	88.4(3)	87.9(2)
**C(2)–Re(1)–Cl(1)**	92.5(3)	91.26(16)	93.1(2)	95.04(18)
**C(2)–Re(1)–N(1)**	96.8(4)	97.4(2)	95.6(3)	95.29(18)
**C(2)–Re(1)–N(2)**	171.7(4)	170.9(2)	169.3(2)	170.36(18)
**C(2)–Re(1)–C(3)**	90.8(5)	87.8(2)	91.5(3)	89.9(2)
**C(3)–Re(1)–Cl(1)**	176.2(3)	179.03(15)	175.4(2)	174.92(15)
**C(3)–Re(1)–N(1)**	93.7(3)	94.91(19)	92.6(3)	98.77(17)
**C(3)–Re(1)–N(2)**	90.7(4)	98.27(17)	94.6(3)	90.15(17)

Table S3. Short intra- and intermolecular hydrogen bonds.

D–H•••A	D–H [Å]	H•••A [Å]	D–A [Å]	D–H•••A [°]
**2**				
**C(20)–H(20)•••O(2)[a]**	0.93	2.36	3.221(15)	154.00
**3a**				
**N(4)–H(4)•••Cl(1)[a]**	0.86	2.36	3.187(5)	162.00
**C(5)–H(5)•••O(2)[b]**	0.93	2.59	3.502(7)	168.00
**C(6)–H(6)••Cl(1)[a]**	0.93	2.78	3.653(5)	156.00
**C(16)–H(16)•••O(3)[c]**	0.93	2.55	3.145(8)	122.00
**3b**				
**N(4)–H(4)•••Cl(1)[d]**	0.86	2.33	3.175(7)	169.00
**C(11)–H(11)•••N(3)[e]**	0.93	2.50	3.298(9)	144.00
**C(12)–H(12)••Cl(1)[f]**	0.93	2.77	3.557(8)	142.00
**4**				
**C(5)–H(5)•••Cl(1)[g]**	0.93	2.75	3.659(5)	166.00

symmetry codes: (a) = 1-x,1-y,1-z; (b) = 1-x,-y,1-z; (c) = 2-x,1-y,1-z; (d) = 1/2+x,2-y,z; (e) =3/2-x,1/2-y,1/2-z; (f) = 3/2-x,-1+y,1-z

Table S4. Short π•••π interactions.

Cg(I)•••Cg(J)	Cg(I)•••Cg(J) [Å]	α [°]	β [°]	γ [°]	Cg(I)-Perp [Å]	Cg(J)-Perp [Å]
**2**						
**Cg(1)•••Cg(2)[g]**	3.928(7)	14.6(5)	13.7	15.3	3.788(3)	3.816(6)
**3a**						
**Cg(3)•••Cg(1)[a]**	3.911(3)	2.6(3)	31.6	31.8	3.324(2)	3.3299(19)
**Cg(3)•••Cg(1)[c]**	3.504(3)	2.6(3)	9.1	7.0	3.478(2)	3.4608(19)
**Cg(1)•••Cg(4)[a]**	3.967(3)	2.5(2)	31.1	33.6	3.3055(19)	3.3962(19)
**Cg(1)•••Cg(4)[c]**	3.819(3)	2.5(2)	26.9	25.7	3.4422(19)	3.4060(19)
**3b**						
**Cg(3)•••Cg(3)[h]**	3.624(4)	0.0(4)	20.3	20.3	3.399(3)	3.398(3)
**Cg(3)•••Cg(4)[h]**	3.631(4)	0.8(3)	20.9	20.3	3.405(3)	3.392(2)
**4**						
**Cg(3)•••Cg(5)[a]**	3.622(3)	5.6(3)	21.4	20.7	3.387(2)	3.3719(18)
**Cg(1)•••Cg(5)[i]**	3.779(2)	7.2(2)	31.0	23.8	3.4568(18)	3.2392(18)

symmetry codes: (a) = 1-x,1-y,1-z; (c) = 2-x,1-y,1-z; (g) = 1-x,1/2+y,1/2-z; (h) = 3/2-x,3/2-y,1/2-z; (i) = x,-1+y,z; α = dihedral angle between Cg(I) and Cg(J); Cg(I)-Perp = Perpendicular distance of Cg(I) on ring J; Cg(J)-Perp = perpendicular distance of Cg(J) on ring I; β = angle Cg(I)→Cg(J) vector and normal to ring I; γ = angle Cg(I) →Cg(J) vector and normal to plane J; Cg(1) is the centroid of atoms = N1/C4/C5/C6/C7/C15; Cg(2) is the centroid of atoms = C21/C22/C23/C24/C25/C32; Cg(3) is the centroid of atoms = N3/C9/C8/N4/C16 ; Cg(4) is the centroid of atoms = C7/C8/C9/C10/C14/C15; Cg(5) is the centroid of atoms = N2/C13/C12/C11/C10/C14

Table S5. X–Y•••Cg(J)(π-ring) interactions.

X–Y•••Cg(J)	X(I)•••Cg(J) [Å]	X-Perp [Å]	γ [°]	Y-X(I)•••Cg(J) [°]
**2**				
**C(6)–H(6) ••• Cg(6)**	2.99	2.66	27.19	146.00
**Re(1)–Cl(1) ••• Cg(4)[a]**	3.753(5)	-3.611	15.78	168.25(14)
**3a**				
**C(1)-O(1) ••• Cg(5)[k]**	3.317(5)	-3.299	6.05	106.3(4)
**3b**				
**C(1)–O(1) ••• Cg(3)[l]**	3.521(6)	3.499	6.46	115.5(5)
**C(1)–O(1) ••• Cg(4)[l]**	3.922(6)	3.500	26.83	146.8(5)
**C(3)–O(3) ••• Cg(1)[m]**	3.348(6)	3.050	24.35	103.5(6)
**C(3)–O(3) ••• Cg(4)[m]**	3.383(5)	3.029	26.43	142.1(6)
**4**				
**Re(1)–Cl(1) ••• Cg(1)[m]**	3.866(2)	3.534	23.90	112.54(5)
**C(2)–O(2) ••• Cg(7)[m]**	3.513(5)	3.096	28.20	105.0(3)

symmetry codes: (a) = 1-x,1-y,1-z; (k) = 1-x,-1/2+y,1/2-z; (l) = -1/2+x,1-y,z; (m) = 3/2-x,y,1-z; γ = angle X(I)→Cg(J) vector and normal to plane J. Cg(1) is the centroid of atoms = N1/C4/C5/C6/C7/C15; Cg(3) is the centroid of atoms = N3/C9/C8/N4/C16; Cg(4) is the centroid of atoms = C7/C8/C9/C10/C14/C15; Cg(5) is the centroid of atoms = N2/C13/C12/C11/C10/C14; Cg(6) is the centroid of atoms = C33/C34/C35/C36/C37/C38; Cg(7) is the centroid of atoms = C17/C18/C19/C20/C21/C22

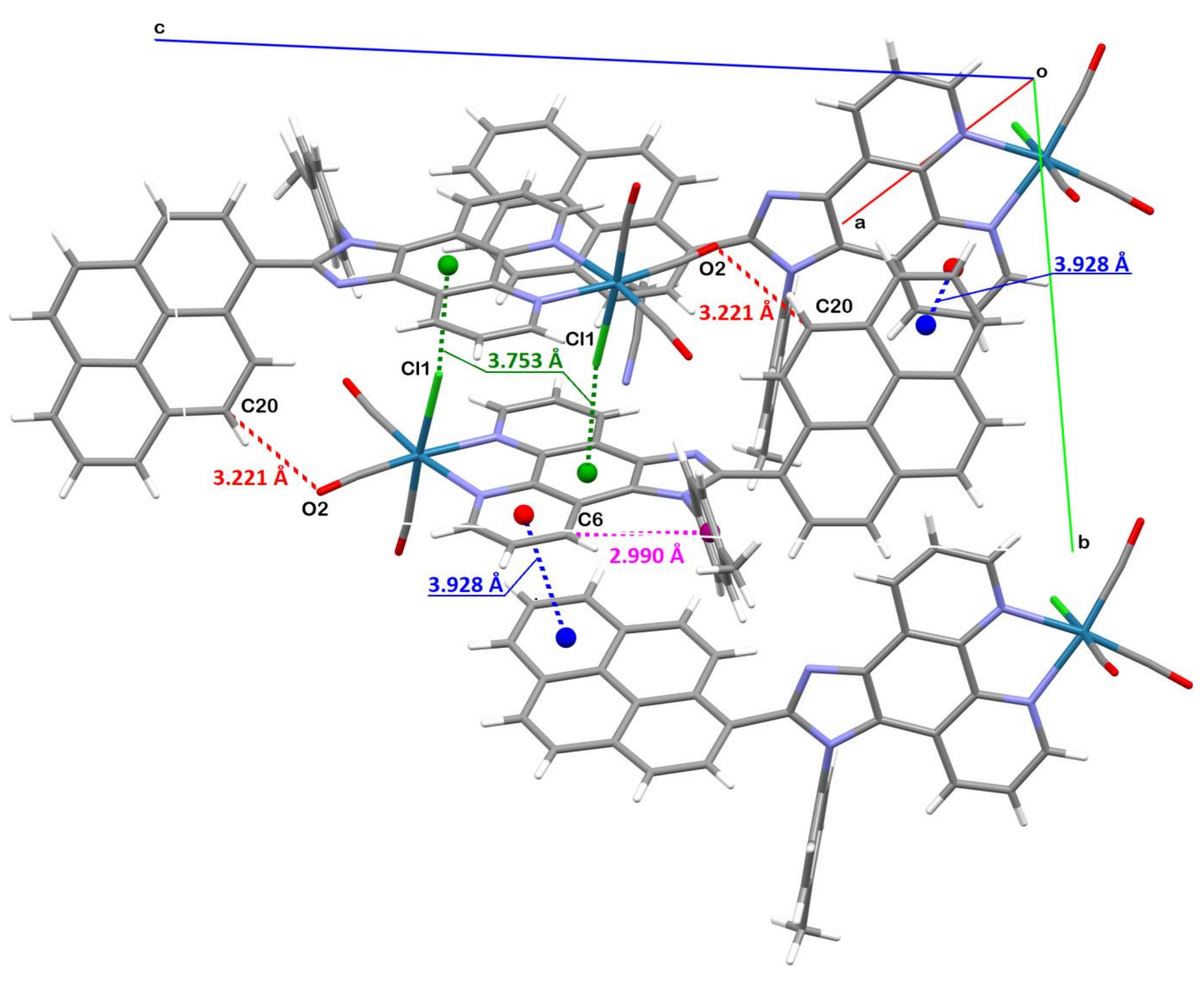

c
o
a
b
O2
3.221 Å
C20
3.928 Å
Cl1
Cl1
3.753 Å
C20
3.221 Å
O2
C6
2.990 Å
3.928 Å

2

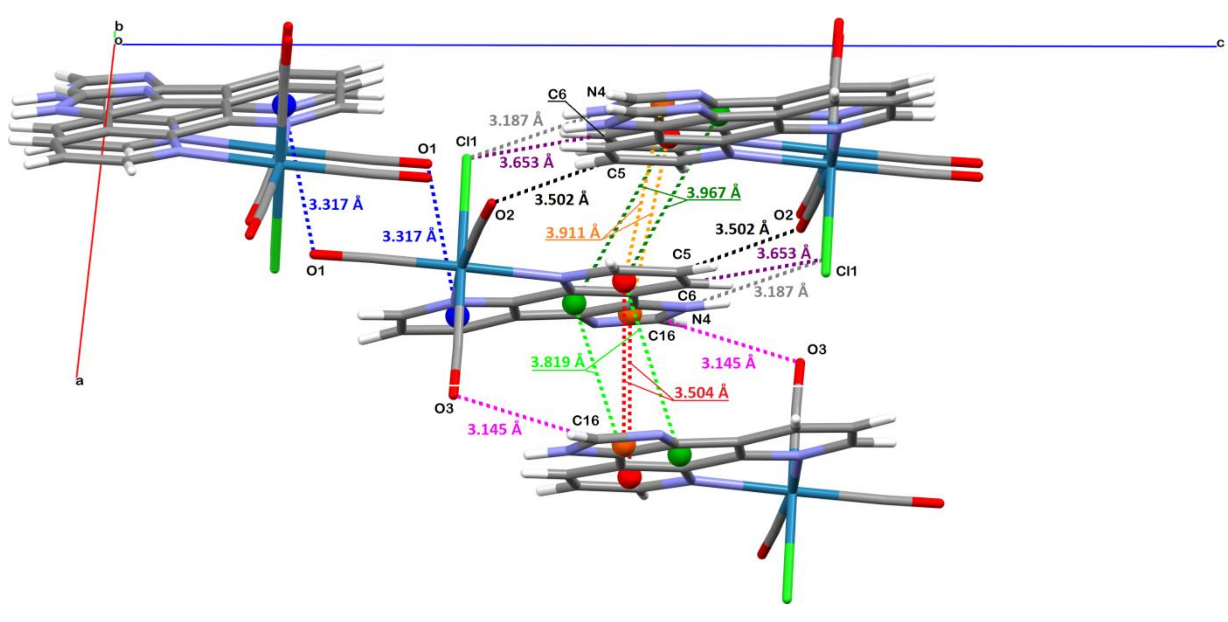

b
o
c
a
C6
N4
3.187 Å
Cl1
O1
3.653 Å
C5
3.502 Å
3.967 Å
3.317 Å
3.317 Å
O2
3.911 Å
3.502 Å
O2
O1
C5
3.653 Å
Cl1
C6
3.187 Å
N4
C16
O3
3.819 Å
3.145 Å
3.504 Å
O3
3.145 Å
C16

3a

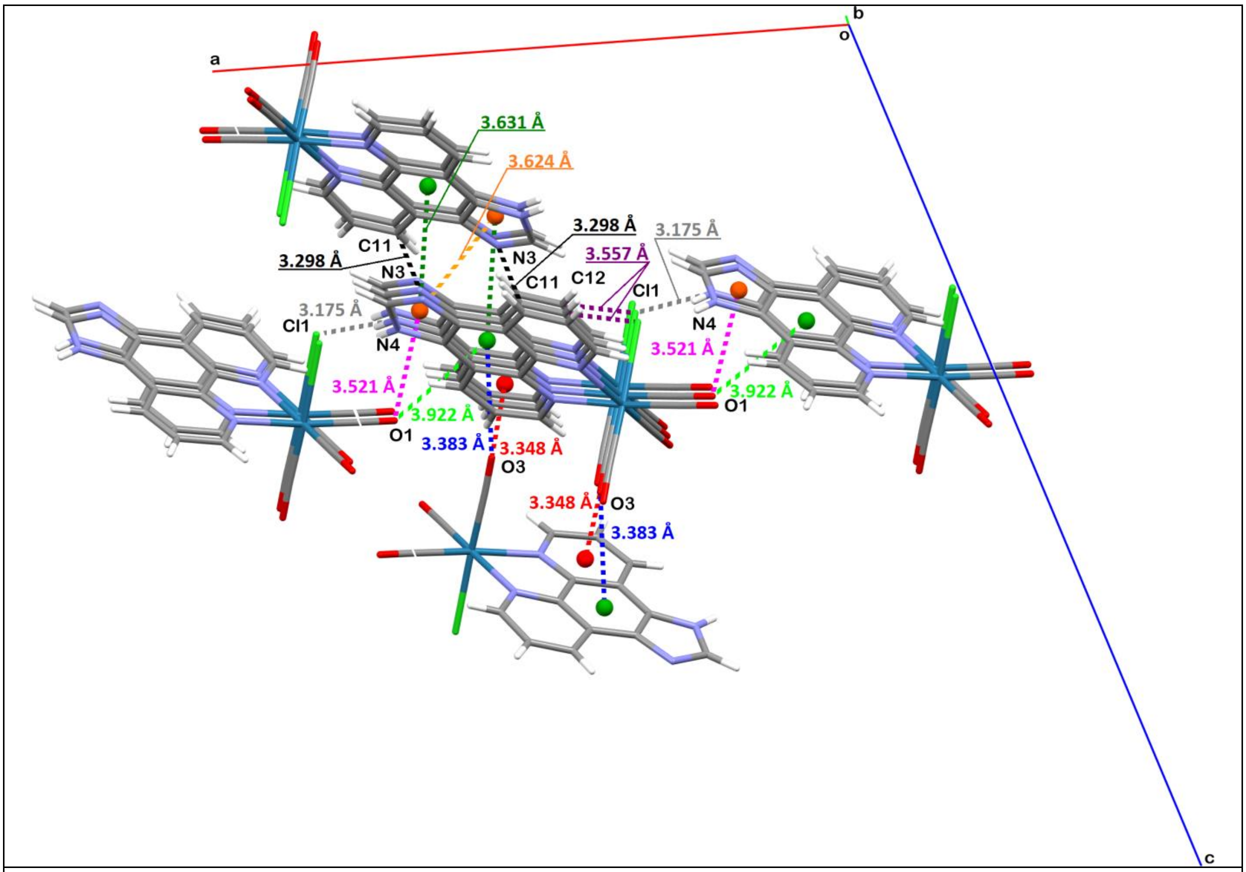

3b

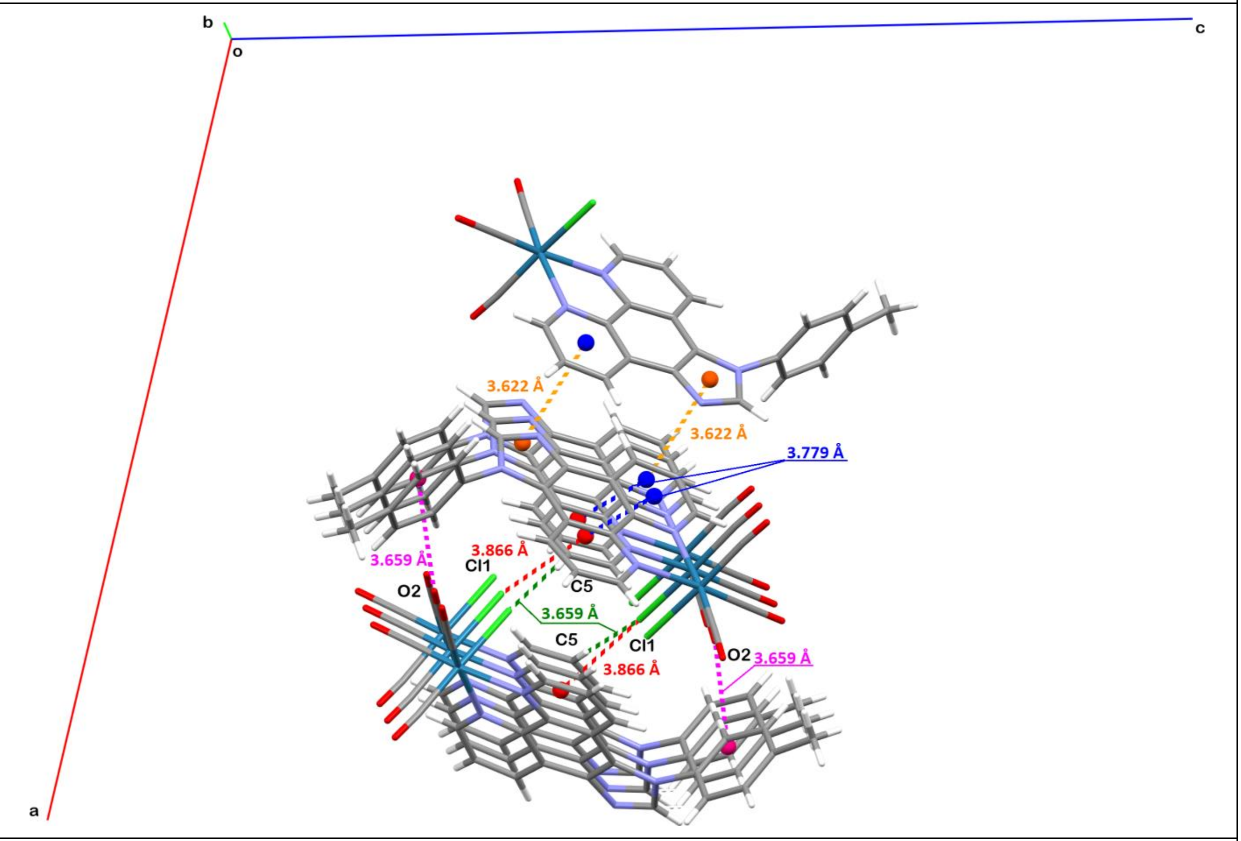

4

Figure S7. Inter- and intramolecular short contacts (for more details see Tables S3–S5).

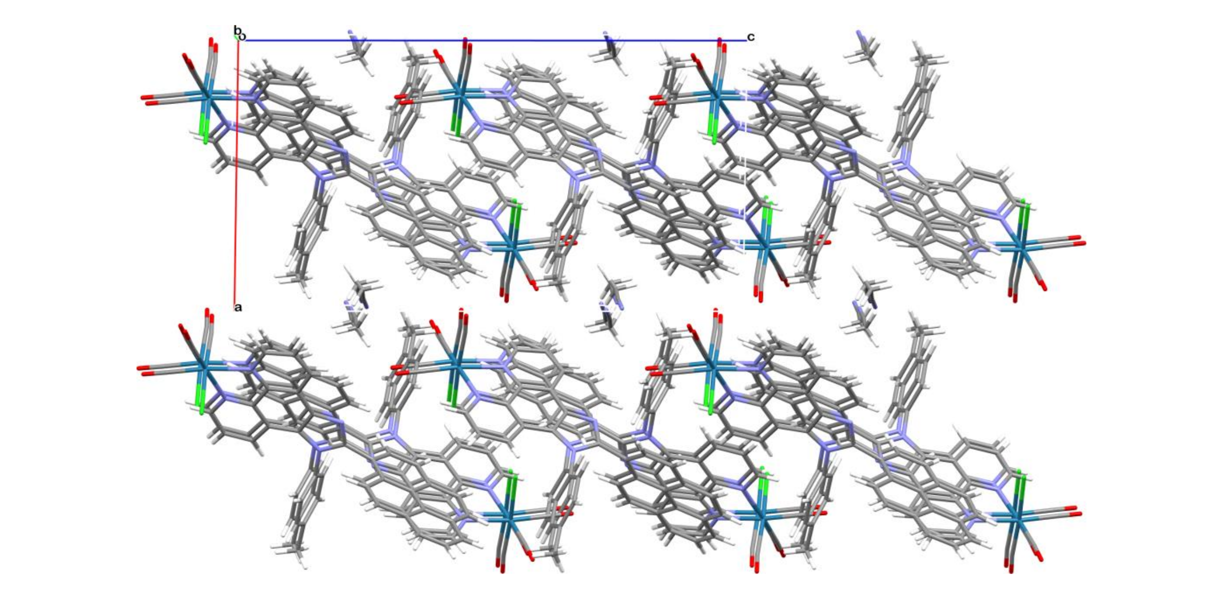

2

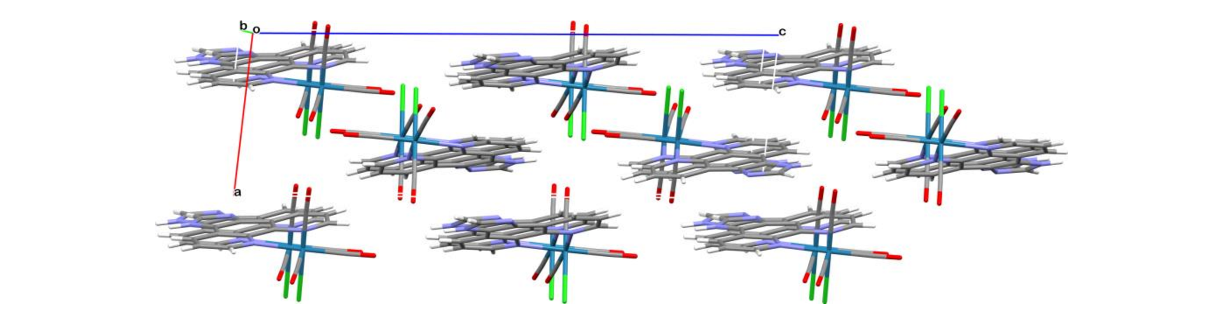

3a

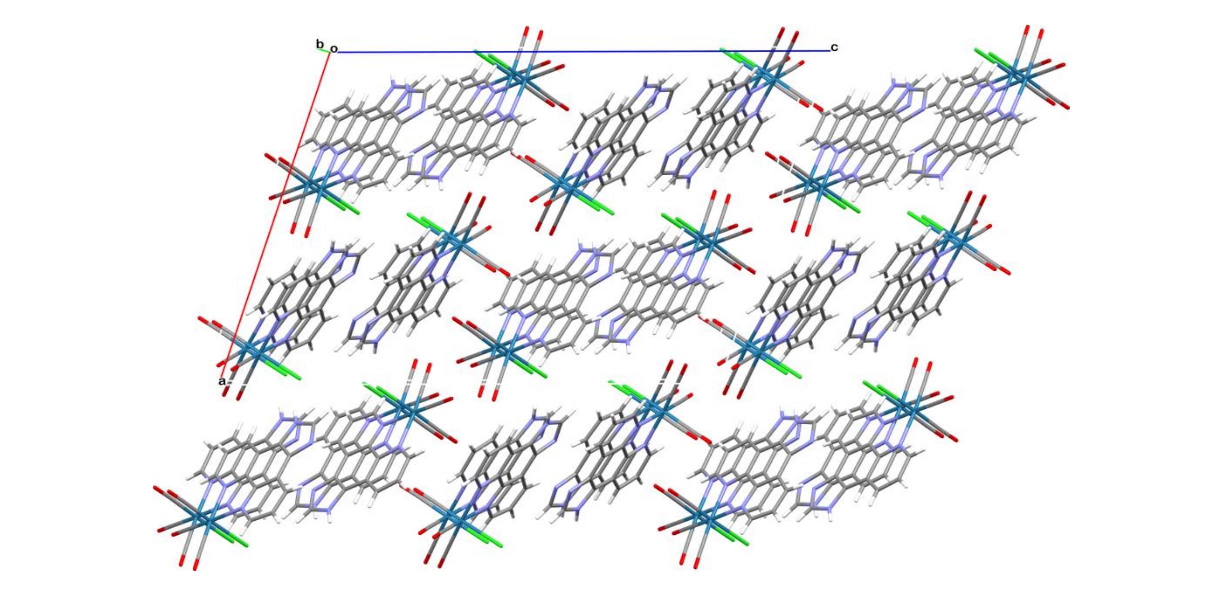

3b

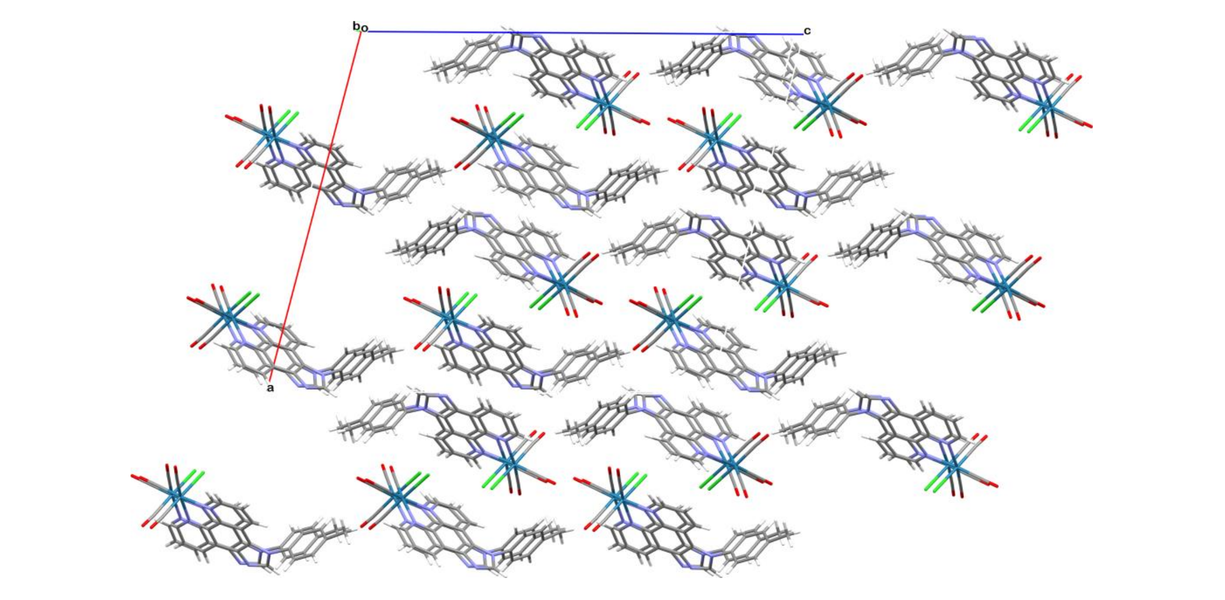

4

Figure S8. Molecular packing displayed down the *b* axis.

DFT calculations

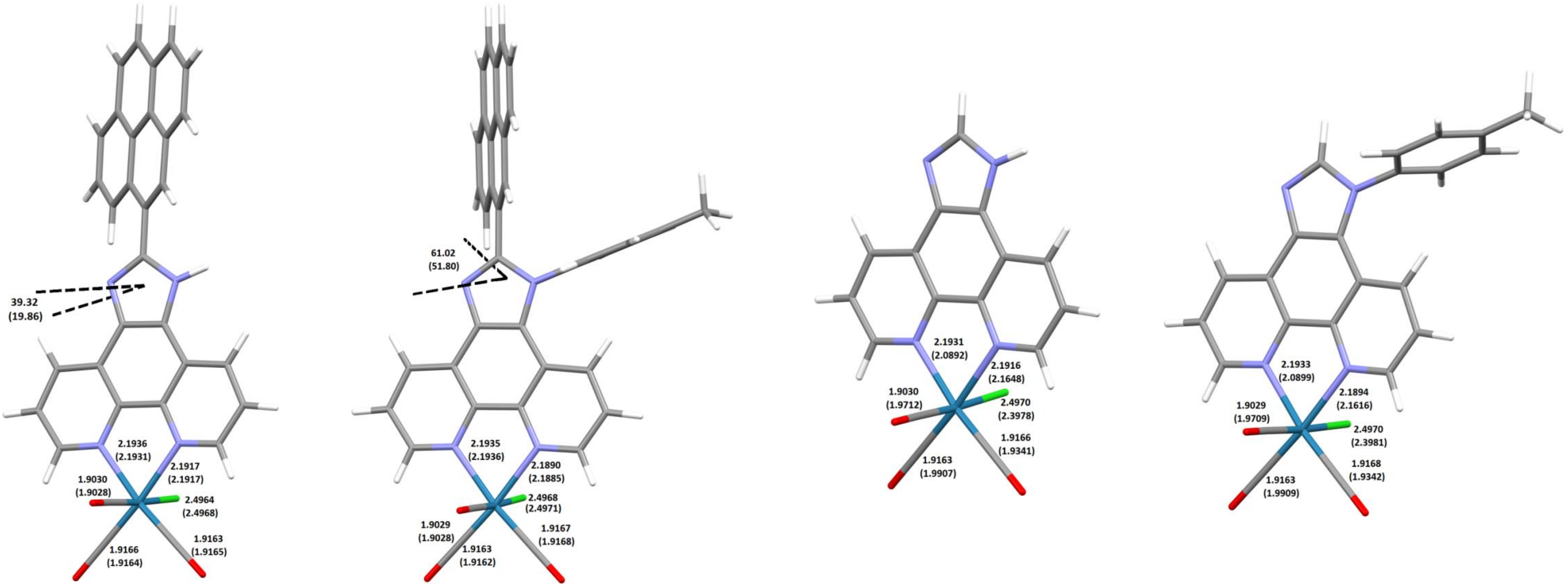

1 **2** **3** **4**

Figure S9. Selected values of bond lengths and the dihedral angles between the mean planes of imphen and pyrene in DFT-optimized structures in singlet ground state (S_0, without brackets) and lowest triplet-excited state (T_1, in brackets).

Table S6. The calculated and experimental values of selected bond lengths of complexes **1–4** in DFT-optimized singlet ground state (S_0) and lowest triplet-excited state (T_1) structures and the dihedral angle between the mean planes of imphen and pyrene moieties in **1–2**.

	**1**		**2**			**3**				**4**		
	**S_0**	**T_1**	**S_0**	**T_1**	**Xray**	**S_0**	**T_1**	**XrayA**	**XrayB**	**S_0**	**T_1**	**Xray**
**Re(1)–Cl(1)**	2.4964	2.4968	2.4968	2.4971	2.459(3)	2.4970	2.3978	2.4907(14)	2.4969(18)	2.4970	2.3981	2.461(2)
**Re(1)–C(1)**	1.9166	1.9164	1.9163	1.9162	1.889(10)	1.9163	1.9907	1.914(7)	1.915(8)	1.9163	1.9909	1.910(5)
**Re(1)–C(2)**	1.9163	1.9165	1.9167	1.9168	1.896(15)	1.9166	1.9341	1.919(6)	1.912(8)	1.9168	1.9342	1.924(5)
**Re(1)–C(3)**	1.9030	1.9028	1.9029	1.9028	2.020(12)	1.9030	1.9712	1.903(6)	1.926(9)	1.9029	1.9709	1.982(7)
**Re(1)–N(1)**	2.1936	2.1931	2.1935	2.1936	2.164(7)	2.1931	2.0892	2.181(4)	2.175(5)	2.1933	2.0899	2.177(4)
**Re(1)–N(2)**	2.1917	2.1917	2.1890	2.1885	2.164(8)	2.1916	2.1648	2.168(4)	2.165(5)	2.1894	2.1616	2.178(3)
**Angle imphen–pyrene**	39.32	19.86	61.02	51.80	70.57	-	-	-	-	-	-	-

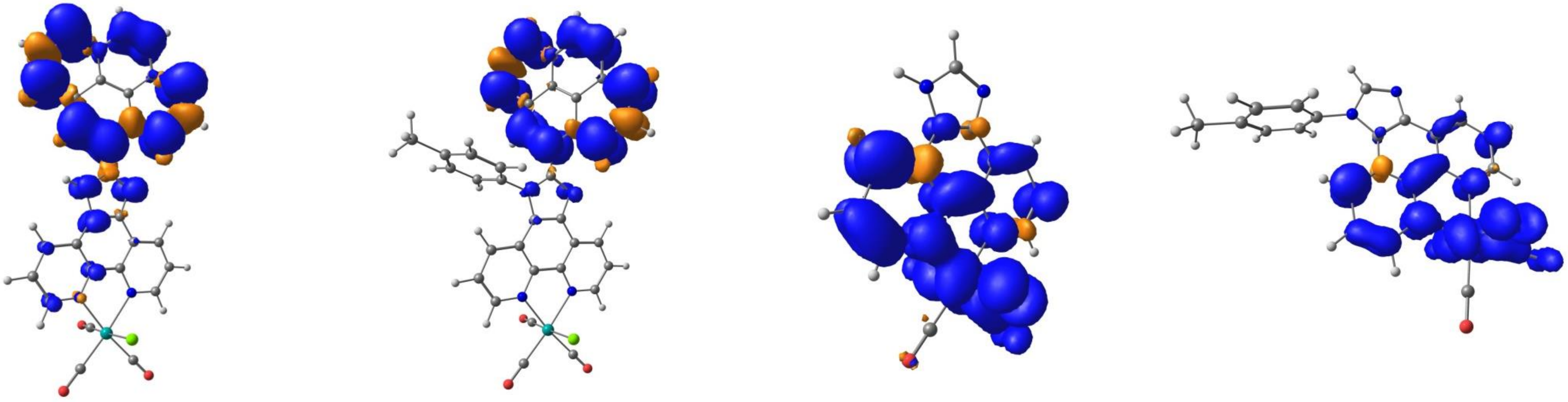

1 **2** **3** **4**

Figure S10. DFT calculated spin density maps for complexes **1–4**.

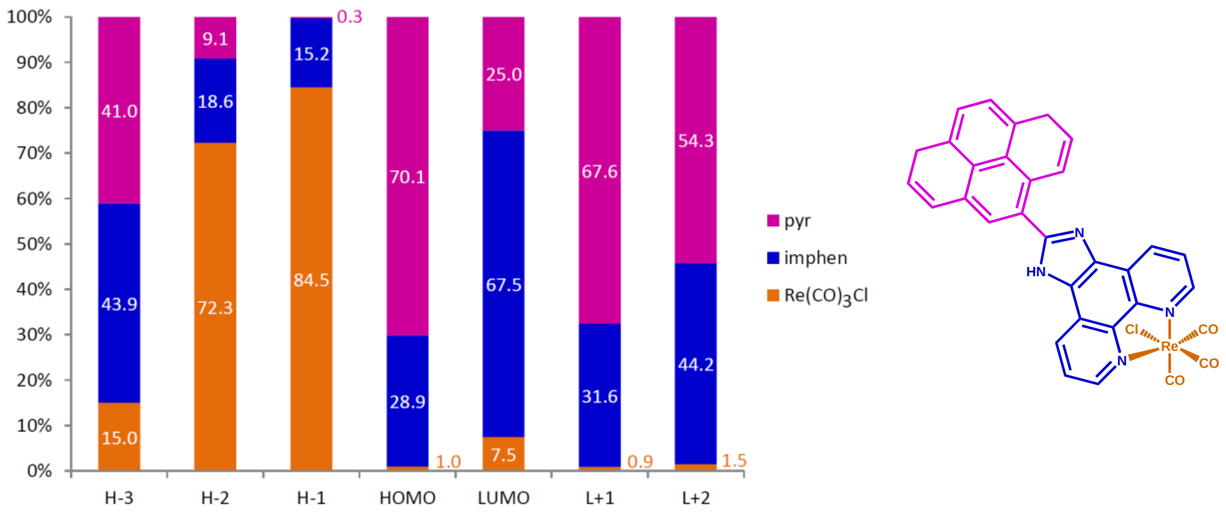

(a)

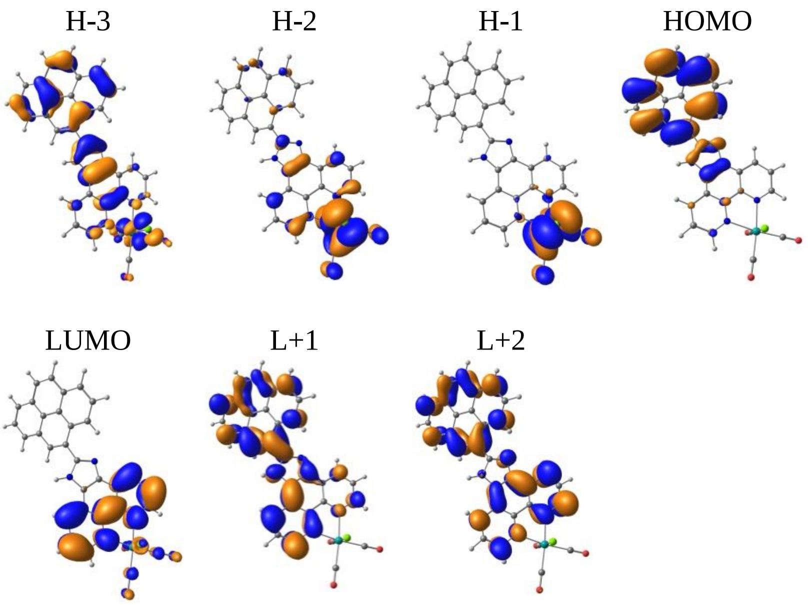

(b)

Figure S11. Percentage contribution of selected molecular fragments to the frontier molecular orbitals (a) and the isosurface plots of the orbitals (b) of complex **1**.

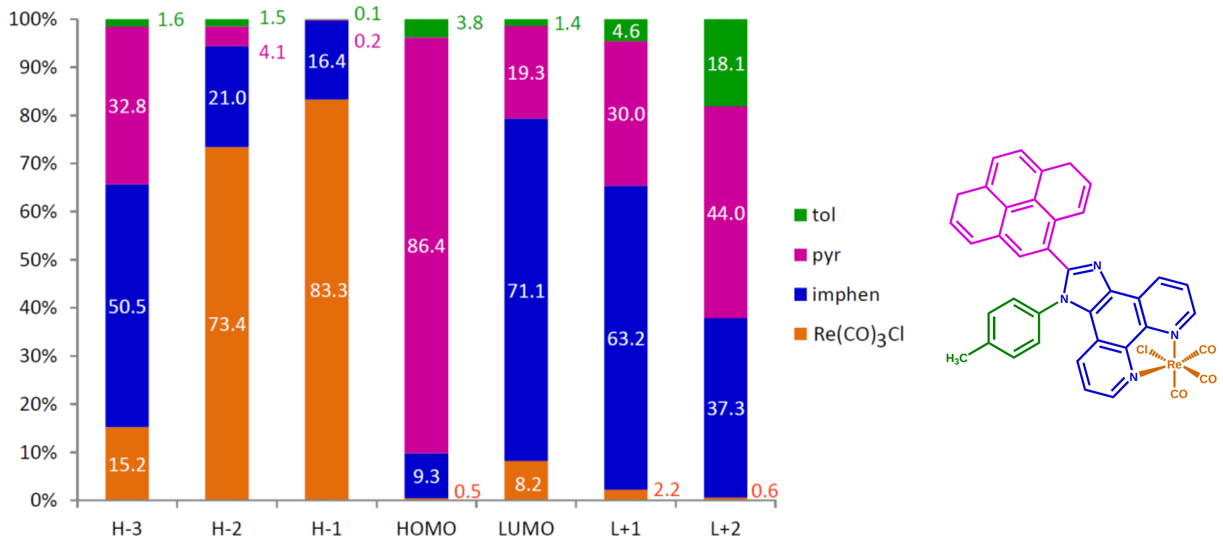

(a)

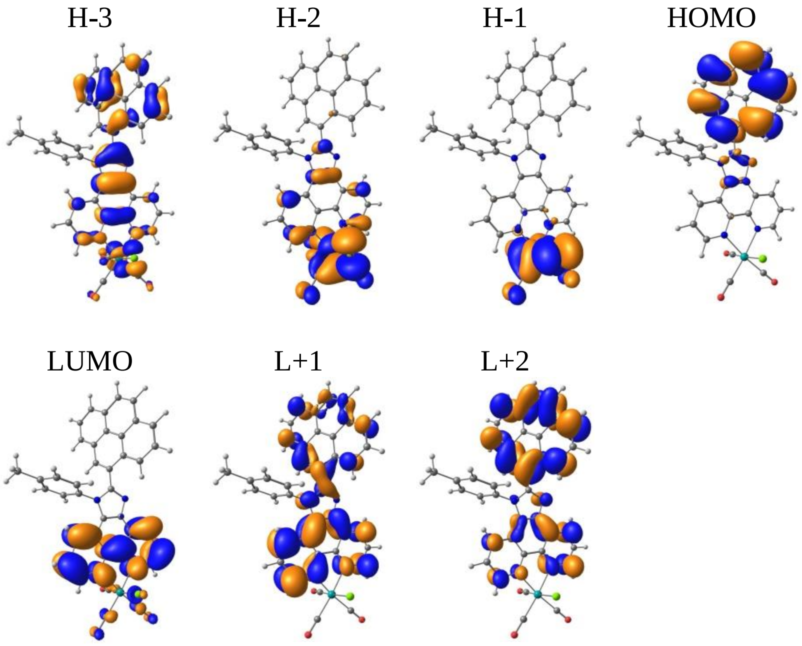

(b)

Figure S12. Percentage contribution of selected molecular fragments to the frontier molecular orbitals (a) and the isosurface plots of the orbitals (b) of complex **2**.

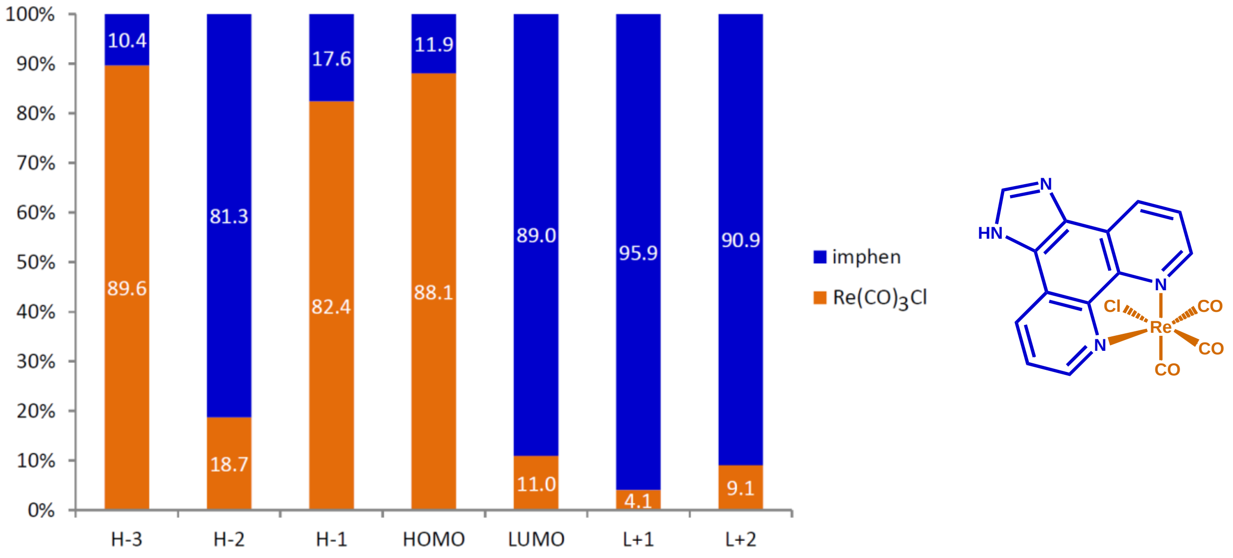

(a)

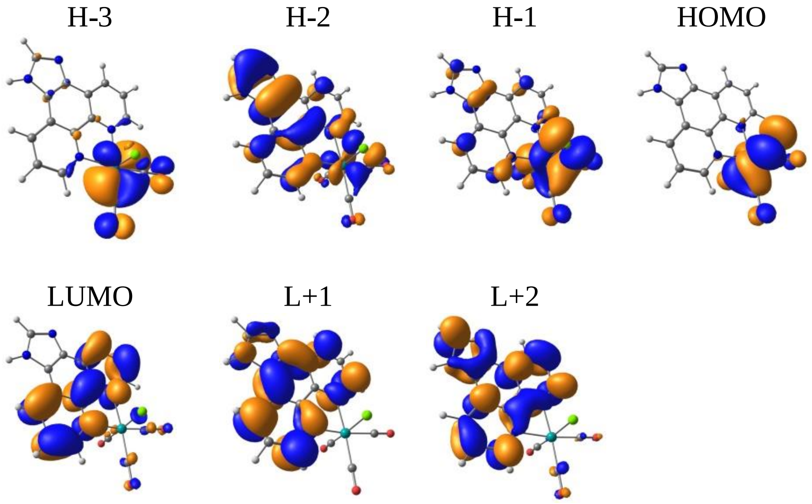

(b)

Figure S13. Percentage contribution of selected molecular fragments to the frontier molecular orbitals (a) and the isosurface plots of the orbitals (b) of complex **3**.

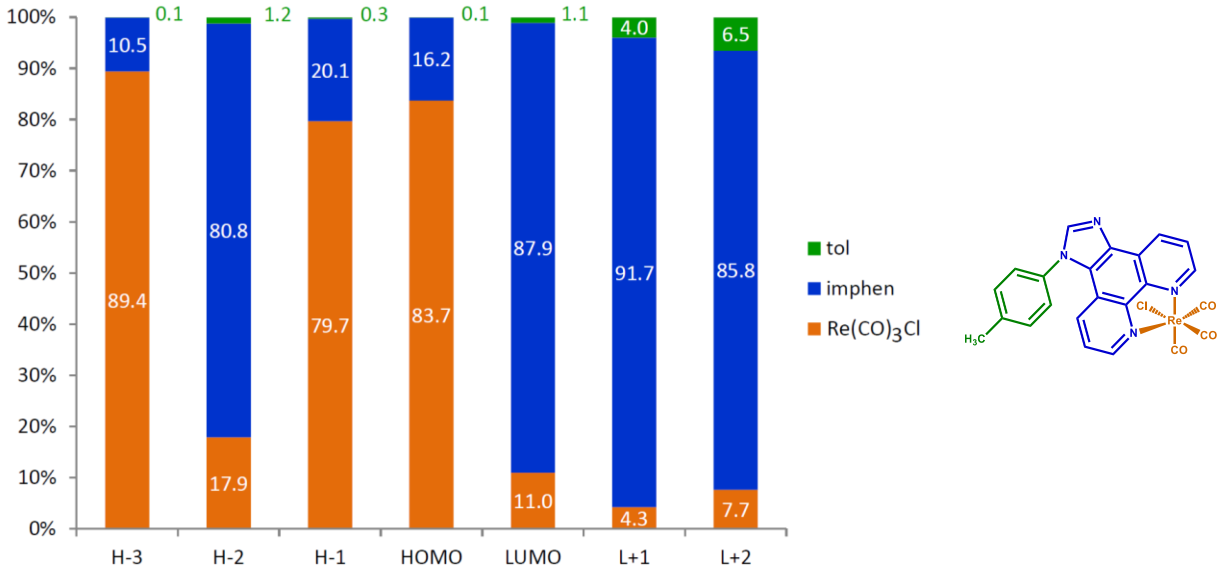

(a)

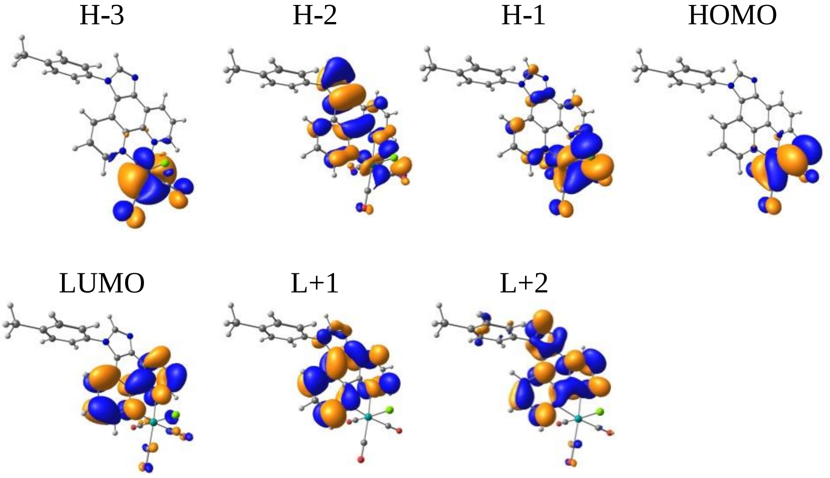

(b)

Figure S14. Percentage contribution of selected molecular fragments to the frontier molecular orbitals (a) and the isosurface plots of the orbitals (b) of complex **4**.

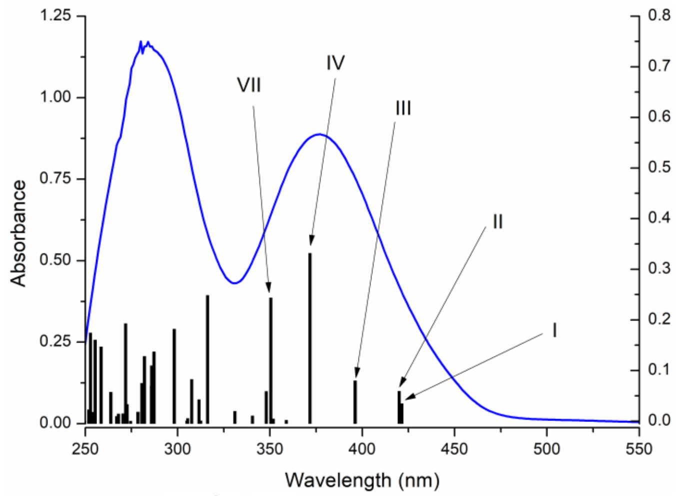

NTO I: 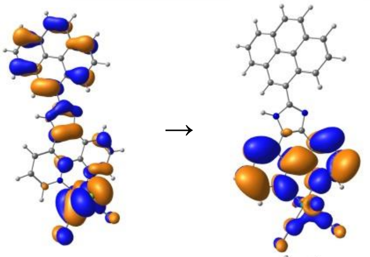

Osc. Strength 0.0349

H-1 → LUMO (60%)
HOMO → LUMO (34%)
H-2 → LUMO (5%)

NTO II: 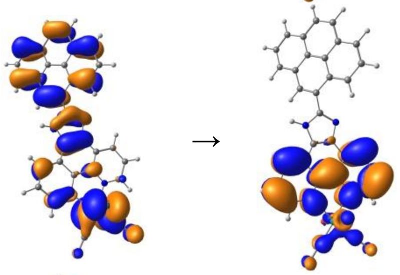

Osc. Strength 0.0594

H-2 → LUMO (10%)
H-1 → LUMO (39%)
HOMO → LUMO (49%)

NTO III: 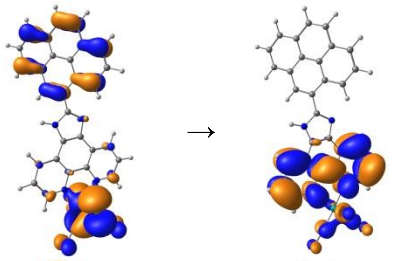

Osc. Strength 0.0802

H-2 → LUMO (80%)
HOMO → LUMO (12%)
H-3 → LUMO (5%)

NTO IV: 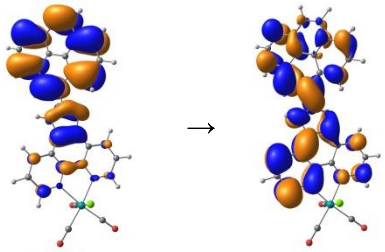

Osc. Strength 0.3318

HOMO → L+1 (93%)

NTO VII: 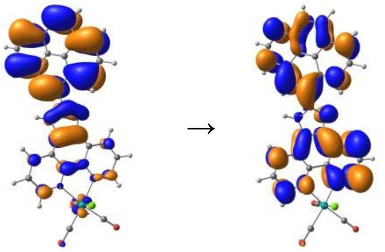

Osc. Strength 0.2438

HOMO → L+2 (88%)

Figure S15. Calculated NTOs of significant low-energy transitions for complex **1.**

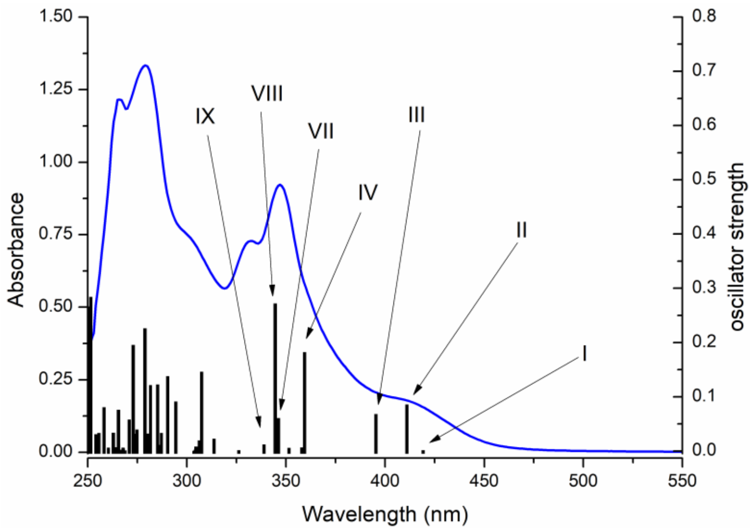

NTO I: 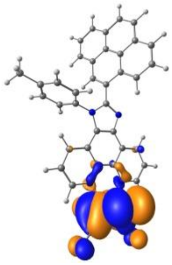 → 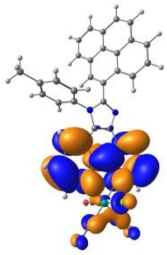

Osc. Strength 0.0017

H-1 → LUMO (99%)

NTO II: 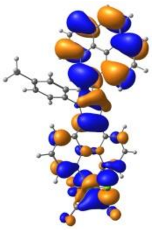 → 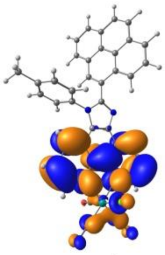

Osc. Strength 0.0858

HOMO → LUMO (74%)
H-2 → LUMO (24%)

NTO III: 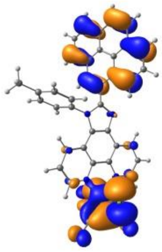 → 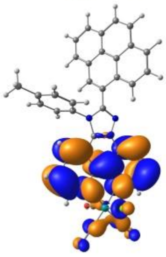

Osc. Strength 0.0681

H-2 → LUMO (73%)
HOMO → LUMO (22%)

NTO IV: 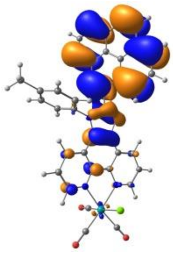 → 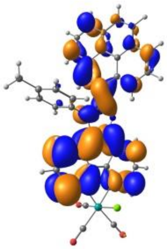

Osc. Strength 0.182

HOMO → L+1 (92%)

NTO VII: 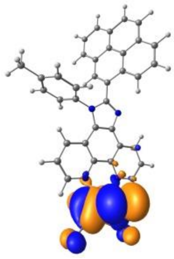 → 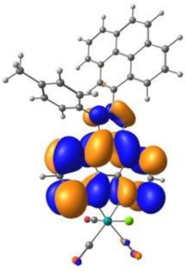

Osc. Strength 0.0605

H-1 → L+1 (65%)
H-1 → L+2 (16%)
H-3 → LUMO (13%)

NTO VIII:

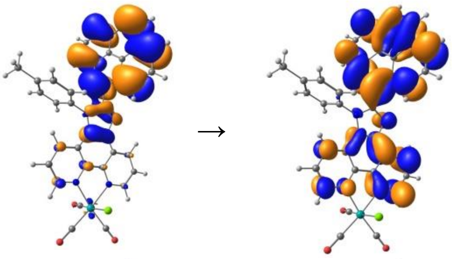

Osc. Strength 0.2720

HOMO → L+2 (90%)

NTO IX:

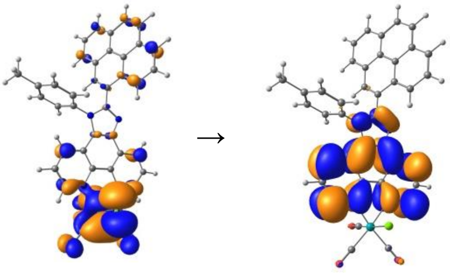

Osc. Strength 0.0121

H-2 → L+1 (72%)
H-2 → L+2 (16%)

Figure S16. Calculated NTOs of significant low-energy transitions for complex **2**.

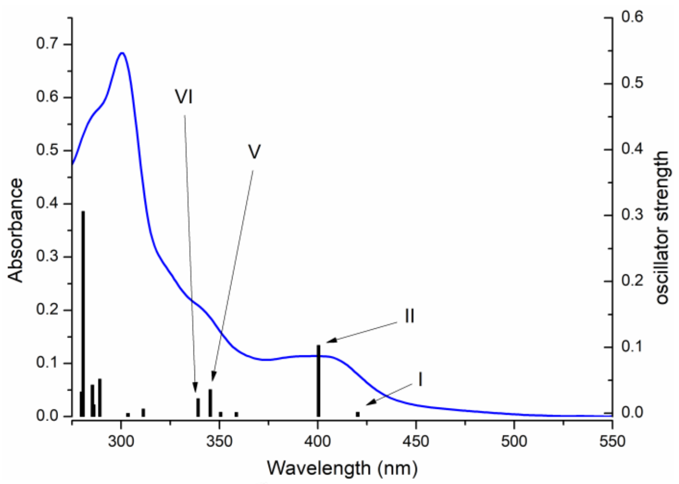

NTO I:

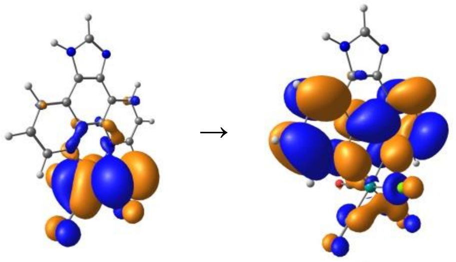

Osc. Strength 0.0019

HOMO → LUMO (99%)

NTO II:

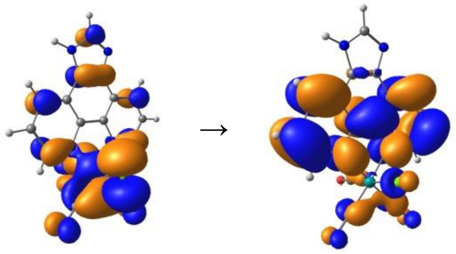

Osc. Strength 0.1033

H-1 → LUMO (98%)

NTO V:

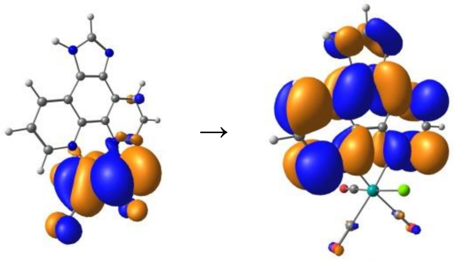

Osc. Strength 0.0362

HOMO → L+1 (69%)
H-2 → LUMO (26%)

NTO VI:

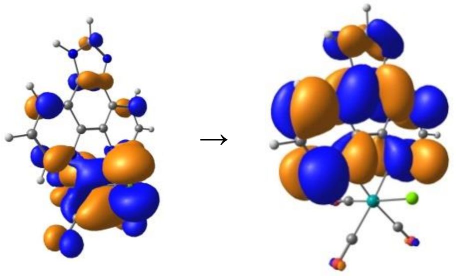

Osc. Strength 0.0225

H-1 → L+1 (93%)

Figure S17. Calculated NTOs of significant low-energy transitions for complex **3**.

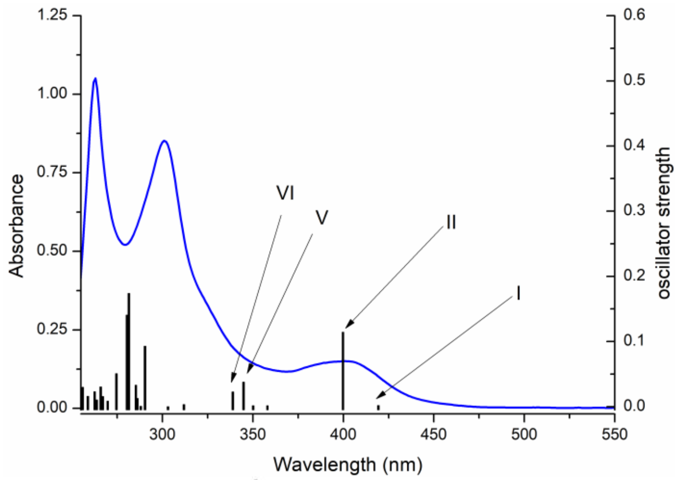

NTO I: 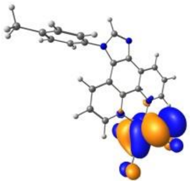 → 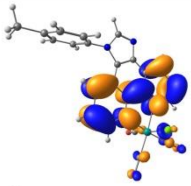

Osc. Strength 0.0019

HOMO → LUMO (99%)

NTO II: 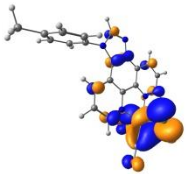 → 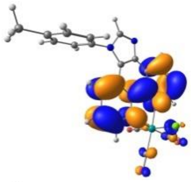

Osc. Strength 0.1145

H-1 → LUMO (98%)

NTO V: 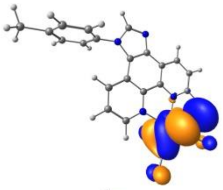 → 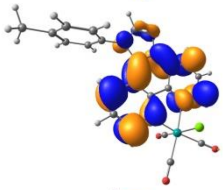

Osc. Strength 0.0378

HOMO → L+1 (70%)
H-2 → LUMO (26%)

NTO VI: 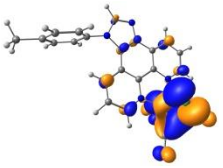 → 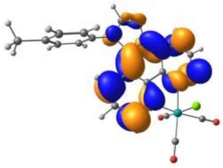

Osc. Strength 0.0229

H-1 → L+1 (93%)

Figure S18. Calculated NTOs of significant low-energy transitions for complex **4**.

Table S7. Calculated theoretical parameters of electron transitions for complex **1**.

**Medium**	**Experimental absorption λ; nm (10^4 ε; $M^{-1}cm^{-1}$)**	**Calculated transitions**				
		**Major contribution (%)**	**Character**	**E [eV]**	**λ [nm]**	**Oscillator strength**
DMSO	429 (1.12)	H-1 → LUMO (60%)<br>HOMO → LUMO (34%)	MLCT<br>ILCT	2.942	421.37	0.0349 S_1
		HOMO → LUMO (49%)<br>H-1 → LUMO (39%)<br>H-2 → LUMO (10%)	ILCT<br>MLCT<br>MLCT	2.952	419.97	0.0594 S_2
	374 (3.52)	H-2 → LUMO (80%)<br>HOMO → LUMO (12%)	MLCT<br>ILCT	3.130	396.08	0.0802 S_3
		HOMO → L+1 (93%)	IL	3.336	371.63	0.3318 S_4
		HOMO → L+2 (88%)	IL	3.538	350.44	0.2438 S_7
		H-1 → L+1 (40%)<br>H-3 → LUMO (27%)<br>H-1 → L+2 (23%)	MLCT<br>IL<br>MLCT	3.563	347.96	0.0589 S_8
	299 (3.64)	H-3 → L+1 (34%)<br>H-5 → L+1 (13%)<br>H-2 → L+1 (13%)<br>H-2 → L+2 (13%)	IL/ILCT<br>IL<br>MLCT<br>MLCT	3.921	316.18	0.2489 S_{11}
		H-3 → L+2 (41%)<br>H-3 → L+1 (14%)<br>H-2 → L+2 (10%)	IL<br>IL/ILCT<br>MLCT	4.159	298.11	0.1821S_{17}
	285 (4.88)	H-6 → LUMO (26%)<br>H-2 → L+5 (18%)	IL<br>MLCT	4.317	287.17	0.1372 S_{18}
		H-1 → L+5 (29%)<br>H-6 → LUMO (16%)<br>H-1 → L+6 (14%)<br>H-2 → L+5 (10%)	MLCT<br>IL<br>MLCT<br>MLCT	4.336	285.94	0.1096 S_{19}
		H-5 → L+1 (40%)<br>HOMO → L+4 (15%)	IL<br>IL	4.397	281.99	0.1283 S_{21}
		H-5 → L+2 (49%)<br>H-1 → L+3 (14%)	ILCT<br>MLCT	4.562	271.80	0.1929 S_{26}
		H-2 → L+7 (31%)<br>H-2 → L+3 (10%)	MLCT<br>MLCT	4.796	258.51	0.1468 S_{31}
		H-3 → L+3 (32%)<br>H-4 → L+7 (11%)	MLCT<br>MLCT/IL	4.857	255.29	0.1608 S_{32}
		H-7 → L+1 (45%)<br>H-7 → L+2 (15%)	IL<br>IL	4.905	252.78	0.1745 S_{35}

Table S8. Calculated theoretical parameters of electron transitions for complex **2**.

Medium	Experimental absorption λ; nm (10^4 ε; $M^{-1}cm^{-1}$)	Calculated transitions				
		**Major contribution (%)**	**Character**	**E [eV]**	**λ [nm]**	**Oscillator strength**
DMSO	407 (0.74)	H-1 → LUMO (99%)	MLCT	2.958	419.20	0.0017 S_1
		HOMO → LUMO (74%)<br>H-2 → LUMO (24%)	ILCT<br>MLCT	3.017	410.93	0.0858 S_2
		H-2 → LUMO (73%)<br>HOMO → LUMO (22%)	MLCT<br>ILCT	3.136	395.34	0.0681 S_3
	375 (1.36)	HOMO → L+1 (92%)	ILCT/IL	3.450	359.35	0.182 S_4
	347 (3.91)	H-1 → L+1 (65%)<br>H-1 → L+2 (16%)<br>H-3 → LUMO (13%)	MLCT<br>MLCT<br>IL	3.581	346.21	0.0605 S_7
		HOMO → L+2 (90%)	IL/ILCT	3.599	344.53	0.272 S_8
	331 (3.02)	H-2 → L+1 (72%)<br>H-2 → L+2 (16%)	MLCT<br>MLCT	3.659	338.87	0.0121 S_9
	301 (3.00)	H-3 → L+1 (48%)<br>H-7 → LUMO (14%)<br>H-6 → LUMO (10%),<br>H-3 → L+2 (10%)	IL<br>IL<br>ILCT<br>ILCT	3.952	313.68	0.0225 S_{11}
		H-2 → L+2 (33%)<br>HOMO → L+3 (23%)<br>H-3 → L+2 (11%)<br>H-2 → L+1 (10%)	MLCT<br>ILCT/IL<br>ILCT<br>MLCT	4.033	307.39	0.146 S_{12}
		H-5 → LUMO (73%)<br>HOMO → L+3 (12%)	ILCT<br>ILCT	4.048	306.26	0.0195 S_{13}
	280 (5.70)	H-6 → LUMO (79%)	ILCT	4.211	294.43	0.0912 S_{17}
		H-3 → L+2 (46%)<br>H-2 → L+2 (14%)	ILCT<br>MLCT	4.271	290.306	0.1378 S_{18}
		H-2 → L+7 (38%)<br>H-7 → LUMO (31%)	MLCT<br>IL	4.347	285.20	0.1226 S_{21}
		H-1 → L+3 (18%)<br>H-1 → L+9 (17%)<br>H-7 → LUMO (12%)	MLCT<br>MLCT/MC<br>IL	4.402	281.63	0.1212 S_{22}
	269 (4.91)	H-5 → L+1 (60%)<br>HOMO → L+4 (14%)<br>H-6 → L+1 (10%)	ILCT<br>IL/ILCT<br>ILCT	4.447	278.80	0.2261 S_{24}
		H-6 → L+1 (28%)<br>H-5 → L+2 (25%)<br>H-2 → L+3 (10%)	ILCT<br>ILCT<br>MLCT	4.543	272.90	0.1955 S_{27}
		H-2 → L+9 (38%)<br>H-4 → L+9 (12%)	MC/MLCT<br>MC/MLCT	4.802	258.17	0.0808 S_{37}
		H-2 → L+4 (21%)<br>H-3 → L+3 (17%)<br>H-5 → L+3 (14%)	MLCT<br>IL<br>IL/ILCT	4.930	251.48	0.2841 S_{41}

Table S9. Calculated theoretical parameters of electron transitions for complex **3**.

**Medium**	**Experimental absorption λ; nm (10^4 ε; $M^{-1}cm^{-1}$)**	**Calculated transitions**				
		**Major contribution (%)**	**Character**	**E [eV]**	**λ [nm]**	**Oscillator strength**
DMSO	396 (0.45)	HOMO → LUMO (99%)	MLCT	2.950	420.32	0.0019 S_1
		H-1 → LUMO (98%)	MLCT	3.097	400.34	0.1033 S_2
	340 (0.84)	H-3 → LUMO (90%)	MLCT	3.457	358.60	0.0015 S_3
		H-2 → LUMO (62%)<br>HOMO → L+1 (29%)	ILCT<br>MLCT	3.537	350.57	0.0018 S_4
		HOMO → L+1 (69%)<br>H-2 → LUMO (26%)	MLCT<br>ILCT	3.591	345.30	0.0362 S_5
		H-1 → L+1 (93%)	MLCT	3.656	339.11	0.0225 S_6
	301 (2.79)	HOMO → L+2 (50%)<br>HOMO → L+4 (13%)<br>H-4 → LUMO (12%)<br>H-1 → L+3 (12%)	MLCT<br>MC<br>ILCT<br>MLCT	4.086	303.45	0.052 S_9
	286 (2.30)	H-1 → L+3 (61%)<br>HOMO → L+3 (14%)	MLCT/MC<br>MLCT/MC	4.345	285.37	0.0429 S_{11}
		H-4 → LUMO (34%)<br>HOMO → L+4 (18%)<br>H-2 → L+1 (13%)<br>HOMO → L+2 (10%)	ILCT<br>MC<br>ILCT<br>MLCT	4.418	280.64	0.3066 S_{12}
		H-1 → L+2 (74%)<br>H-4 → L+1 (12%)	MLCT<br>ILCT	4.431	279.81	0.0327 S_{13}
		HOMO → L+4 (52%)<br>HOMO → L+2 (30%)	MC<br>MLCT	4.524	274.04	0.0654 S_{14}
	269 (4.91)	H-5 → LUMO (93%)	MLCT/ILCT	4.649	266.69	0.0200 S_{16}
		H-6 → LUMO (82%)	MLCT	4.706	263.43	0.0513 S_{17}
		H-1 → L+4 (48%)<br>H-3 → L+2 (22%)<br>HOMO → L+6 (11%)	MC<br>MLCT<br>MC/MLCT	4.849	255.71	0.0257 S_{19}

Table S10. Calculated theoretical parameters of electron transitions for complex **4**.

**Medium**	**Experimental absorption λ; nm (10^4 ε; $M^{-1}cm^{-1}$)**	**Calculated transitions**				
		**Major contribution (%)**	**Character**	**E [eV]**	**λ [nm]**	**Oscillator strength**
DMSO	402 (0.54)	HOMO → LUMO (99%)	MLCT	2.956	419.30	0.0019 S_1
		H-1 → LUMO (98%)	MLCT	3.101	399.79	0.1145 S_2
	321 (1.27)	H-3 → LUMO (92%)	MLCT	3.463	358.02	0.0015 S_3
		H-2 → LUMO (65%)<br>HOMO → L+1 (28%)	IL<br>MLCT	3.541	350.15	0.0015 S_4
		HOMO → L+1 (70%)<br>H-2 → LUMO (26%)	MLCT<br>IL	3.595	344.85	0.0378 S_5
		H-1 → L+1 (93%)	MLCT	3.658	338.95	0.0229 S_6
		H-2 → L+1 (66%)<br>H-5 → LUMO (24%)	IL<br>IL	3.976	311.87	0.0034 S_7
	302 (2.88)	H-4 → LUMO (43%)<br>HOMO → L+2 (29%)<br>H-2 → L+1 (11%)	ILCT<br>MLCT<br>IL	4.270	290.39	0.0928 S_9
		H-1 → L+2 (32%)<br>H-5 → LUMO (20%)<br>H-2 → L+1 (8%)<br>HOMO → L+2 (8%)	MLCT<br>IL<br>IL<br>MLCT	4.405	281.45	0.174 S_{13}
		H-5 → LUMO (18%)<br>H-1 → L+2 (44%)<br>HOMO → L+6 (7%)<br>H-5 → L+1 (7%)	IL<br>MLCT<br>MLCT<br>IL	4.421	280.47	0.1407 S_{14}
		HOMO → L+6 (54%)<br>HOMO → L+2 (28%)	MC/MLCT<br>MLCT	4.515	274.61	0.0509 S_{15}
	262 (4.20)	H-2 → L+2 (49%)<br>H-5 → L+1 (22%)<br>H-3 → L+2 (7%)	IL<br>IL<br>MLCT	5.058	245.13	0.8488 S_{25}

UV-Vis absorption

Table S11. Summary of UV-Vis properties of complexes **1-4** in DMSO. Concentration: 25 μM.

**complex**	**λ/nm (ε/ 10^4 $dm^3 \cdot mol^{-1} \cdot cm^{-1}$)**
**1**	429 (1.12); 374 (3.52); 299 (3.64); 285 (4.88)
**2**	407 (0.74); 375 (1.36); 347 (3.91); 331 (3.02); 301 (3.00); 280 (5.70); 269 (4.91)
**3**	396 (0.45); 340 (0.84); 322 (1.15); 301 (2.79); 286 (2.30); 260 (3.65)
**4**	402 (0.54); 321 (1.27); 302 (2.88); 262 (4.20)

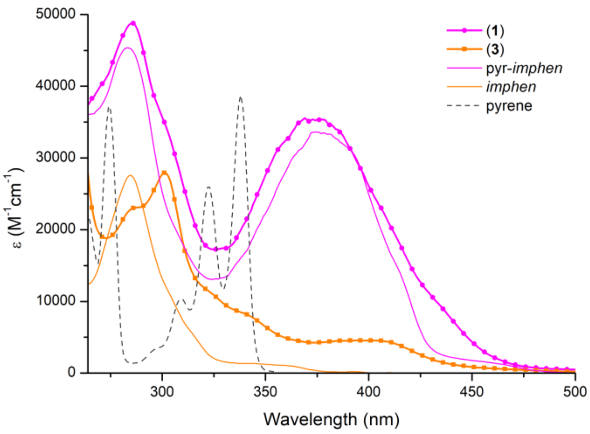

1

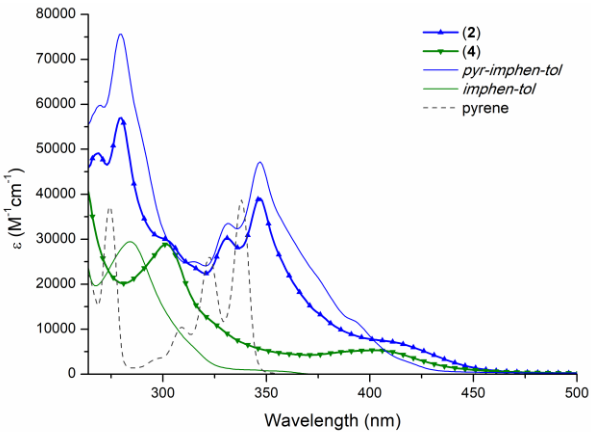

2

Figure S19. Comparison of UV-Vis spectra in DMSO of compounds **1** and **2** with spectra of their respective ligands and model complexes 3 and 4 as well as pyrene building block. Concentration: 25 µM.

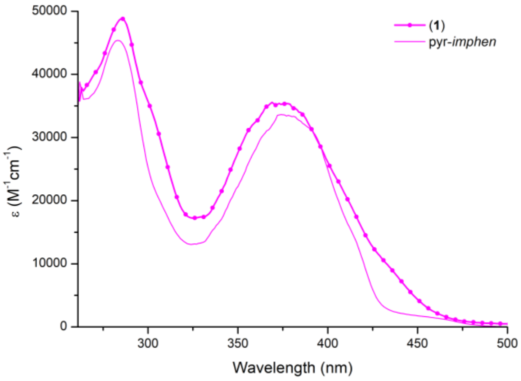

1

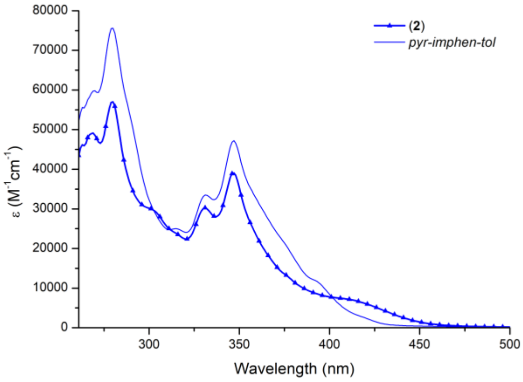

2

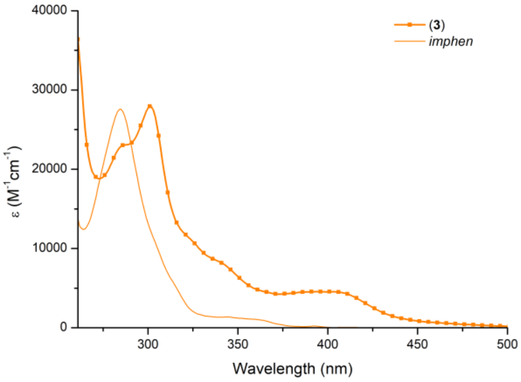

3

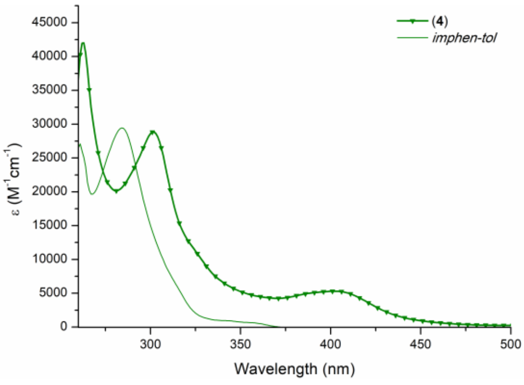

4

Figure S20. UV-Vis spectra of complexes **1-4** and their respective ligands in DMSO. Concentration: 25 µM.

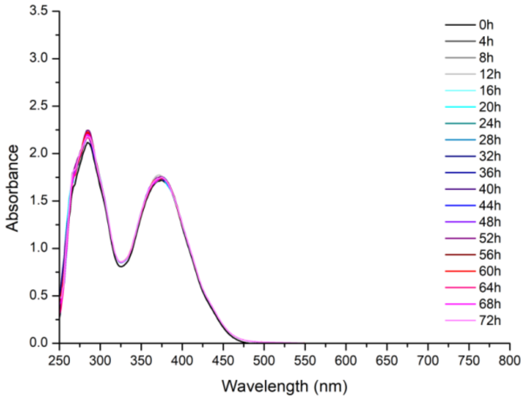

1

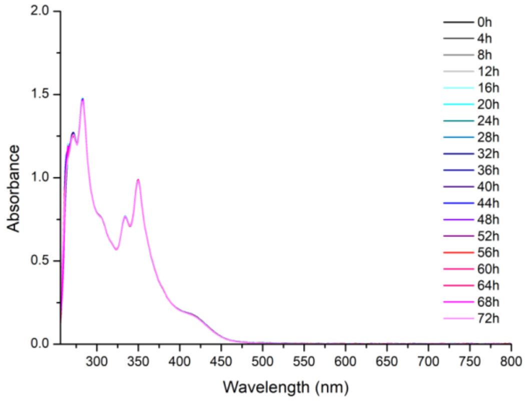

2

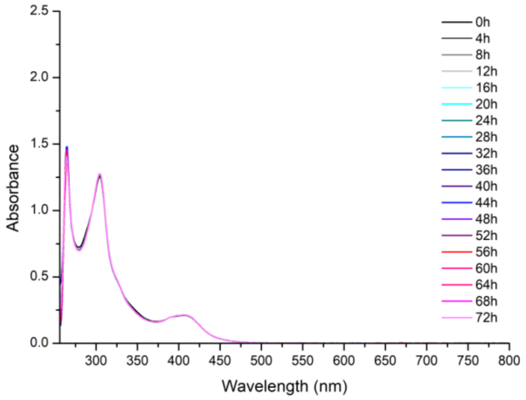

3

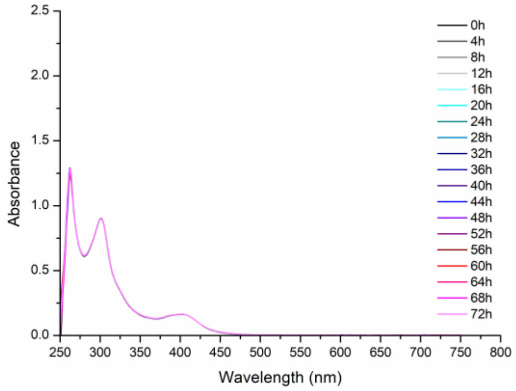

4

Figure S21. UV-Vis stability of complexes **1-4** in DMSO. Spectra recorded every 4h for 72h.

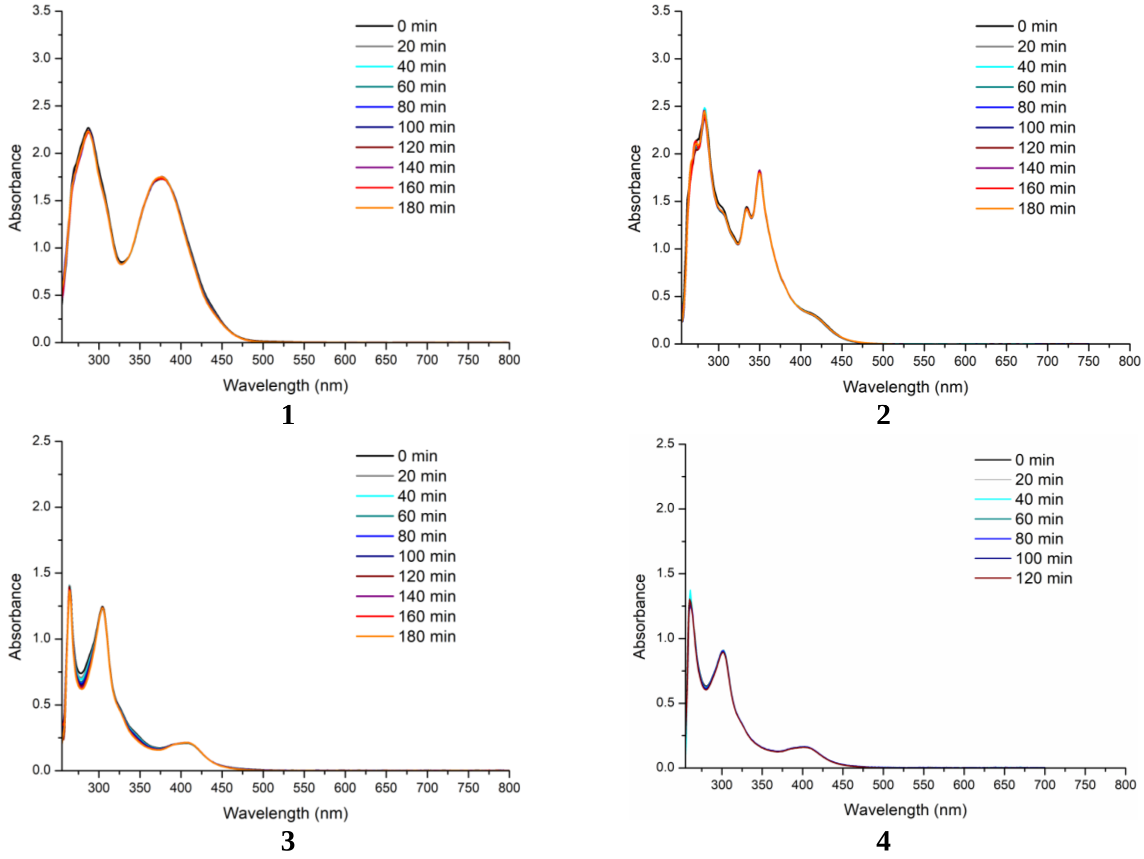

Figure S22. UV-Vis photostability of complexes **1-4** in DMSO upon irradiation with wavelength 420 nm. Spectra were recorded every 20 min.

Emission spectroscopy

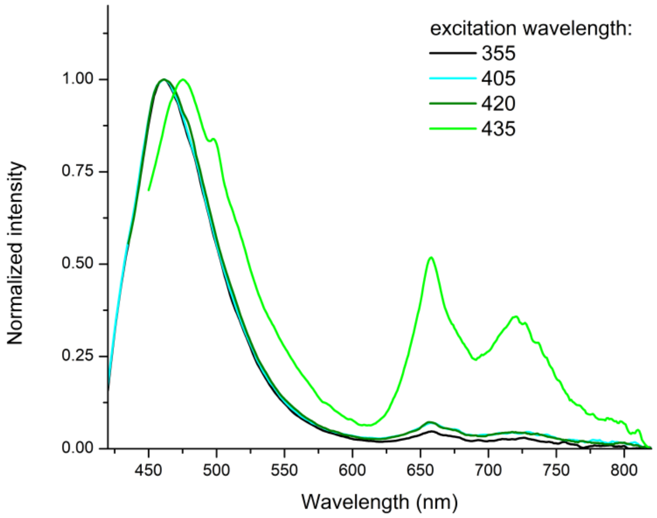

1

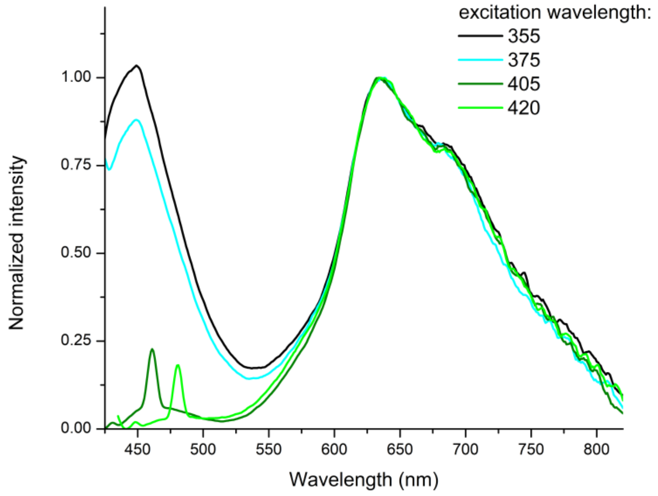

2

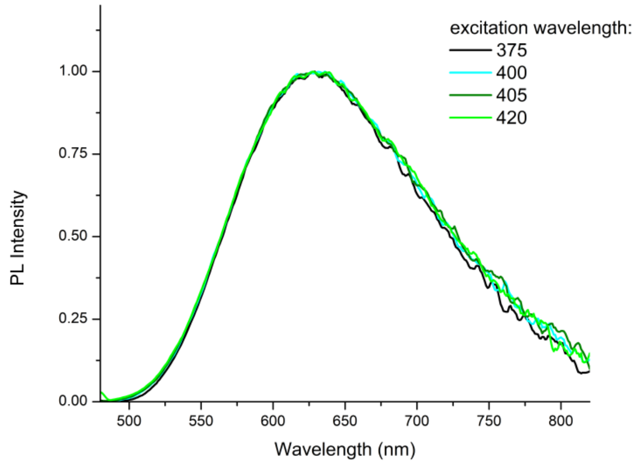

3

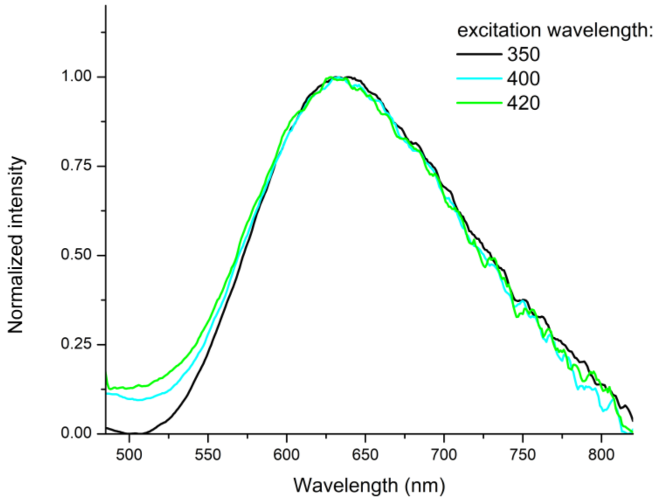

4

Figure S23. Emission spectra recorded upon different excitation wavelengths (nm) for **1–4** in DMSO.

1
RT

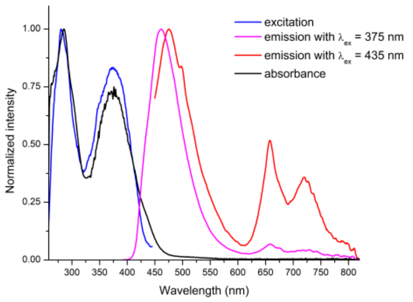

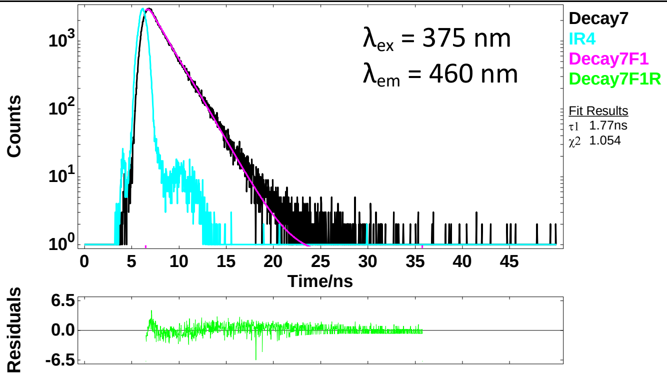

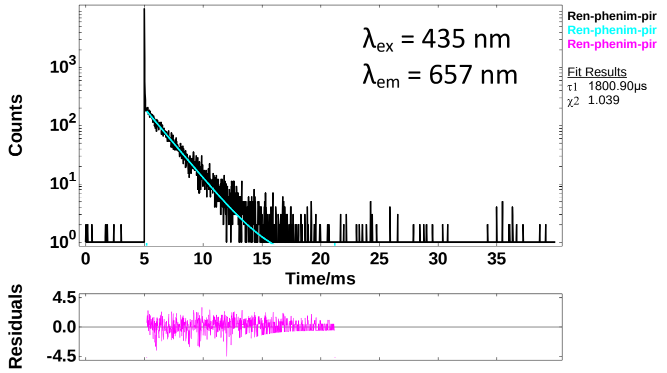

1
77K

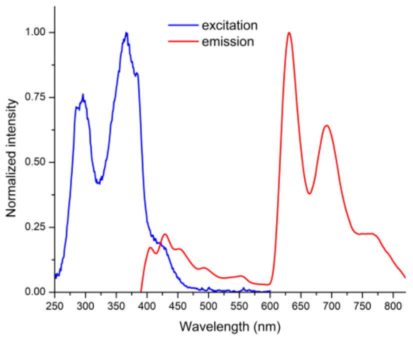

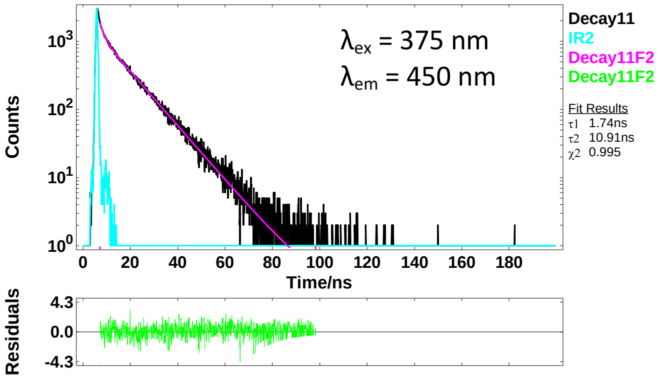

Fit Parameters

Fit = A + B1.exp(-t/T1) + B2.exp(-t/T2)

	Value	Std Dev		Value	Std Dev	Rel %
**T1**	1.744E-9	8.238E-11	**B1**	5.392E-2	3.817E-2	15.46
**T2**	1.091E-8	5.308E-11	**B2**	4.711E-2	5.390E-3	84.54
**Chisq**	9.950E-1		**A**	1.820E-1		
**Shift**	2.561E-11					

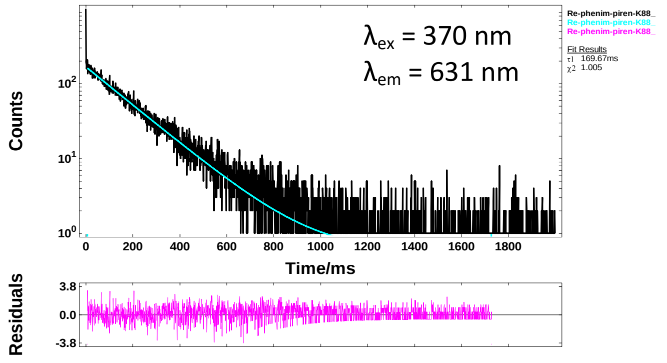

2
RT

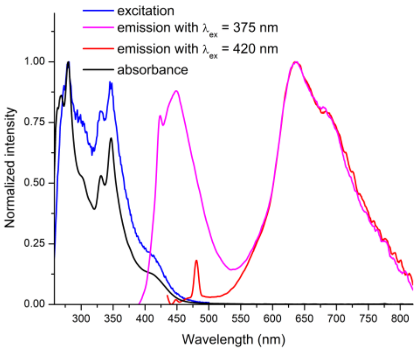

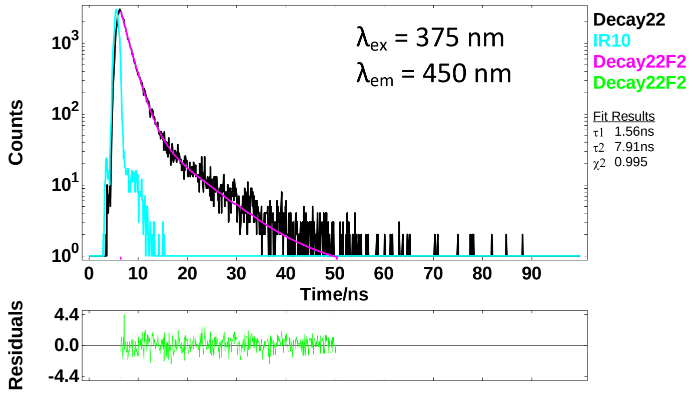

Fit Parameters

Fit = A + B1.exp(-t/T1) + B2.exp(-t/T2)

	Value	Std Dev		Value	Std Dev	Rel %
**T1**	1.557E-9	1.515E-11	**B1**	1.526E-1	7.599E-3	91.05
**T2**	7.907E-9	3.910E-10	**B2**	2.955E-3	2.588E-4	8.95
**Chisq**	9.948E-1		**A**	6.240E-1		
**Shift**	7.992E-11					

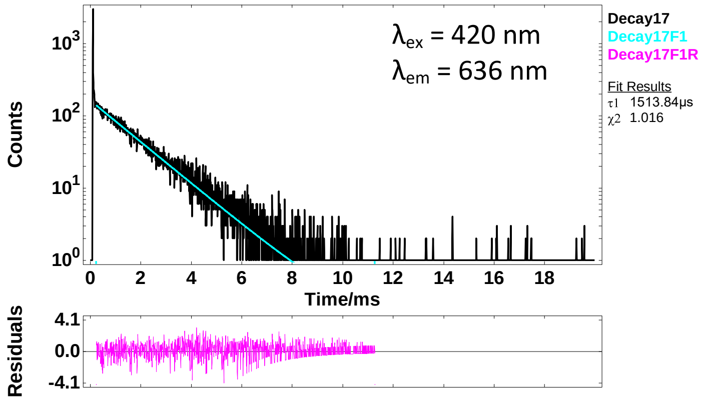

2
77K

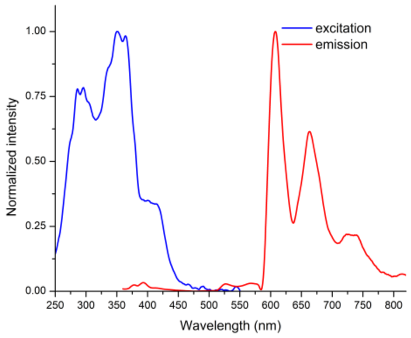

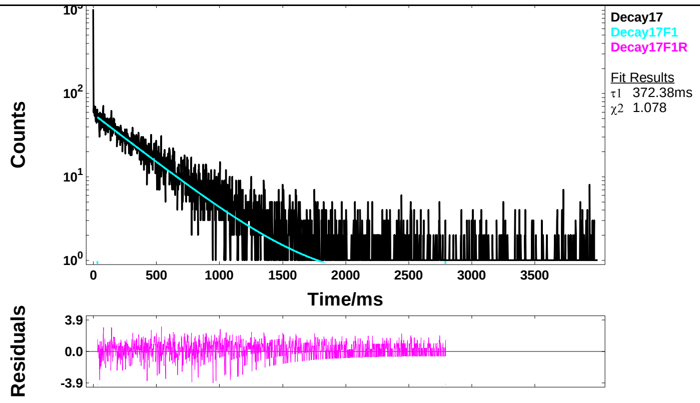

3 RT

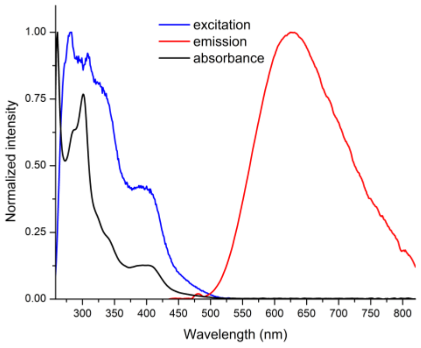

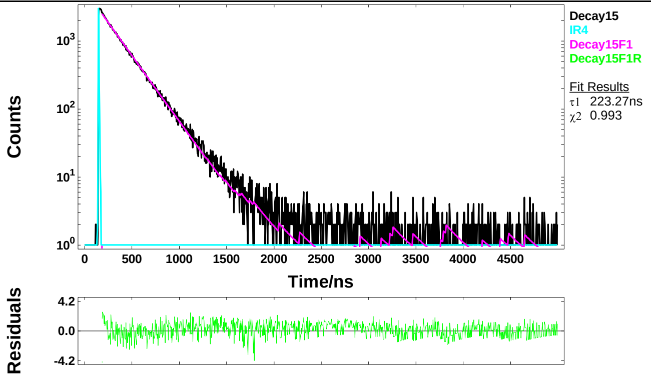

3 77K

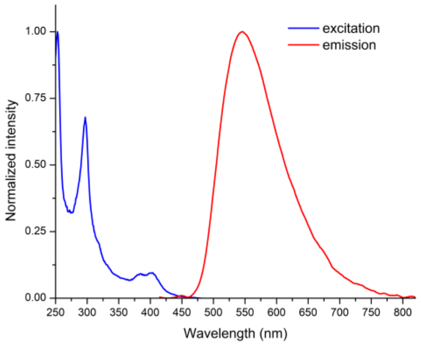

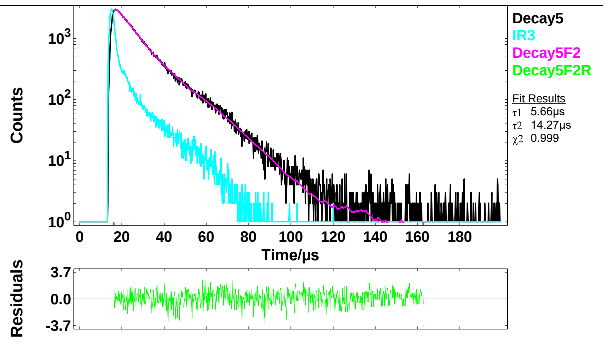

Fit Parameters

Fit = A + B1.exp(-t/T1) + B2.exp(-t/T2)

	Value	Std Dev		Value	Std Dev	Rel %
**T1**	5.664E-6	1.177E-7	**B1**	9.615E-2	1.238E-3	75.64
**T2**	1.427E-5	4.904E-7	**B2**	1.229E-2	1.451E-3	24.36
**Chisq**	9.990E-1		**A**	2.930E-1		
**Shift**	2.134E-7					

4 RT

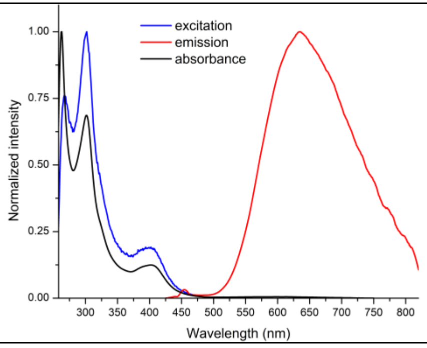

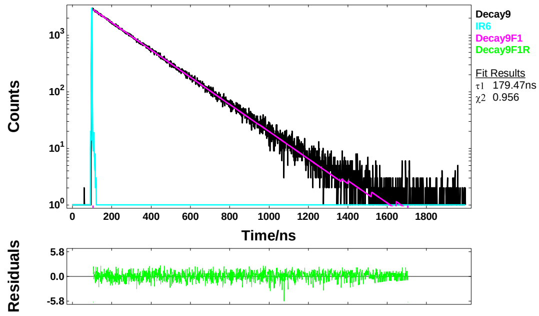

4 77K

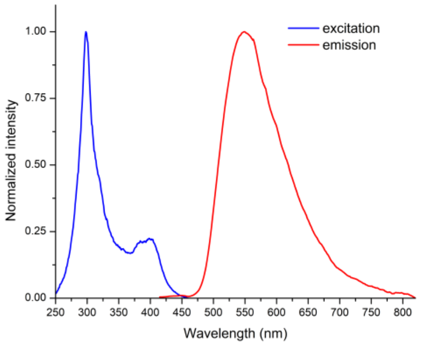

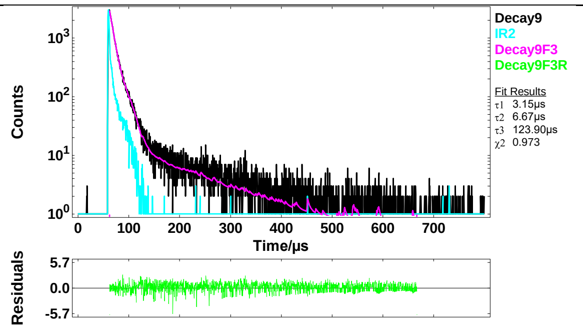

Fit Parameters

Fit = A + B1.exp(-t/T1) + B2.exp(-t/T2) + B3.exp(-t/T3)

	Value	Std Dev		Value	Std Dev	Rel %
**T1**	3.149E-6	6.613E-7	**B1**	9.580E-2	1.980E-2	25.49
**T2**	6.666E-6	3.525E-7	**B2**	1.231E-1	2.444E-2	69.33
**T3**	1.239E-4	6.612E-6	**B3**	4.951E-4	2.717E-5	5.18
**Chisq**	9.731E-1		**A**	3.840E-1		
**Shift**	3.202E-7					

Figure S24. Summary of luminescence properties of complexes **1–4**.

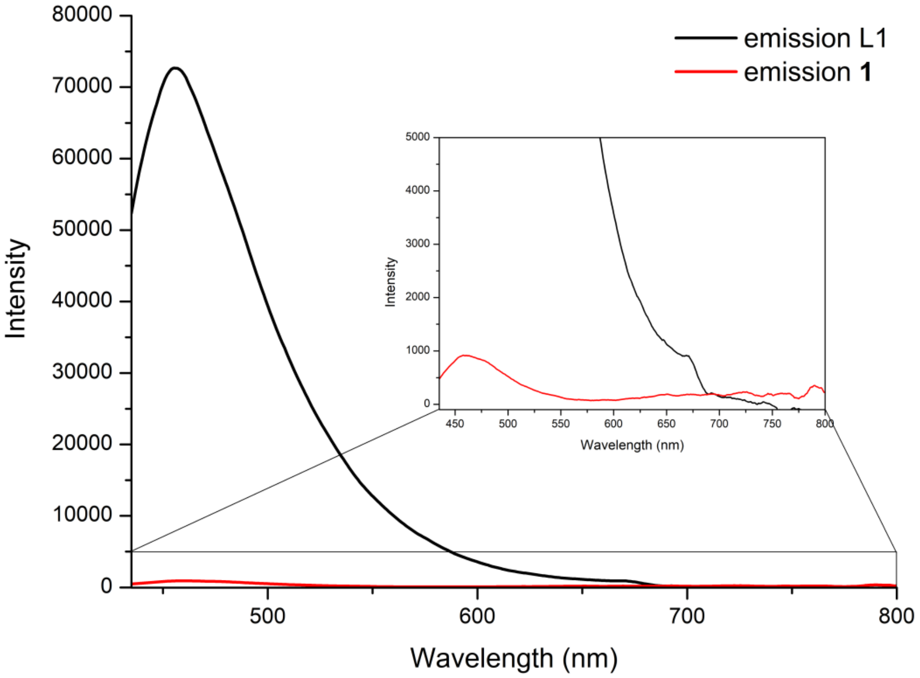

1

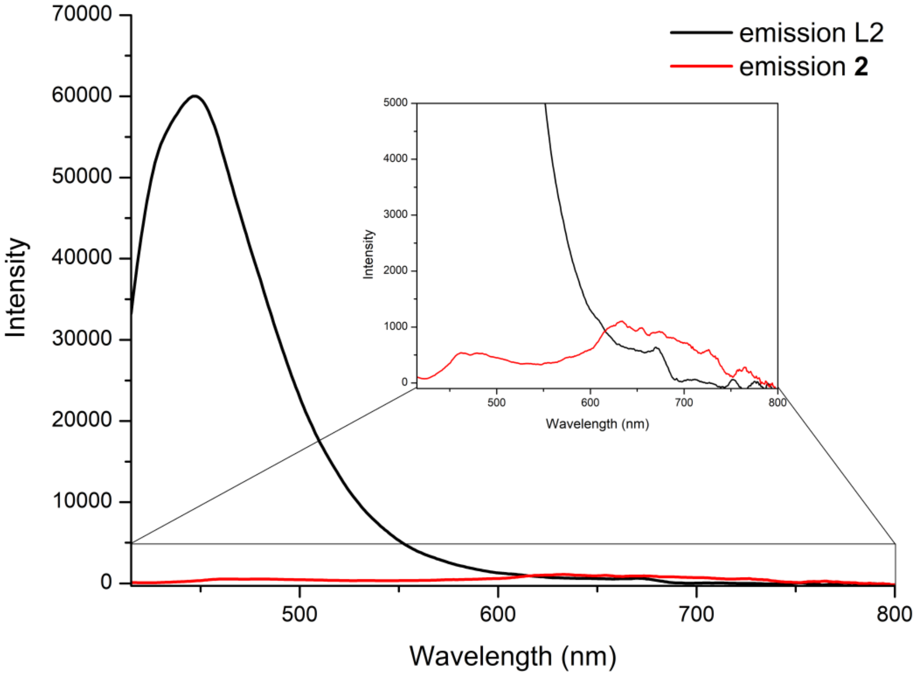

2

Figure S25. Comparison of emission intensity of Re(I) complexes **1** and **2** with their respective ligands (L1 and L2) with identical measurement conditions (DMSO, concentration of compounds and ligands: 50 μM, excitation: 405 nm).

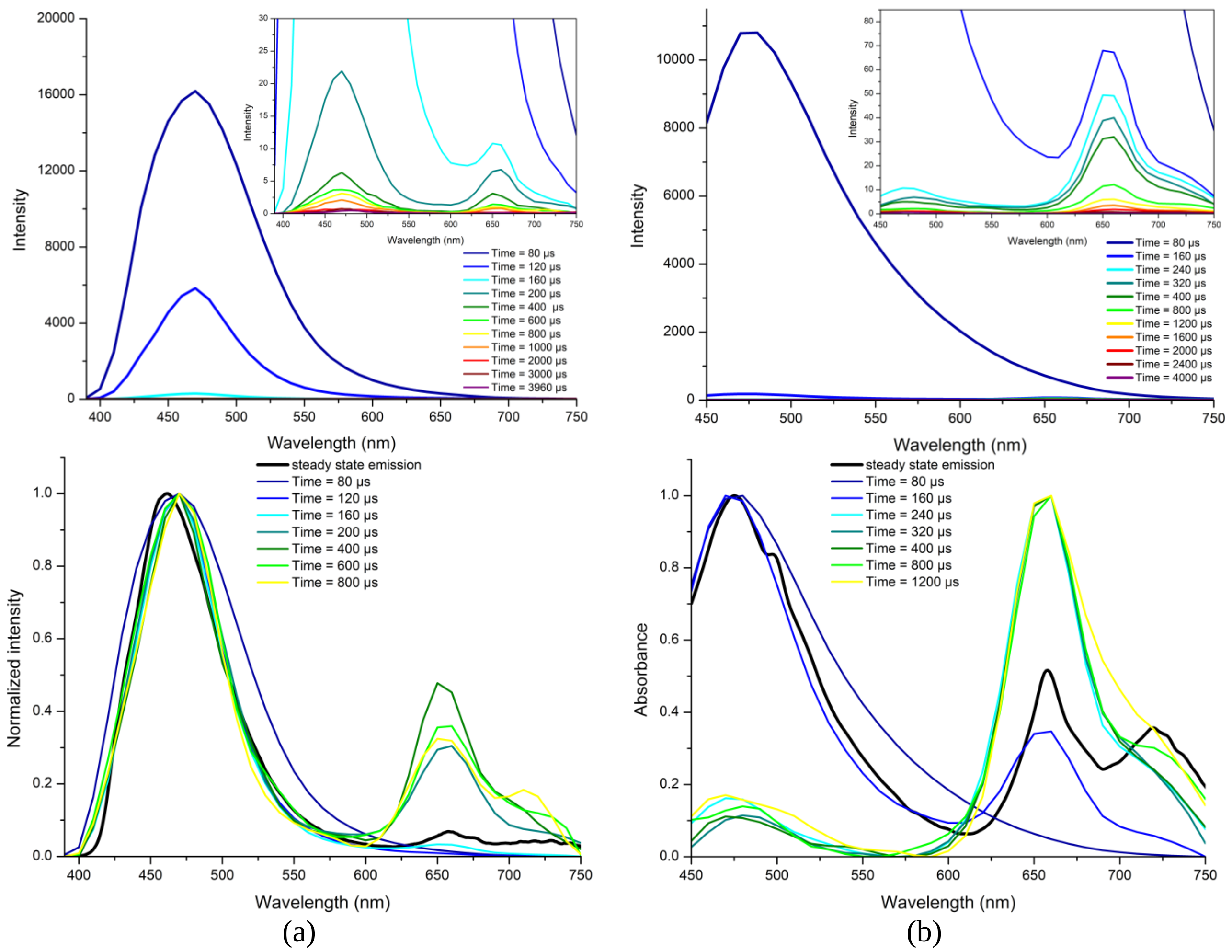

Figure S26. Time-resolved emission spectra (TRES) of **1** along with low intensity close-up in inset (upper panel) and selected normalized time-resolved spectra in comparison with steady state emission (lower panel). Measurements in RT DMSO (concentration 50 μM), excitation wavelength 355 nm (a) and 435 nm (b).

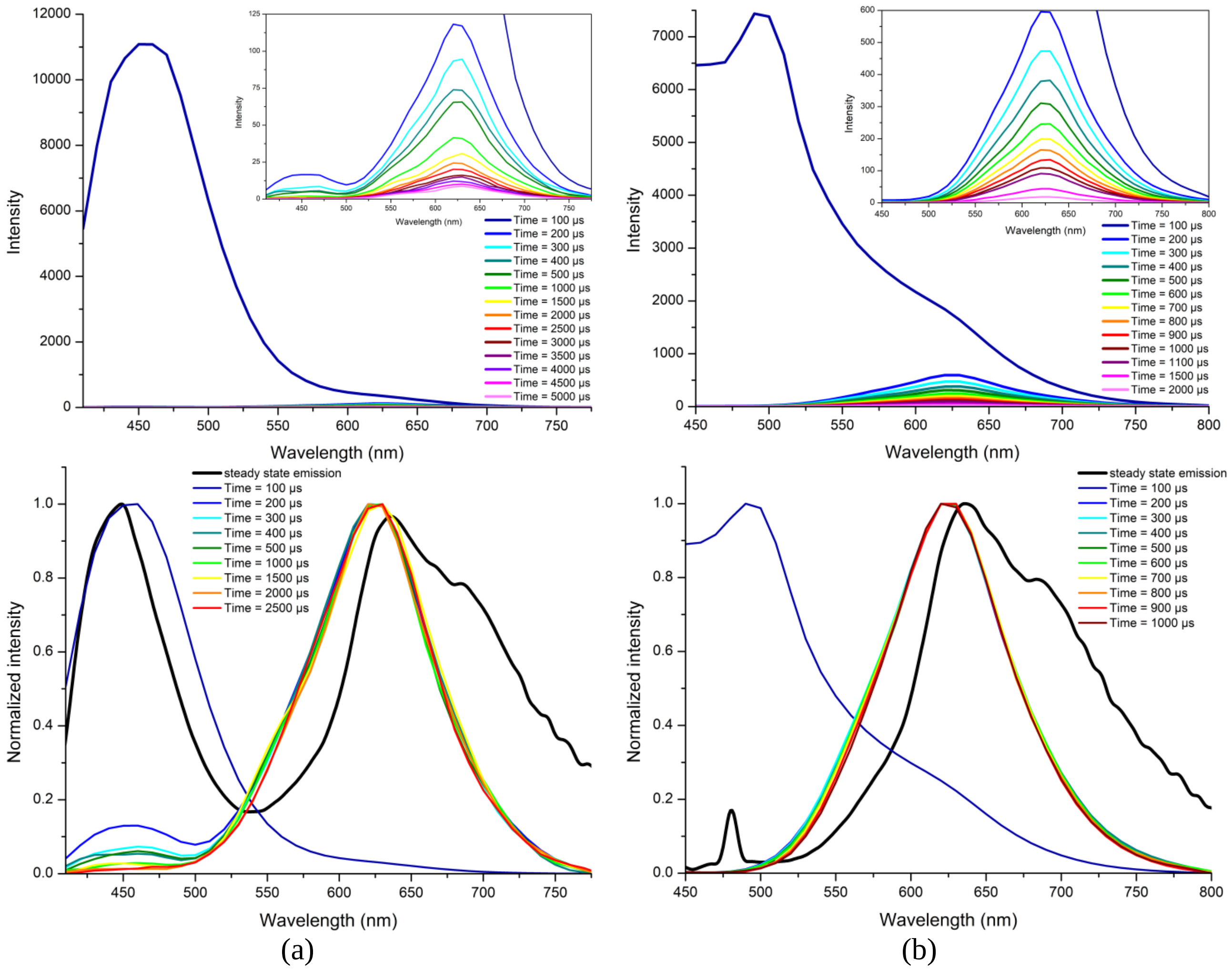

Figure S27. Time-resolved emission spectra (TRES) of **2** along with low intensity close-up in inset (upper panel) and selected normalized time-resolved spectra in comparison with steady state emission (lower panel). Measurements in RT DMSO (concentration 50 μM), excitation wavelength 355 nm (a) and 435 nm (b).

1

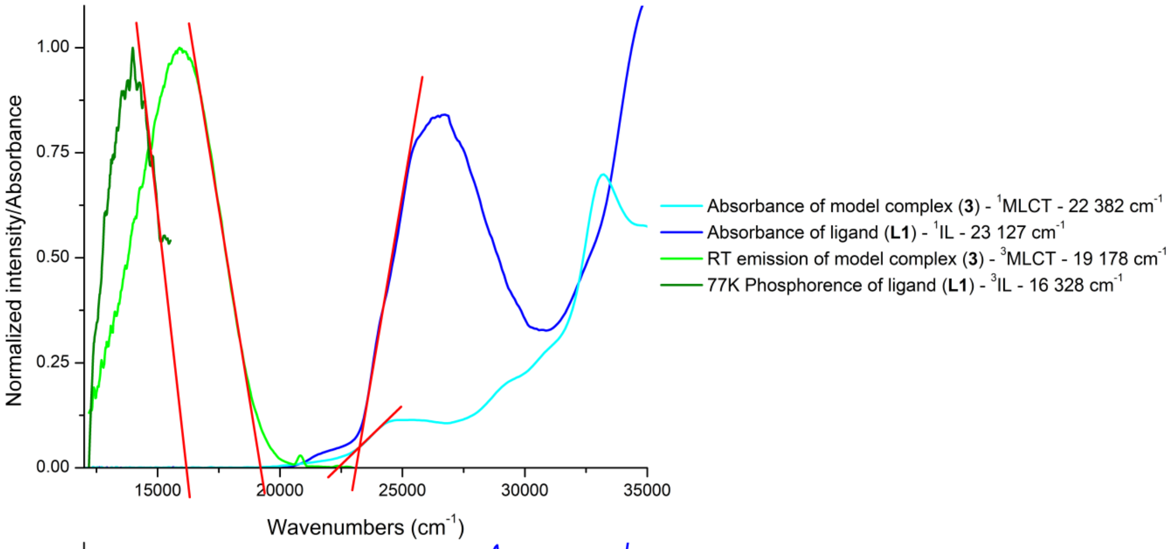

2

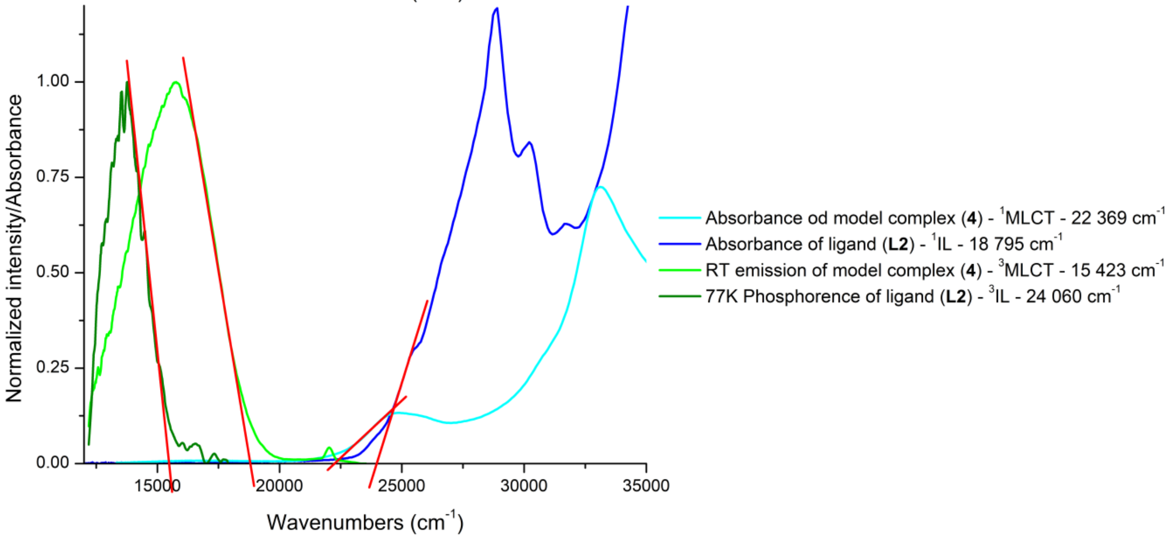

Figure S28. Method of calculating energies of ^{1}MLCT, ^{3}MLCT, ^{1}IL and ^{3}IL states of **1** and **2** and the obtained values. Red lines indicate tangents used for determination of the intersection with the X-axis.

Femtosecond transient absorption

355 nm	420 nm

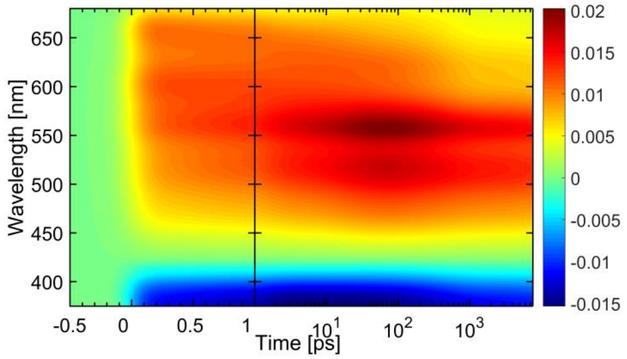

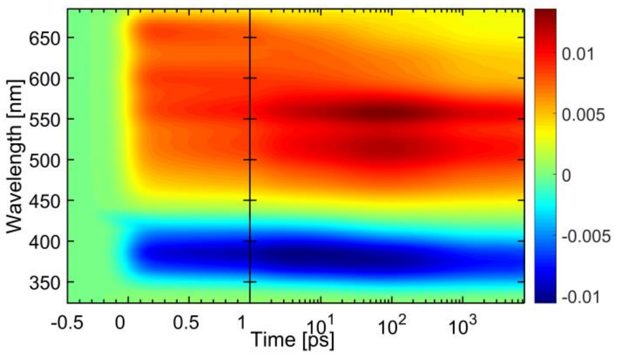

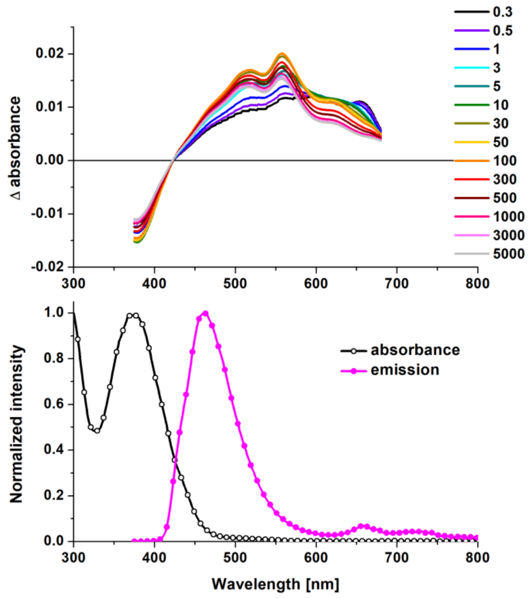

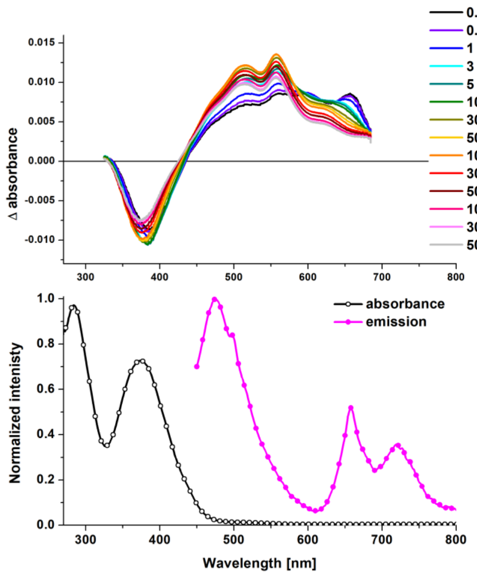

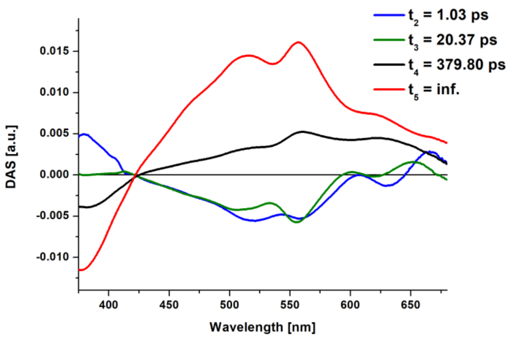

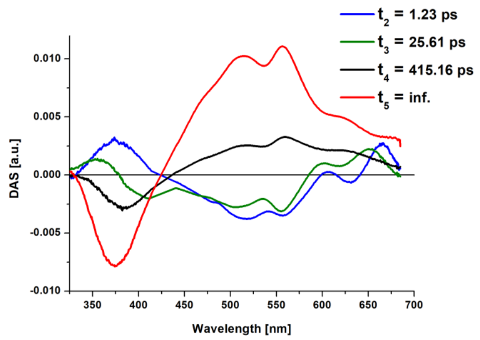

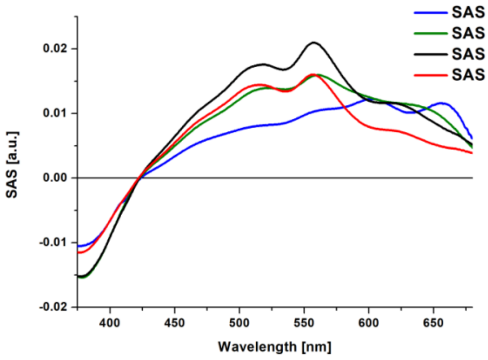

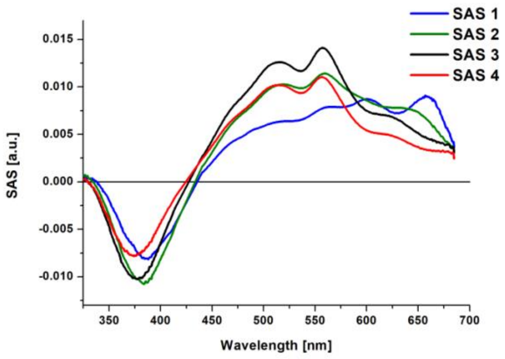

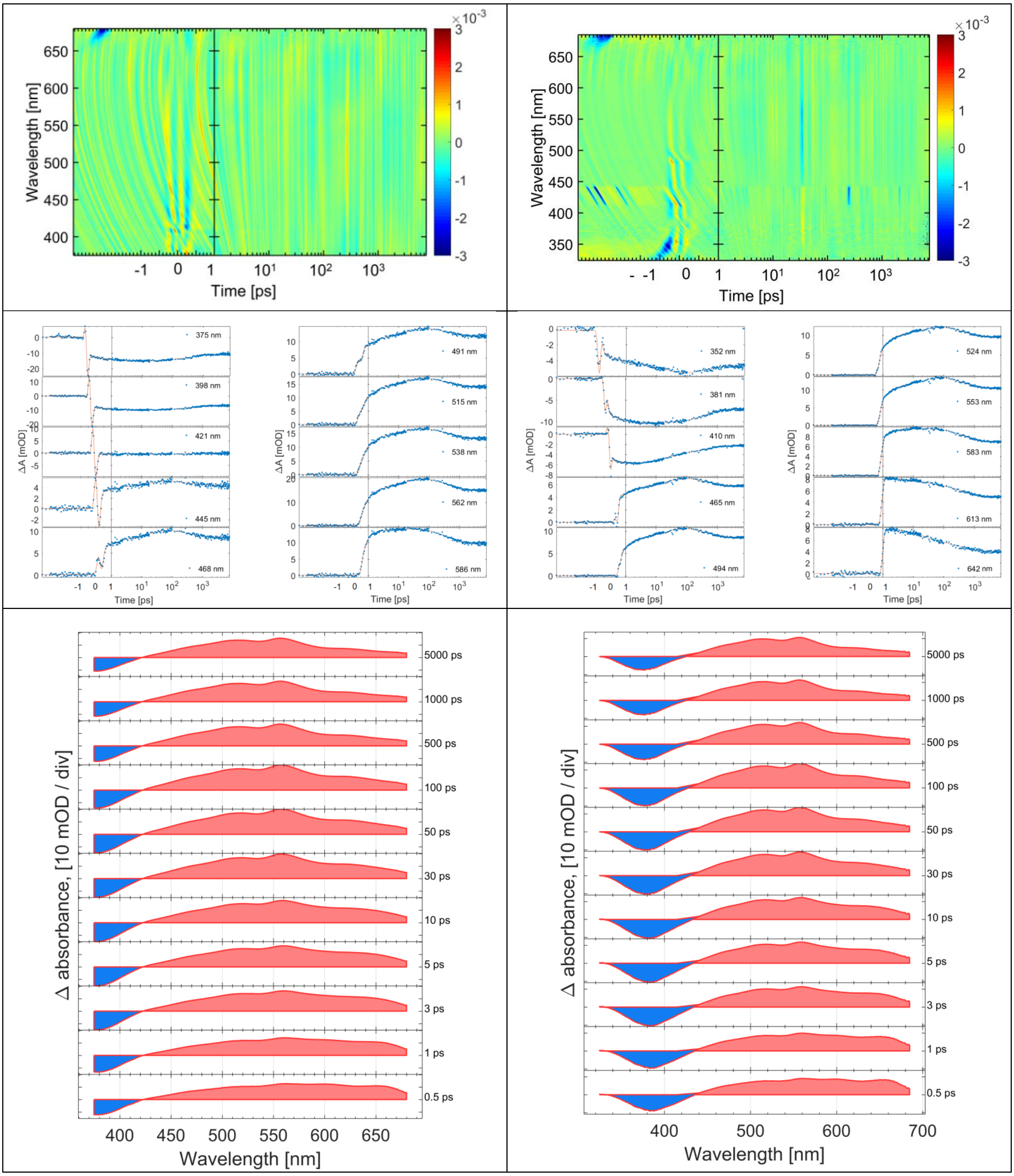

Figure S29. Summary of fs-TA measurements of complex **1** in two pump wavelengths: 355 nm and 420 nm.

355 nm	420 nm

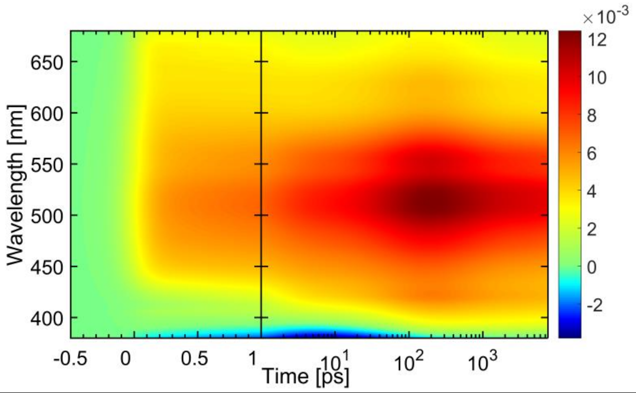

×10-3
Wavelength [nm]
Time [ps]

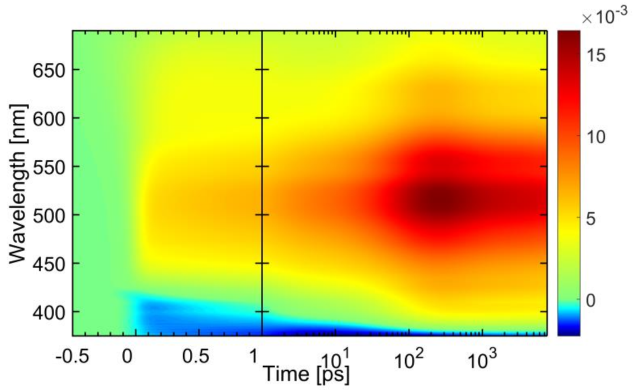

×10-3
Wavelength [nm]
Time [ps]

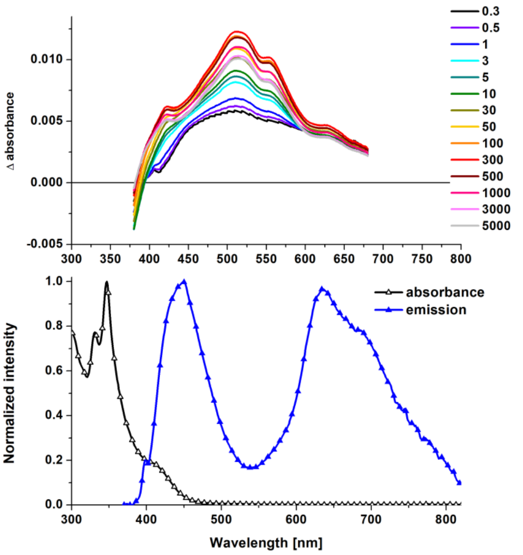

Δ absorbance
0.3
0.5
1
3
5
10
30
50
100
300
500
1000
3000
5000
Normalized intensity
absorbance
emission
Wavelength [nm]

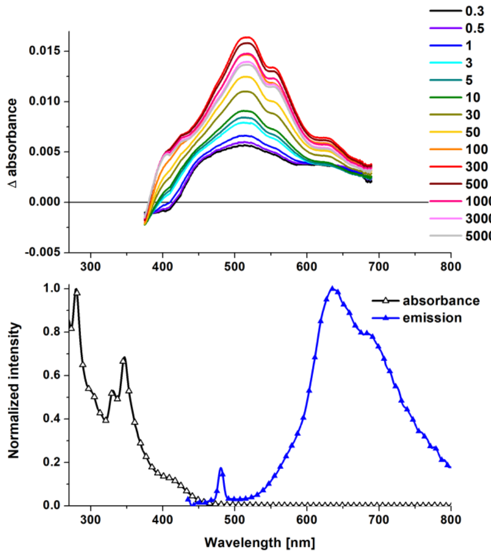

Δ absorbance
0.3
0.5
1
3
5
10
30
50
100
300
500
1000
3000
5000
Normalized intensity
absorbance
emission
Wavelength [nm]

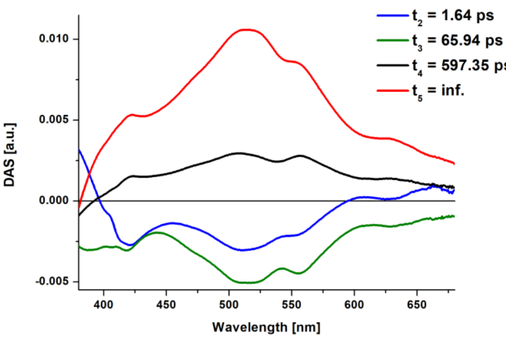

DAS [a.u.]
t2 = 1.64 ps
t3 = 65.94 ps
t4 = 597.35 ps
t5 = inf.
Wavelength [nm]

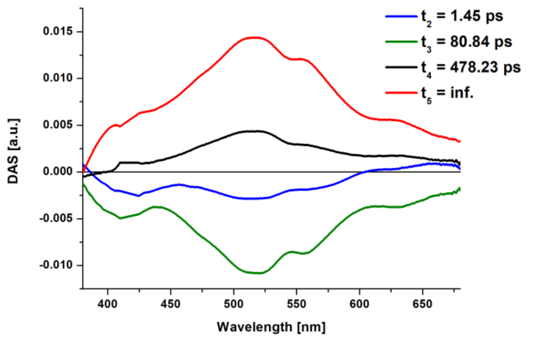

DAS [a.u.]
t2 = 1.45 ps
t3 = 80.84 ps
t4 = 478.23 ps
t5 = inf.
Wavelength [nm]

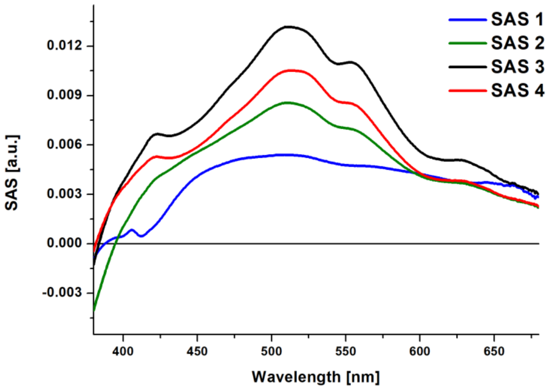

SAS [a.u.]
SAS 1
SAS 2
SAS 3
SAS 4
Wavelength [nm]

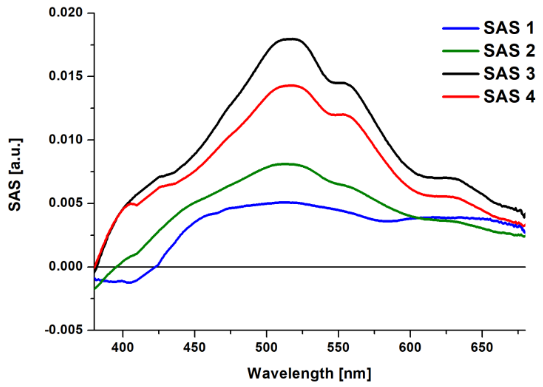

SAS [a.u.]
SAS 1
SAS 2
SAS 3
SAS 4
Wavelength [nm]

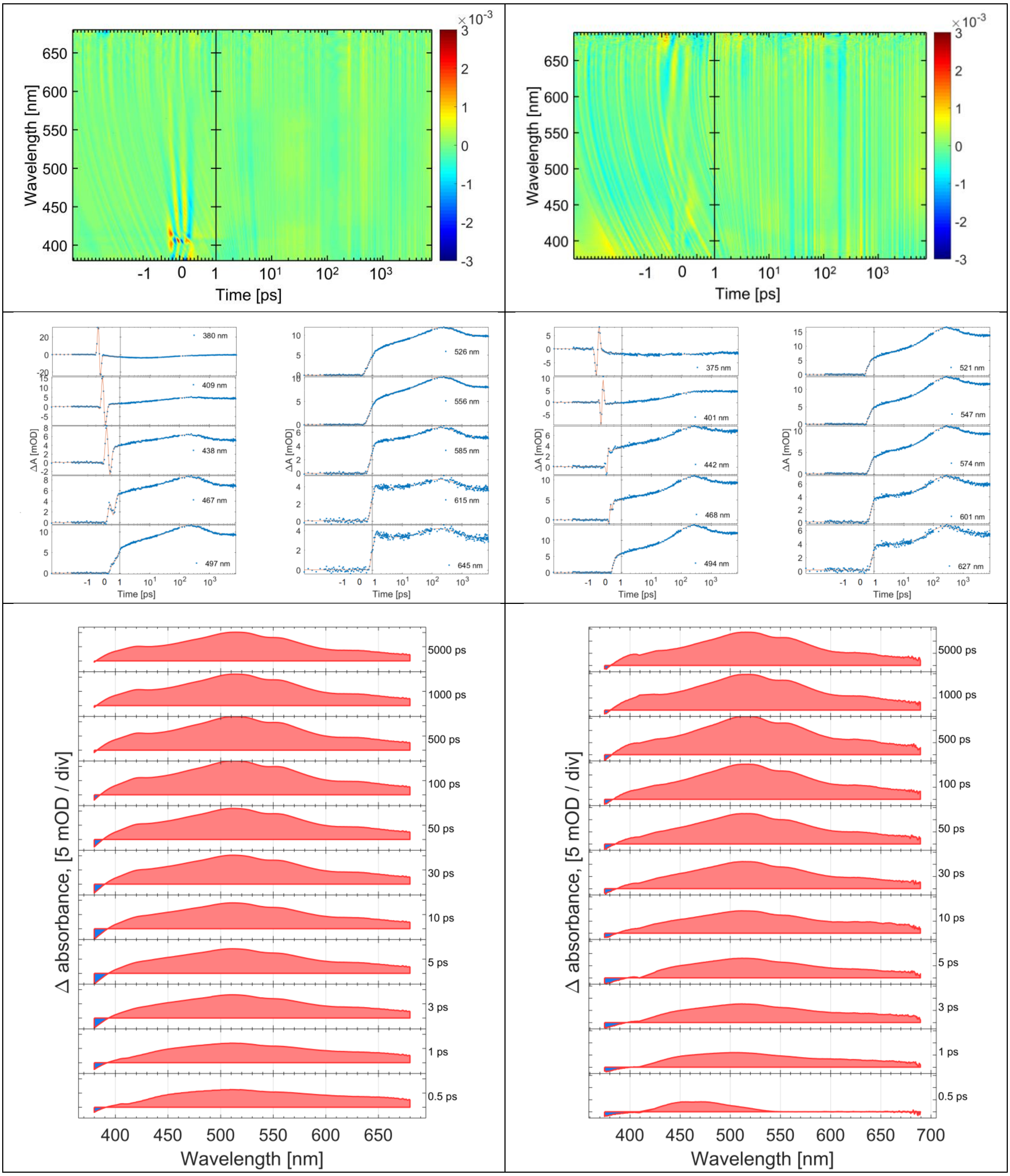

Figure S30. Summary of fs-TA measurements of complex **2** in two pump wavelengths: 355 nm and 420 nm.

**3**<br>**355 nm**	**4**<br>**355 nm**
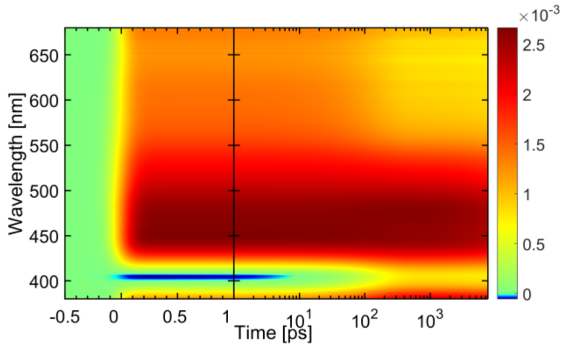	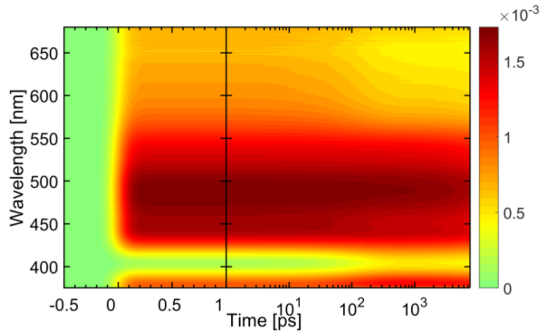
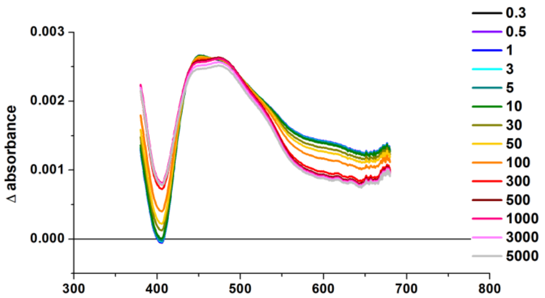<br>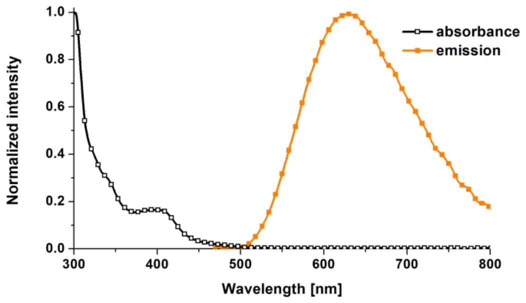	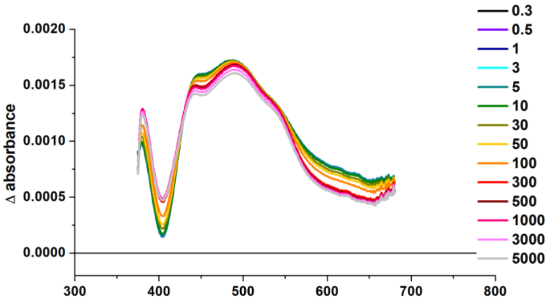<br>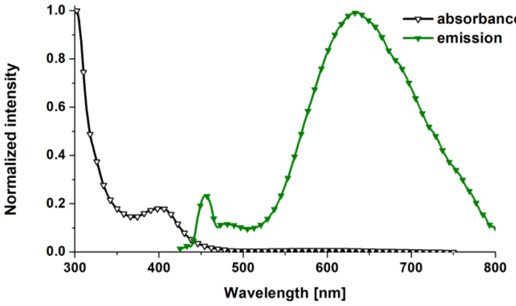
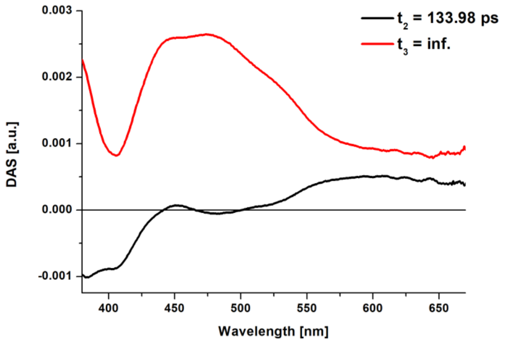	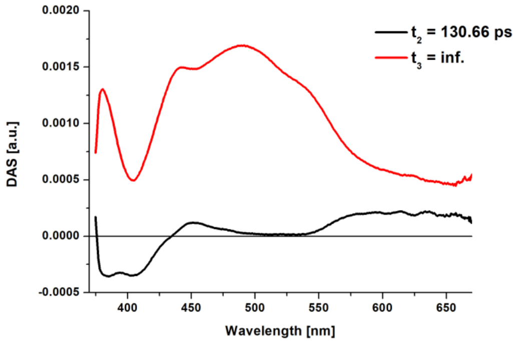
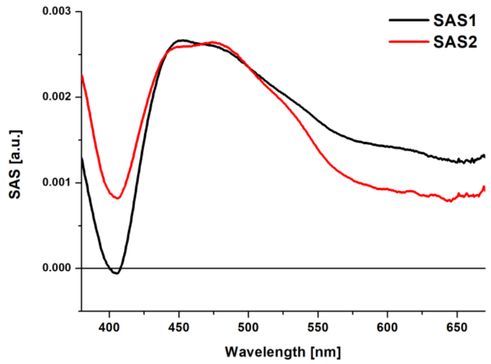	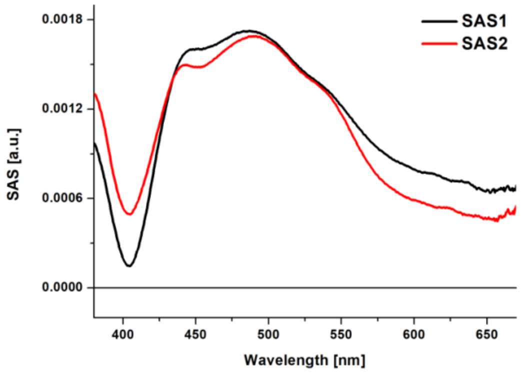

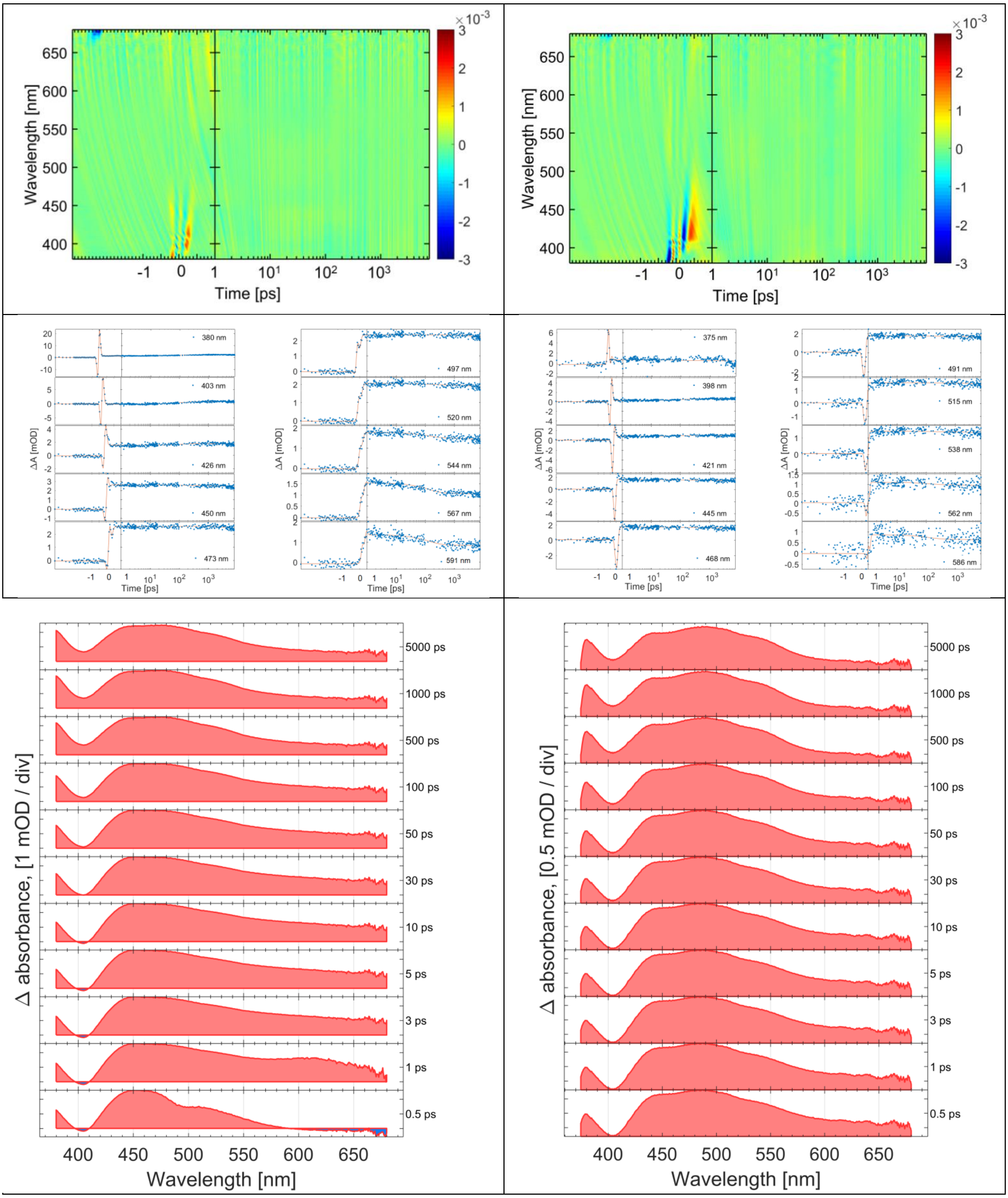

Figure S31. Summary of fs-TA measurements of complexes **3** and **4**.

L1 (pyr-imphen) 355 nm	L2 (pyr-tol-imphen) 355 nm
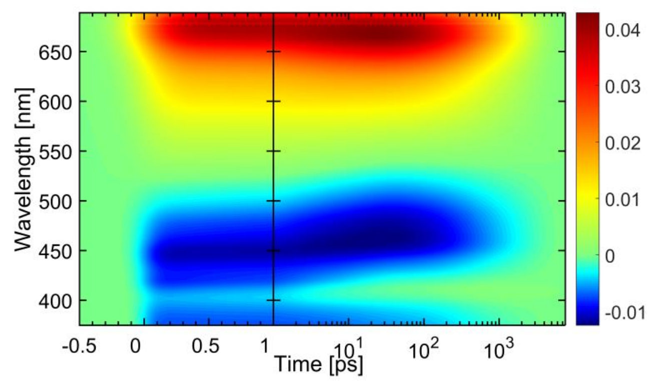	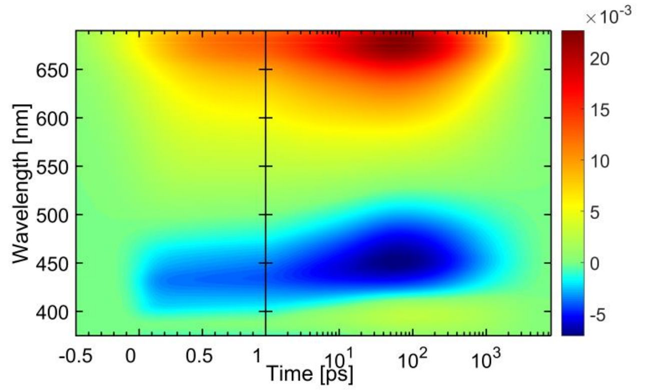
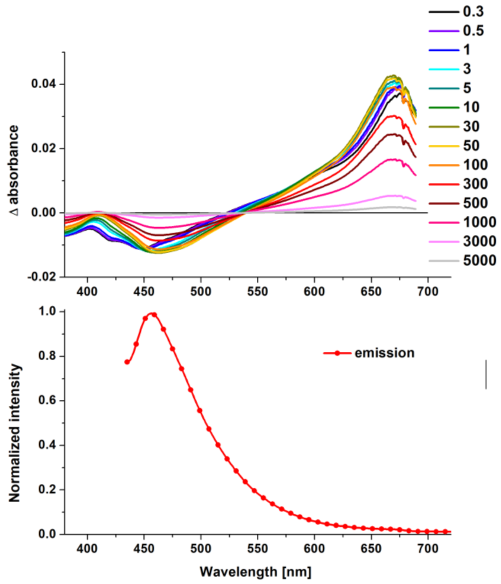	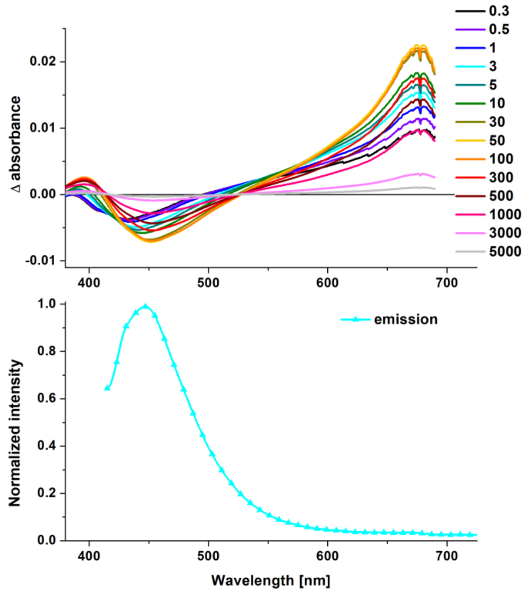
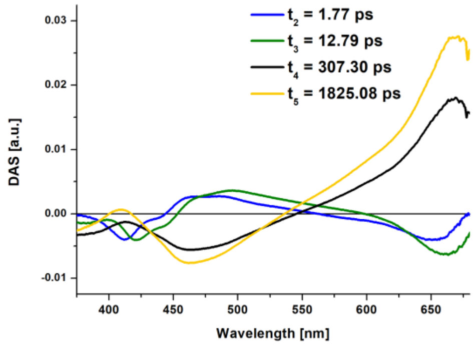	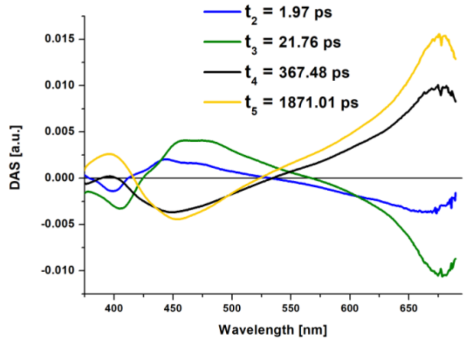
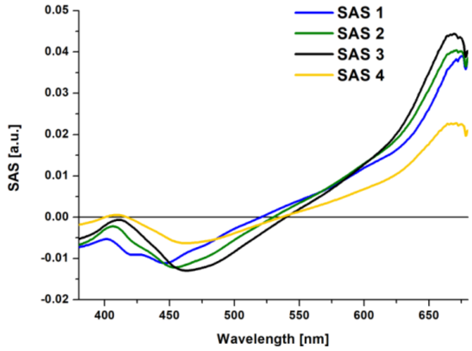	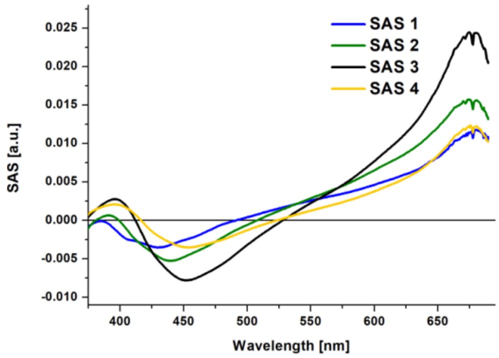

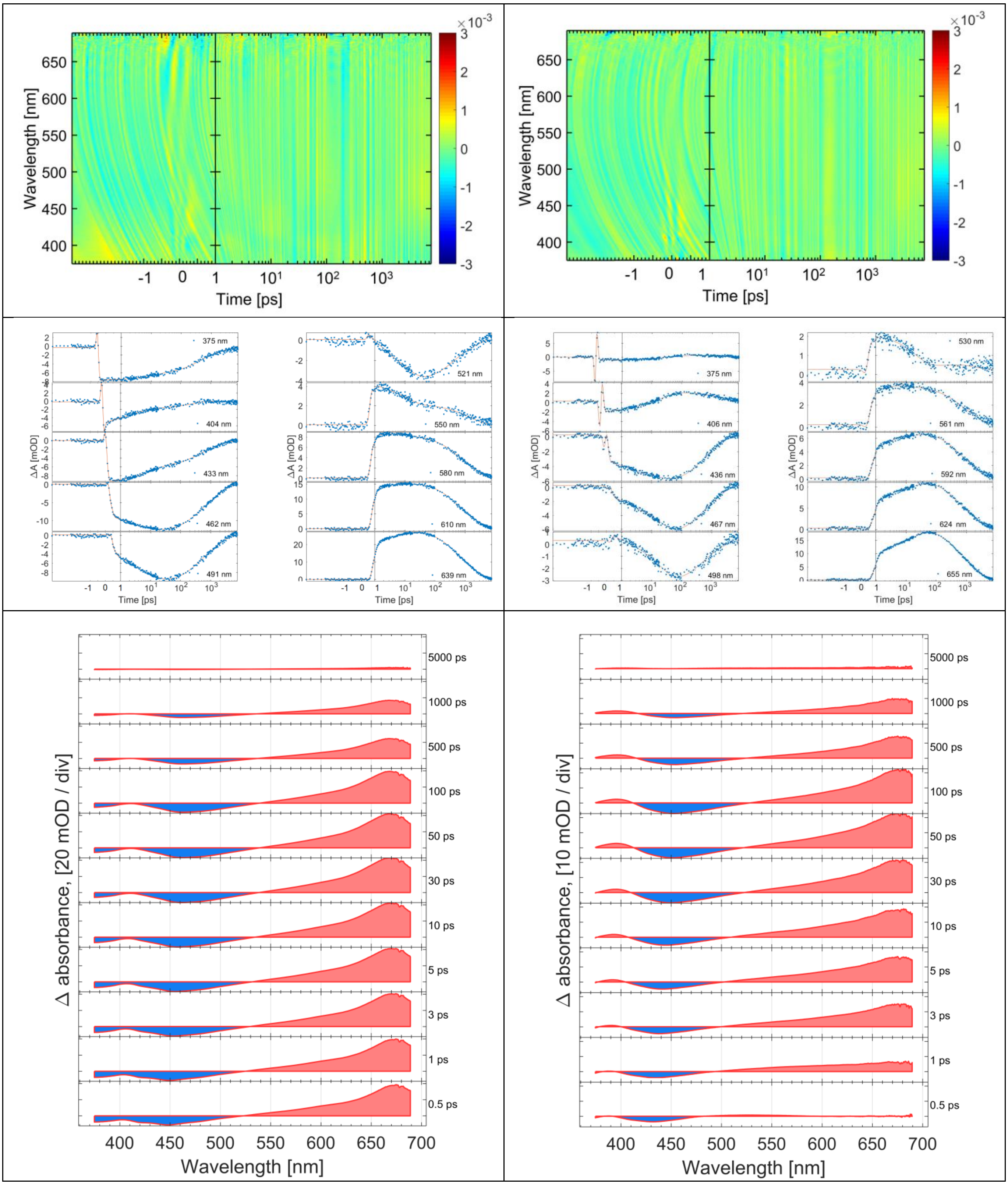

Figure S32. Summary of fs-TA measurements of ligands L1 and L2.

Singlet oxygen generation

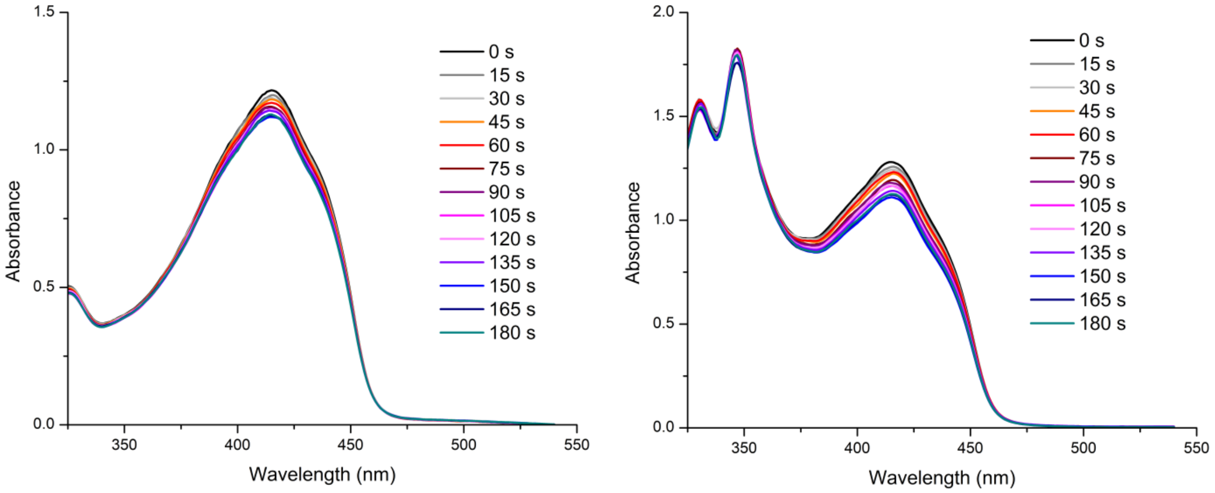

1 2

Figure S33. UV-Vis absorption spectra of diphenylisobenzofuran (DPBF) in DMSO (concentration: 50 μM) treated with the complexes **1** and **2** (concentration: 50 μM) upon exposure to visible light at 420 nm recorded over 180 s.

Triplet–triplet annihilation upconversion

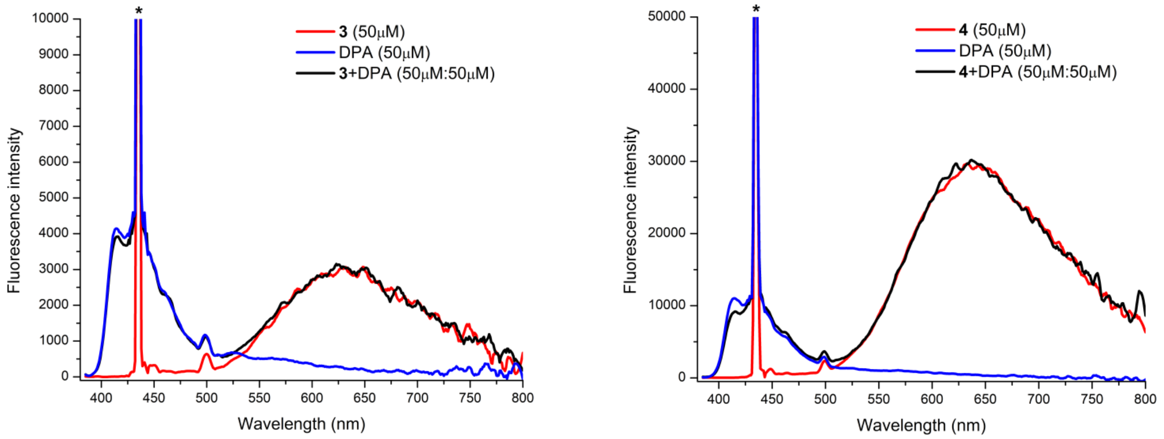

3 4

Figure S34. Emission spectra displaying TTA upconversion of 9,10-diphenylanthracene (DPA) in presence of complexes **3** and **4**. Asterisk denotes excitation wavelength of 435 nm.

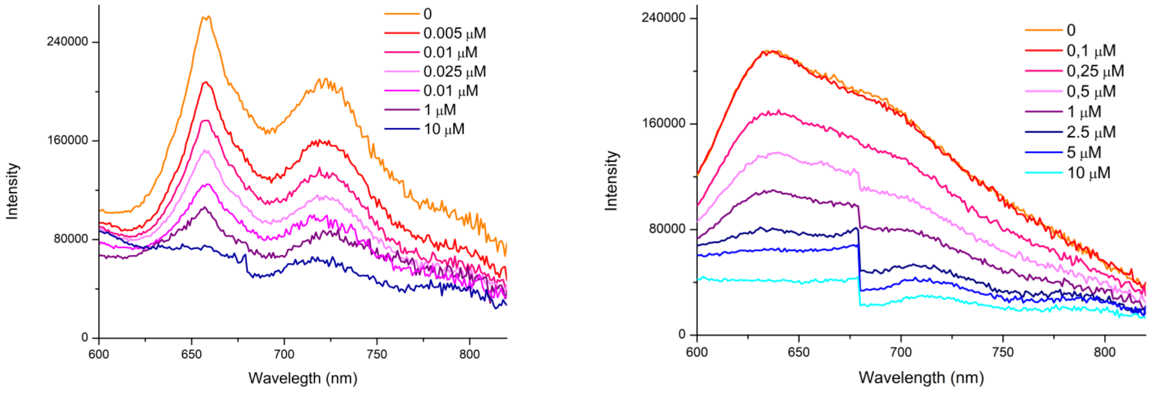

1 **2**

(a)

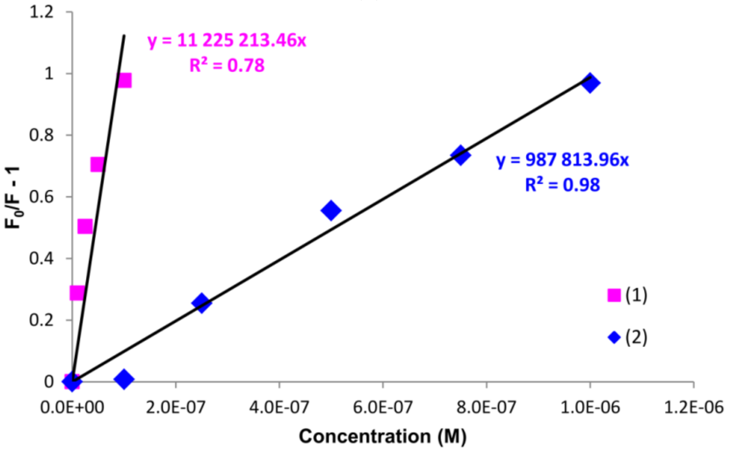

(b)

Figure S35. The phosphorence quenching of the complexes **1** and **2** with increasing concentration of DPA in deaerated DMSO (a). Stern-Volmer plots of $(F_0/F)–1$ vs DPA concentration for complexes **1** and **2** (b). F and F_0 are integrated intensities of the Re(I) complexes phosphorescence with and without DPA.

References

(1) Mariappan, M.; Maiya, B. G. Effects of Anthracene and Pyrene Units on the Interactions of Novel Polypyridylruthenium(II) Mixed-Ligand Complexes with DNA. *European Journal of Inorganic Chemistry* **2005**, *2005* (11), 2164–2173. https://doi.org/10.1002/ejic.200400952.

(2) Peuntinger, K.; Pilz, T. D.; Staehle, R.; Schaub, M.; Kaufhold, S.; Petermann, L.; Wunderlin, M.; Görls, H.; Heinemann, F. W.; Li, J.; Drewello, T.; Vos, J. G.; Guldi, D. M.; Rau, S. Carbene Based Photochemical Molecular Assemblies for Solar Driven Hydrogen Generation. *Dalton Trans.* **2014**, *43* (36), 13683–13695. https://doi.org/10.1039/C4DT01546K.

(3) Singh, V.; Mondal, P. C.; Kumar, A.; Jeyachandran, Y. L.; Awasthi, S. K.; Gupta, R. D.; Zharnikov, M. Surface Confined Heteroleptic Copper(II)–Polypyridyl Complexes for Photonuclease Activity. *Chem. Commun.* **2014**, *50* (78), 11484–11487. https://doi.org/10.1039/C4CC05063K.

(4) Jin, C.; Liu, J.; Chen, Y.; Li, G.; Guan, R.; Zhang, P.; Ji, L.; Chao, H. Cyclometalated Iridium(III) Complexes with Imidazo[4,5-f][1,10]Phenanthroline Derivatives for Mitochondrial Imaging in Living Cells. *Dalton Trans.* **2015**, *44* (16), 7538–7547. https://doi.org/10.1039/C5DT00467E.

(5) Szłapa-Kula, A.; Palion-Gazda, J.; Ledwon, P.; Erfurt, K.; Machura, B. A Fundamental Role of the Solvent Polarity and Remote Substitution of the 2-(4-R-Phenyl)-1H-Imidazo[4,5-

f][1,10]Phenanthroline Framework in Controlling the Ground- and Excited-State Properties of Re(I) Chromophores [ReCl(CO)3(R-C6H4-Imphen)]. *Dalton Trans.* **2022**, *51* (38), 14466–14481. https://doi.org/10.1039/D2DT02439J.

(6) CrysAlis PRO, Oxford Diffraction/Agilent Technologies UK Ltd, Yarnton, England, 2014.

(7) Sheldrick, G. M. Crystal Structure Refinement with SHELXL. *Acta Cryst C* **2015**, *71* (1), 3–8. https://doi.org/10.1107/S2053229614024218.

(8) Frisch, M. J.; Trucks, G. W.; Schlegel, H. B.; Scuseria, G. E.; Robb, M. A.; Cheeseman, J. R.; Scalmani, G.; Barone, V.; Petersson, G. A.; Nakatsuji, H.; Li, X.; Caricato, M.; Marenich, A. V.; Bloino, J.; Janesko, B. G.; Gomperts, R.; Mennucci, B.; Hratchian, H. P.; Ortiz, J. V.; Izmaylov, A. F.; Sonnenberg, J. L.; Williams; Ding, F.; Lipparini, F.; Egidi, F.; Goings, J.; Peng, B.; Petrone, A.; Henderson, T.; Ranasinghe, D.; Zakrzewski, V. G.; Gao, J.; Rega, N.; Zheng, G.; Liang, W.; Hada, M.; Ehara, M.; Toyota, K.; Fukuda, R.; Hasegawa, J.; Ishida, M.; Nakajima, T.; Honda, Y.; Kitao, O.; Nakai, H.; Vreven, T.; Throssell, K.; Montgomery Jr., J. A.; Peralta, J. E.; Ogliaro, F.; Bearpark, M. J.; Heyd, J. J.; Brothers, E. N.; Kudin, K. N.; Staroverov, V. N.; Keith, T. A.; Kobayashi, R.; Normand, J.; Raghavachari, K.; Rendell, A. P.; Burant, J. C.; Iyengar, S. S.; Tomasi, J.; Cossi, M.; Millam, J. M.; Klene, M.; Adamo, C.; Cammi, R.; Ochterski, J. W.; Martin, R. L.; Morokuma, K.; Farkas, O.; Foresman, J. B.; Fox, D. J. Gaussian 16 Rev. C.01, 2016.

(9) Adamo, C.; Barone, V. Toward Reliable Density Functional Methods without Adjustable Parameters: The PBE0 Model. *J. Chem. Phys.* **1999**, *110* (13), 6158–6170. https://doi.org/10.1063/1.478522.

(10) Ernzerhof, M.; Scuseria, G. E. Assessment of the Perdew–Burke–Ernzerhof Exchange-Correlation Functional. *The Journal of Chemical Physics* **1999**, *110* (11), 5029–5036. https://doi.org/10.1063/1.478401.

(11) Andrae, D.; Häußermann, U.; Dolg, M.; Stoll, H.; Preuß, H. Energy-Adjustedab Initio Pseudopotentials for the Second and Third Row Transition Elements. *Theoret. Chim. Acta* **1990**, *77* (2), 123–141. https://doi.org/10.1007/BF01114537.

(12) Weigend, F.; Ahlrichs, R. Balanced Basis Sets of Split Valence, Triple Zeta Valence and Quadruple Zeta Valence Quality for H to Rn: Design and Assessment of Accuracy. *Phys. Chem. Chem. Phys.* **2005**, *7* (18), 3297–3305. https://doi.org/10.1039/B508541A.

(13) Rappoport, D.; Furche, F. Property-Optimized Gaussian Basis Sets for Molecular Response Calculations. *J. Chem. Phys.* **2010**, *133* (13), 134105. https://doi.org/10.1063/1.3484283.

(14) Cancès, E.; Mennucci, B.; Tomasi, J. A New Integral Equation Formalism for the Polarizable Continuum Model: Theoretical Background and Applications to Isotropic and Anisotropic Dielectrics. *J. Chem. Phys.* **1997**, *107* (8), 3032–3041. https://doi.org/10.1063/1.474659.

(15) Mennucci, B.; Tomasi, J. Continuum Solvation Models: A New Approach to the Problem of Solute's Charge Distribution and Cavity Boundaries. *J. Chem. Phys.* **1997**, *106* (12), 5151–5158. https://doi.org/10.1063/1.473558.

(16) Cossi, M.; Barone, V.; Mennucci, B.; Tomasi, J. Ab Initio Study of Ionic Solutions by a Polarizable Continuum Dielectric Model. *Chemical Physics Letters* **1998**, *286* (3–4), 253–260. https://doi.org/10.1016/S0009-2614(98)00106-7.

(17) Szlapa-Kula, A.; Małecka, M.; Maroń, A. M.; Janeczek, H.; Siwy, M.; Schab-Balcerzak, E.; Szalkowski, M.; Maćkowski, S.; Pedzinski, T.; Erfurt, K.; Machura, B. In-Depth Studies of Ground- and Excited-State Properties of Re(I) Carbonyl Complexes Bearing 2,2′:6′,2″-Terpyridine and 2,6-Bis(Pyrazin-2-Yl)Pyridine Coupled with π-Conjugated Aryl Chromophores. *Inorg. Chem.* **2021**, *60* (24), 18726–18738. https://doi.org/10.1021/acs.inorgchem.1c02151.

(18) Małecka, M.; Szlapa-Kula, A.; Maroń, A. M.; Ledwon, P.; Siwy, M.; Schab-Balcerzak, E.; Sulowska, K.; Maćkowski, S.; Erfurt, K.; Machura, B. Impact of the Anthryl Linking Mode on the Photophysics and Excited-State Dynamics of Re(I) Complexes [ReCl(CO)3(4′-An-Terpy-κ2N)]. *Inorg. Chem.* **2022**, *61* (38), 15070–15084. https://doi.org/10.1021/acs.inorgchem.2c02160.

(19) Wilderen, L. J. G. W. van; Lincoln, C. N.; Thor, J. J. van. Modelling Multi-Pulse Population Dynamics from Ultrafast Spectroscopy. *PLOS ONE* **2011**, *6* (3), e17373. https://doi.org/10.1371/journal.pone.0017373.

(20) Slavov, C.; Hartmann, H.; Wachtveitl, J. Implementation and Evaluation of Data Analysis Strategies for Time-Resolved Optical Spectroscopy. *Anal. Chem.* **2015**, *87* (4), 2328–2336. https://doi.org/10.1021/ac504348h.
(21) Lutkus, L. V.; Rickenbach, S. S.; McCormick, T. M. Singlet Oxygen Quantum Yields Determined by Oxygen Consumption. *Journal of Photochemistry and Photobiology A: Chemistry* **2019**, *378*, 131–135. https://doi.org/10.1016/j.jphotochem.2019.04.029.
(22) Yuan, Z.; He, J.; Mahmood, Z.; Xing, L.; Ji, S.; Huo, Y.; Zhang, H.-L. Deciphering the Ligand's Geometric Effect on the Photophysical Properties of Osmium Complex and Its Application in Triplet-Triplet Annihilation Upconversion. *Dyes and Pigments* **2022**, *199*, 110049. https://doi.org/10.1016/j.dyepig.2021.110049.
(23) Singh-Rachford, T. N.; Islangulov, R. R.; Castellano, F. N. Photochemical Upconversion Approach to Broad-Band Visible Light Generation. *J. Phys. Chem. A* **2008**, *112* (17), 3906–3910. https://doi.org/10.1021/jp712165h.
(24) Zhao, J.; Ji, S.; Guo, H. Triplet–Triplet Annihilation Based Upconversion: From Triplet Sensitizers and Triplet Acceptors to Upconversion Quantum Yields. *RSC Adv.* **2011**, *1* (6), 937–950. https://doi.org/10.1039/C1RA00469G.
